# Hypochlorous Acid-Gated Hydrolysis of a Phosphinate Ester Dye in Living Cells

**DOI:** 10.1021/jacs.5c12615

**Published:** 2025-10-22

**Authors:** Yuan Fang, Xinqi Zhou, Julia L. McAfee, Benjamin M. Faulkner, Lauren Lesiak, Yuchen He, Frederik Bro̷ndsted, Hao Fan, Eric D. Donarski, Xiaoyan Hu, B. Jill Venton, Steven Grant, Francine E. Garrett-Bakelman, Cliff I. Stains

**Affiliations:** † Department of Chemistry, 2358 University of Virginia, Charlottesville, Virginia 22904, United States; ‡ Department of Chemistry, University of Nebraska-Lincoln, Lincoln, Nebraska 68588, United States; § Department of Medicine, 2358 University of Virginia, Charlottesville, Virginia 22904, United States; ∥ Department of Biochemistry and Molecular Genetics, 2358 University of Virginia, Charlottesville, Virginia 22904, United States; ⊥ Division of Hematology/Oncology, Department of Medicine, 6887 Virginia Commonwealth University, Richmond, Virginia 23298, United States; # Massey Cancer Center, 6887 Virginia Commonwealth University, Richmond, Virginia 23298, United States; ∇ University of Virginia Cancer Center, 2358 University of Virginia, Charlottesville, Virginia 22908, United States; ○ Virginia Drug Discovery Consortium, Blacksburg, Virginia 24061, United States

## Abstract

Theranostic fluorescent platforms are capable of the selective delivery of small molecules to target cells with simultaneous optical monitoring. Such technologies promise to significantly reduce off-target effects compared with cytotoxic chemotherapy. However, small-molecule approaches are often hindered by relatively complex designs that are required to incorporate a fluorescent reporter, reactive linker, targeting ligand, and cargo into a single molecule. Herein, we provide the first direct evidence for the ability to gate the delivery of small-molecule cargos from phosphinate ester-containing Nebraska Red (**NR**) dyes in vitro and in living cells. This simplified system integrates the fluorescent reporter, reactive linker, and targeting ligand into one species—a phosphinate ester dye. As a proof-of-principle for delivery of drug-like molecules to cells, we developed **NR-HOCl-TFMU**, which responds to hypochlorous acid (HOCl), an analyte detected in acute myeloid leukemia (AML). **NR-HOCl-TFMU** is stable for days prior to reaction with HOCl, leading to phosphinate ester hydrolysis and production of a NIR (near-infrared, **NR** dye) and blue (cargo) fluorescence signal. **NR** dye fluorescence is directly proportional to cargo release, and **NR-HOCl-TFMU** is capable of selectively delivering its drug-like, small-molecule cargo to AML cells in vitro and in a localized tumor model in an HOCl-gated manner. In the long term, we envision the potential use of this technology to afford HOCl-gated delivery systems with selectivity toward HOCl-positive AML cells. More broadly, this approach provides a potentially generalizable strategy for the development of simplified theranostic agents targeted toward small-molecule analytes and enzymatic activities associated with disease.

## Graphical abstract

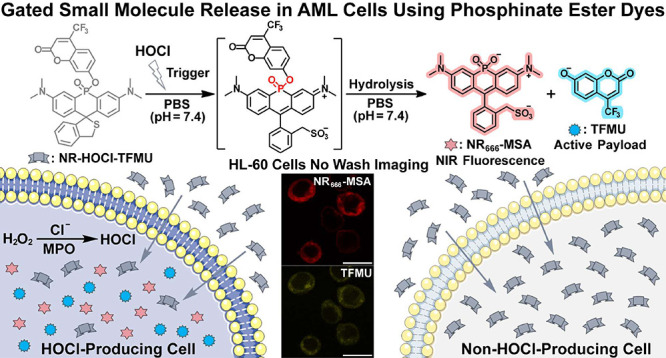

## Introduction

Cytotoxic chemotherapy does not effectively distinguish between healthy and malignant cells, leading to severe off-target effects that significantly reduce patient quality of life and, in some cases, can lead to treatment-related morbidity or mortality.^1,2^ Several fluorophore-based prodrug strategies have been explored to mitigate these off-target issues and enable the direct delivery of cytotoxic agents to malignant cells.^1–3^ In general, these approaches involve masking drug activity through conjugation to a fluorescent reporter and targeting a ligand via a reactive linker, enabling selective uptake in malignant cells (Figure 1a). Upon reaction with intracellular stimuli (glutathione, pH, reactive oxygen species [ROS], thiols, enzymatic activities, etc.) or exogenous stimuli (light, bioorthogonal catalysts, ligands, etc.), the active cargo is released from the conjugate.^1–3^ These theranostic agents can provide imaging data to confirm the delivery of cytotoxic drugs to malignant cells and enable monitoring of therapeutic outcomes (Figure 1a). Although theranostic agents are potentially powerful tools for precision medicine, the integration of a targeting ligand, an imaging agent, a reactive linker, and a therapeutic compound into a single reagent results in relatively complex structures with large molecular weights. Thus, strategies that could decrease the molecular weight of theranostics are desirable in order to improve their performance (e.g., cellular uptake). Moreover, due to the relatively blue-shifted fluorescence of dyes commonly used in theranostics, deep-tissue imaging is not possible. Theranostic approaches utilizing NIR (650–900 nm) fluorophores, which provide deeper tissue penetration, represent an important area for growth in the field.^1,2,4–6^

**Figure 1:**
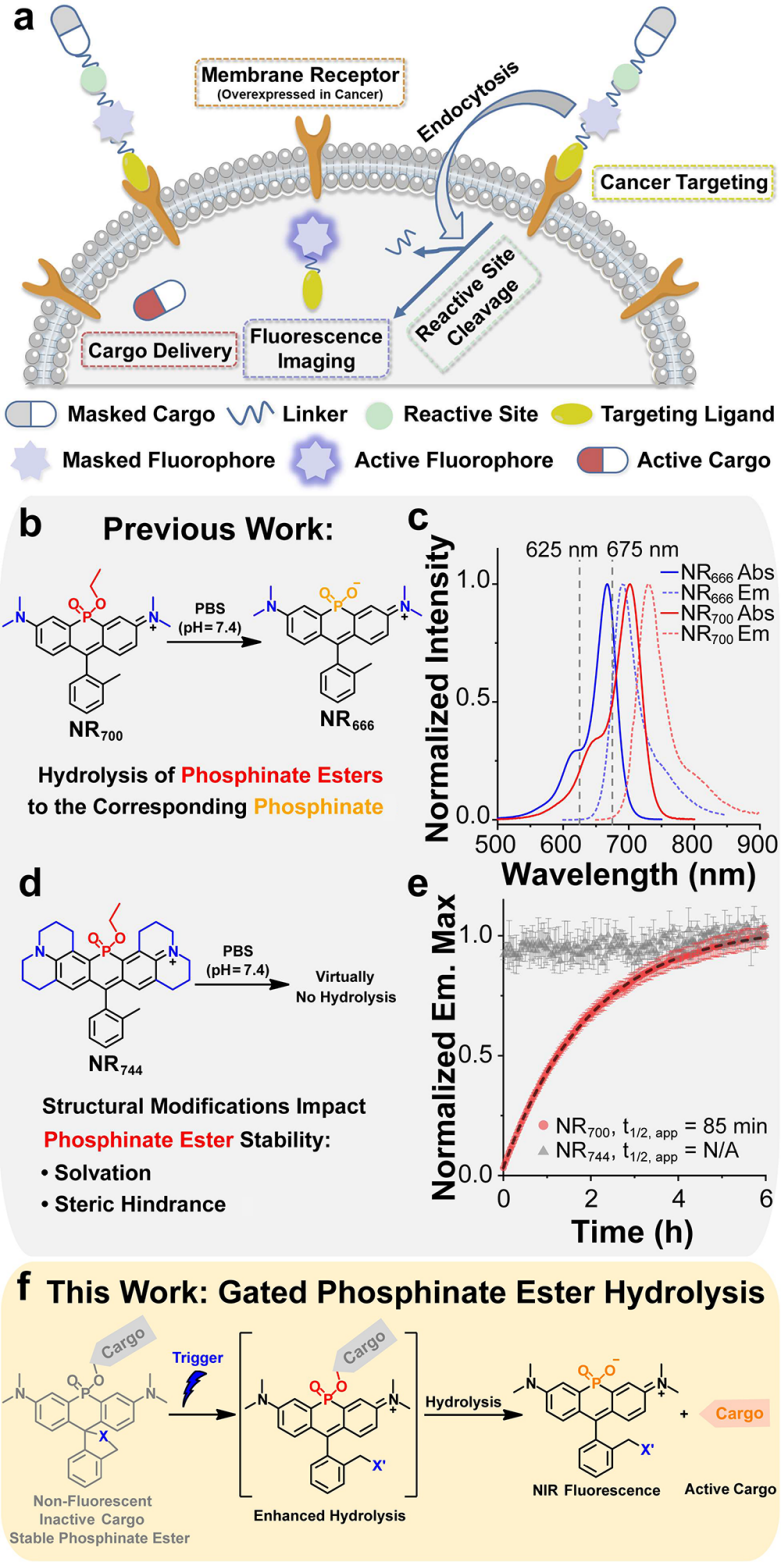
Gated hydrolysis of phosphinate ester-containing dyes for delivery of small molecules. (a) General design strategy for theranostic fluorescent probes. (b) Hydrolysis of the phosphinate ethyl ester-containing dye **NR**_**700**_ to form the corresponding phosphinate (**NR**_**666**_) in PBS. (c) Normalized absorbance and emission spectra of **NR**_**700**_ and **NR**_**666**_ in PBS (pH = 7.4 with 1% DMSO). (d) Structure of an **NR** phosphinate ester analog of rhodamine 101 (**NR**_**744**_). (e) Structural modifications to the **NR** dye core influence the rate of phosphinate ester hydrolysis. Comparison of the hydrolysis rate of 5 μM **NR**_**700**_ or **NR**_**744**_ in PBS (pH = 7.4 with 1% DMSO) by measuring the formation of the corresponding hydrolyzed product **NR**_**666**_ (Ex: 625 nm, Em: 675 nm) or **NR**_**698**_ (Ex: 675 nm, Em: 720 nm). (f) Leveraging gated hydrolysis of phosphinate ester-containing **NR** dyes for delivery of small-molecule cargos.

Our group has previously reported a strategy to obtain NIR-fluorescent rhodamine dyes by replacement of the oxygen atom at the 10-position with a phosphinate ester functional group.^7,8^ This modification results in a significant red shift in both the absorbance and emission spectra of the resulting dyes (∼110 nm), yielding probes with NIR fluorescence. Subsequently, we have shown that phosphinate esters can also induce significant red shifts in the absorbance and fluorescence of fluorescein, as well as methylene blue scaffolds; collectively, we have termed this new dye class **Nebraska Red** (**NR**) dyes.^7–10^ Interestingly, we previously observed rapid hydrolysis of an **NR** derivative bearing a phosphinate ethyl ester (**NR**_**700**_) to the corresponding phosphinate (**NR**_**666**_) at physiological pH (Figure 1b). The relatively electron-rich phosphinate formed after hydrolysis results in a blue shift in the absorbance and emission spectrum of **NR**_**666**_ by ∼30 nm compared to **NR**_**700**_, allowing for the direct observation of **NR**_**666**_ formation (Figure 1c).^7,11^ In subsequent work, we synthesized a series of **NR** rhodamine dyes and discovered that structural modifications can substantially impact the rates of phosphinate ester hydrolysis in the resulting dyes.^7,8,11,12^ For example, the hydrolysis of a rhodamine 101 derivative (**NR**_**744**_, Figure 1d) was virtually undetectable at physiological pH (Figure 1d,e).^12^ Indeed, **NR**_**744**_ required reflux in 6 M HCl to obtain the corresponding hydrolyzed product **NR**_**698**_ (Figure S1).^7^ These results are in agreement with previous work by Haake and co-workers, demonstrating that the hydrolysis of phosphinate esters is a second-order reaction involving both the phosphinate ester and hydroxide anion.^13^ Importantly, this work demonstrated that the rate-limiting step of phosphinate ester hydrolysis is the collapse of the pentavalent intermediate formed after attack by the hydroxide anion (*k*_2_ in Figure S2). Further mechanistic analysis indicated that the steric hindrance of the pentavalent oxyanion intermediate to solvation (Figure S2) controls the rate of this reaction, and structural modifications that increase the solvation of the intermediate lead to increases in the rate of phosphinate ester hydrolysis and vice versa.^13^ Although we cannot completely rule out nucleophilic attack as the rate-limiting step in the context of all **NR** derivatives, our data is consistent with this previous work in that the increased steric bulk of the julolidine substituents in **NR**_**744**_ may prevent effective solvation of the corresponding pentavalent intermediate. Our lab is investigating the extent to which phosphinate ester hydrolysis can be modulated in **NR** dyes using structural modifications. Nonetheless, these current observations clearly indicate the potential to leverage structural changes to gate the hydrolysis of **NR** dyes.

Based on the above observations, we hypothesized that the rate of phosphinate ester hydrolysis in **NR** dyes could be gated by reactions that changed the overall charge, and therefore solvation, of the dye (Figure 1f). In the current design, reaction with the target analyte converts an uncharged, nonfluorescent starting material into a fluorescent, zwitterionic species, which displays enhanced phosphinate ester hydrolysis. Hydrolysis then results in the delivery of a small-molecule cargo (via phosphinate ester hydrolysis) and the production of a membrane-impermeable imaging probe. Such reagents integrate the targeting group, reactive linker, and imaging probe into one species, providing a simplified reagent for small-molecule delivery. Nonetheless, several important challenges must be overcome to realize this goal. First, the phenols present in drug-like molecules possess a lower p*K*_a_ than ethanol, which may decrease the stability of the resulting phosphinate esters and prohibit effective gating. Second, the direct visualization of cargo delivery would be desirable to validate such a system both in vitro and in cells. Lastly, the membrane permeability of complex phosphinate esters remains unknown. Herein, we address these issues and demonstrate for the first time that **NR** dyes can be used as a platform to deliver drug-like small-molecule cargo to living cells. We identify an optimal phenol p*K*_a_ (>7.2), which yields **NR** dye esters that are stable for days prior to reaction with a target analyte. Using this approach, we designed a system to selectively deliver small molecules to AML cells in response to aberrant HOCl production and demonstrated the potential for HOCl-activated cytotoxicity. Given the availability of spiro-ring opening strategies for a variety of disease-relevant analytes (e.g., ROS, reactive nitrogen species [RNS], formaldehyde, or heavy metals),^14–17^ we expect that this platform will enable the construction of diverse new precision medicine reagents for human disease.

## Results and Discussion

### Choice of the Gate for Small-Molecule Delivery

AML is the most common type of acute leukemia in US adults and is a genetically heterogeneous disease that can be defined using molecular events and results in the accumulation of immature myeloid stem cells (usually in the myeloblast or promyelocyte stage) in the bone marrow and peripheral blood.^18–20^ After a patient is diagnosed with AML, the standard of care is intensive combination chemotherapy with or without targeting agents, or less intense treatment options, followed by consolidation treatment.^21^ Although these approaches can produce complete remission in over 60% of patients <60 years old,^20^ 5-year survival rates remain less than 35%, and for patients older than 65 years of age, overall survival is less than 10%.^22^ This difference in age-related outcomes is important since the median age of diagnosis for AML is 69.^23–25^ One factor that contributes to this age-related difference in treatment outcomes is the significantly higher treatment-related morbidity in older individuals.^26^ Thus, there is a clear need to develop strategies to selectively deliver small molecules to AML cells with the long-term goal of developing precision medicine agents with reduced off-target effects to improve clinical outcomes for AML patients.

Interestingly, since AML cells are immature myeloid stem cells, they exhibit a unique feature that distinguishes them from normal cells on a biochemical level. More specifically, previous work has shown that myeloid stem cells begin to express myeloperoxidase (MPO) in azurophilic granules during differentiation and that AML cells often exhibit high levels of MPO, making expression of this enzyme a valuable diagnostic marker for AML in the clinic.^27,28^ Moreover, unrestrained production of HOCl, the enzymatic product of MPO, has been observed in established AML cell lines (e.g., HL-60, 1.03 μM) and primary myeloblasts (2.4–15.6 μM).^29^ HOCl is a potent ROS that plays a significant role in the innate immune response and inflammatory diseases.^30–33^ Normal myeloid cells begin expressing MPO in azurophilic granules during the early stages of myeloid differentiation, starting at the myeloid blast stage and peaking at the promyelocyte stage. This expression persists as the cells mature into fully developed granulocytes (e.g., neutrophils and macrophages), though at decreasing levels.^34–36^ During the innate immune response, granulocytes engulf pathogens and azurophilic granules fuse with the phagosome, leading to the release of MPO into the same cellular compartment as H_2_O_2_ and Cl^–^, substrates which are required for the enzymatic production of HOCl.^29,31–33^ Thus, in the healthy state, HOCl production is tightly regulated through the subcellular compartmentalization of MPO and its substrates. However, since many AML patients are neutropenic at diagnosis,^37–39^ aberrant production of HOCl in AML cells represents an underexplored trigger for development of imaging agents^11^ and small-molecule delivery platforms. In the long term, HOCl-activatable agents could significantly reduce off-target effects from treatment in AML, compared to cytotoxic chemotherapy, in patients with HOCl-positive myeloblasts.

To directly investigate the range of HOCl production in established AML cells, we employed **NR-HOCl** (Figure 2), a cell-permeable, turn-on NIR fluorescent probe, with high selectivity for HOCl in living cells.^7^ The turn-on fluorescence signal from NR-HOCl was used to quantify the relative production of HOCl in a panel of established AML cell lines (Figures S3 and S4),^40^ which was correlated to MPO expression level, as evaluated by Western blotting (Figure S5). These studies demonstrated that while MPO expression is required for HOCl generation, its expression level alone does not reliably predict the level of HOCl production (Figure 2), consistent with the observation that MPO activity is highly regulated in cells, in part, by access to substrates.^41,42^ These experiments indicate the potential utility of HOCl as a trigger for the activation of cytotoxins in AML cells. Furthermore, the lack of a correlation between MPO expression levels and HOCl production provides further motivation for our continued efforts to develop companion diagnostics for HOCl expression in the context of AML. Such companion diagnostics could be utilized to select HOCl-positive patients who might respond to HOCl-activated therapies. Given the potential to target AML cells using HOCl as a trigger, we chose to move forward with HOCl as our first target for construction of a small-molecule delivery system based on the **NR** dye scaffold.

**Figure 2:**
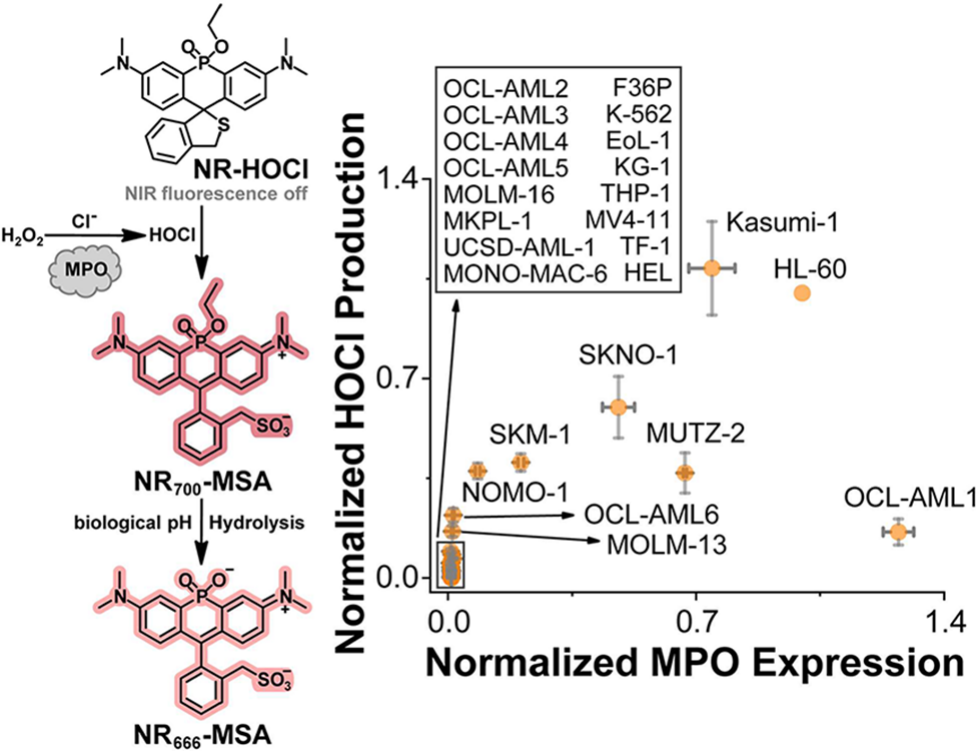
MPO expression does not correlate with HOCl production in established AML cell lines. Left: Schematic of the fluorescent turn-on mechanism for the previously published **NR-HOCl** turn-on fluorescent probe. Right: Flow-cytometry-based screening of AML cells using the previously reported NIR fluorescent probe **NR-HOCl**. The percentage of NIR-fluorescent cells does not correlate with MPO expression levels determined by Western blot across different AML cell lines. Data for both HOCl production (**NR-HOCl**) and MPO expression (Western blotting) are relative to HL-60 cells. *X*-axis error bars represent the standard deviation (SD) of three technical replicates, and *Y*-axis data are presented as mean ± SD from four technical replicates.

### Design, Synthesis, and In Vitro Evaluation of a HOCl-Activated Small-Molecule Delivery Platform

We next sought to identify a cargo that would mimic drug-like compounds while allowing for direct observation of delivery in living cells. Noting that several cytotoxic agents (such as microtubule polymerization inhibitors: combretastatin A-4,^5^ procaspase-3 activators: 1541B^43^ and PAC-1,^44^ and DNA alkylators: duocarmycin^6^) contain phenols that could possibly be used as points of attachment to **NR** dyes, we chose to investigate the ability to cage phenol-containing compounds. To facilitate the monitoring of cargo release while mimicking the molecular complexity of cytotoxins, we turned our attention to fluorescent dyes containing phenols, such as umbelliferone,^45^ resorufin,^46^ and NBD.^47^ Importantly, these dyes contain phenols of varying p*K*_a_s, allowing for the analysis of the effect of phenol p*K*_a_ on phosphinate ester stability. In addition, their fluorescence is dependent upon deprotonation of these phenol groups, enabling the design of turn-on agents to directly visualize cargo delivery. We selected 4-methylumbelliferone (**4MU**) as our first observable cargo and envisioned the development of **NR-HOCl-4MU** (Figure 3a), an HOCl-activatable agent that would produce both a NIR fluorescence (**NR** dye) and blue fluorescence (**4MU**) readout after the reaction with HOCl. Gratifyingly, **NR-HOCl-4MU** could be readily obtained in two steps by first refluxing **NR-HOCl** in 6 M hydrochloric acid and then employing CDI coupling to produce the corresponding phosphinate ester analog of **NR**_**666**_ **-HOCl** (Figure 3a).^7^ This reaction was highly efficient and preserved the spirocyclic thioether recognition element for HOCl (yield: 92%). **4MU** was then installed through CDI coupling,^48^ after which the product was isolated and purified through HPLC to yield **NR-HOCl-4MU** (yield: 78%).

**Figure 3:**
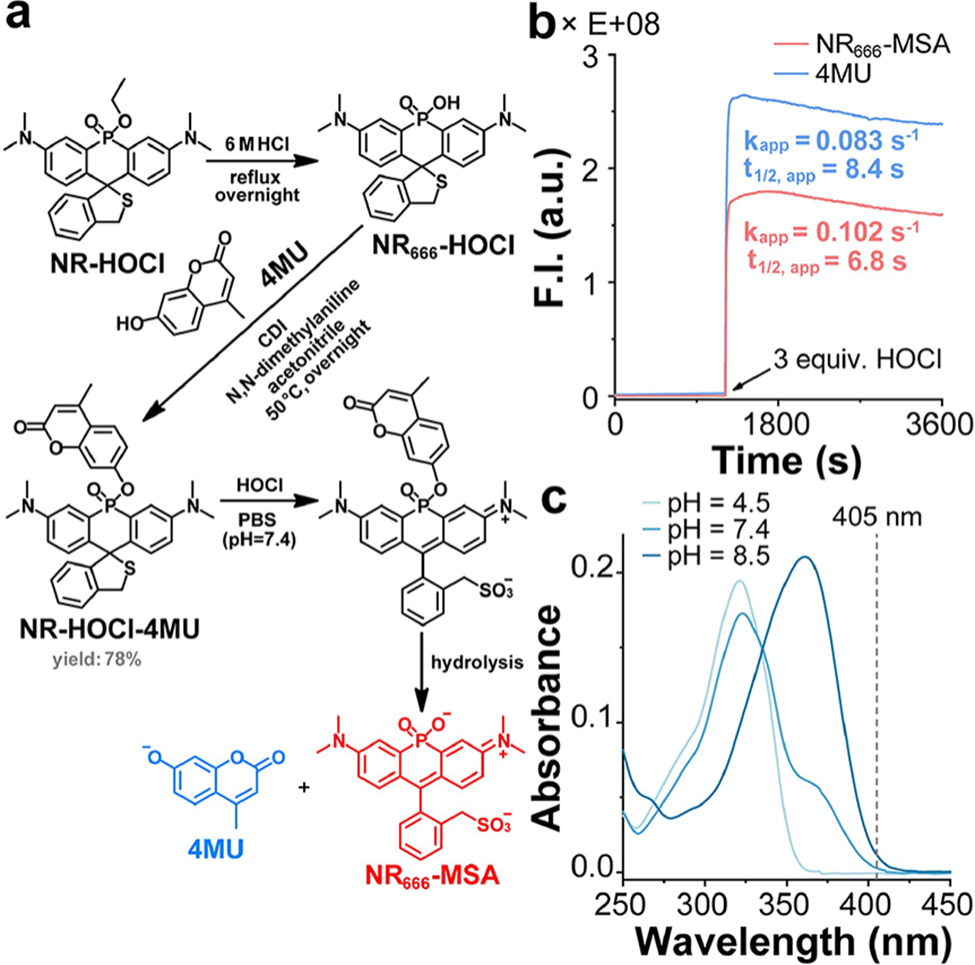
Direct visualization of gated cargo release from phosphinate ester dyes in vitro. (a) Synthetic route for **NR-HOCl-4MU** and its reaction with HOCl to form **NR**_**666**_ **-MSA** and **4MU**. (b) Time-dependent formation of **NR**_**666**_ **-MSA** (Ex: 666 nm, Em: 698 nm) and **4MU** (Ex: 365 nm, Em: 447 nm) after adding 3 equiv. of HOCl to **NR-HOCl-4MU** (5 μM) in PBS (pH = 7.4 with 1% DMF). (c) UV–vis absorbance spectra of **4MU** (10 μM) at the indicated pH (with 1% DMSO).

We initially tested the fluorescence of **NR-HOCl-4MU** (5 μM) in PBS (pH = 7.4 with 1% DMF) in the presence or absence of HOCl (Figure 3b). In the absence of HOCl, there was no increase in either NIR (698 nm) or blue (447 nm) fluorescence within 20 min, confirming that **NR-HOCl-4MU** was stable prior to reaction with HOCl. Upon addition of HOCl (3 equiv), we observed a robust fluorescence turn-on signal in both the NIR (510-fold) and blue (120-fold) fluorescence channels (Figure 3b), corresponding to the generation of both **NR**_**666**_ **-MSA** and **4MU** (Figure 3a). After the reaction with HOCl, both the NIR and blue fluorescence intensities were relatively stable over time (Figure 3b). This phenomenon was also observed during sequential additions of 0.5 equiv of HOCl to **NR-HOCl-TFMU** (Figure S6). The fluorescence enhancements upon addition of HOCl could be fit using an exponential equation to yield apparent first-order rates of hydrolysis of *k*_app_ = 0.102 s^–1^ (698 nm) and 0.083 s^–1^ (447 nm) for **NR**_**666**_ **-MSA** and **4MU**, respectively, indicating similar apparent rates of formation of each dye under these conditions (Figure 3b). The findings presented above indicate that small-molecule cargo release can ultimately be monitored using NIR fluorescence as a proxy and validate the ability to release more complex molecules from phosphinate ester dyes by leveraging chemical reactions that change the charge of the molecule.

While practical for in vitro assays, the use of **4MU** as a cargo hinders direct visualization of small-molecule delivery in living cells. For example, under neutral and alkaline pH conditions, deprotonation of the phenolic hydroxyl group of **4MU** induces a red shift in its absorbance wavelength (Figure 3c) and yields stronger fluorescence emission (Figure S7a). Even so, **4MU** (excitation wavelength = 365 nm) is not spectrally matched to the 405 nm UV laser line that is commonly equipped on confocal fluorescence microscopes. Thus, due to cellular autofluorescence (Figure S7b,c), the ability to visualize delivery of **4MU** from **NR-HOCl-4MU** in cells is severely hindered. Consequently, we sought to both identify cargos that were compatible with cellular imaging and investigate the influence of phenol p*K*_a_ on phosphinate ester stability in order to define the optimal characteristics for phenol-containing cargos in our system.

### Screening Cargos with Different Phenol p*K*_a_s

We set out to screen a small panel of phenol-containing cargo fluorophores with the goal of defining optimal phenol p*K*_a_s for the cargo as well as identifying a cargo that could be compatible with confocal imaging. Three additional fluorophores with red-shifted absorbance and emission wavelengths were chosen: 4-(trifluoromethyl)­umbelliferone (**TFMU**), 4-hydroxy-7-nitrobenzoxadiazole (**NBD-OH**), and **Resorufin** (Figure S8a). Importantly, these dyes are spectrally orthogonal to **NR**_**666**_ **-MSA** (Figure S8b,c), enabling resolution of both cargo and **NR** dye fluorescence during subsequent experiments. By adopting the same synthetic strategy used for **NR-HOCl-4MU** (Figure 3a), the three newly selected fluorophores were conjugated to **NR**_**666**_ **-HOCl** via CDI coupling (Figure S9). Using this approach, we achieved a 73% yield for **NR-HOCl-TFMU**. However, this strategy did not yield appreciable coupling product for **NBD-OH**, and we abandoned it as a cargo. **NR-HOCl-Resorufin** could be purified by HPLC as a single peak. However, characterization after lyophilization by ^1^H- and ^31^P NMR spectra indicated the presence of both **NR-HOCl-Resorufin** and **NR**_**666**_ **-HOCl** (Figure S10a), implying potential decomposition of the phosphinate ester during lyophilization. To verify this, an analytical HPLC injection was conducted prior to lyophilization, demonstrating a single peak for **NR-HOCl-Resorufin** (Figure S10b). After lyophilization, additional peaks were clearly observed that corresponded to **Resorufin** and **NR**_**666**_ **-HOCl** (Figure S10b), consistent with the NMR observations. Additional analysis of the absorbance spectrum of **NR-HOCl-Resorufin** in PBS (pH = 7.4) showed clear evidence for the presence of free **Resorufin** (Figure S10c). After mixing with HOCl, we observed the formation of **NR**_**666**_ **-MSA**; however, the absorbance of **Resorufin** did not change appreciably, indicating virtually complete hydrolysis of this phosphinate ester prior to treatment with HOCl.

In combination with our previous work (Figure 1e), the results presented above indicate that the p*K*_a_ of the phenol cargo influences cargo delivery rates and phosphinate ester stability. To further define the optimal p*K*_a_ for phenol-containing cargo, we measured the p*K*_a_s of cargo fluorophores via fluorescence (Figure S11). This analysis revealed p*K*_a_ values for **4MU**, **TFMU**, **NBD-OH**, and **Resorufin** of 7.76, 7.27, 2.20, and 5.89, respectively. These observations provide insights into the optimal p*K*_a_ for phenol-containing cargos. For example, the relatively low p*K*_a_ of **NBD-OH** (2.20) may yield phosphinate esters that are so unstable that they cannot be purified. The higher p*K*_a_ value of **Resorufin** (5.89) allowed for isolation of the corresponding phosphinate ester. However, this phosphinate ester did not survive lyophilization in the HPLC buffer (Figure S10). Lastly, phosphinate esters containing **4MU** (7.76) and **TFMU** (7.27) could be isolated, and their hydrolysis could be effectively gated with HOCl (e.g., Figure 3). Moreover, virtually, no phosphinate ester hydrolysis of **NR-HOCl-TFMU** was observed in DMF stocks over the course of six months (see the Supplementary Methods section). Thus, in the context of **NR**_**666**_ **-HOCl** (a tetramethyl rhodamine analog), we define a p*K*_a_ of >7.2 for phenol-containing cargo as optimal. Other strategies, such as modification of the dye core (e.g., **NR**_**744**_; see Figures 1d,e and S1) or the use of self-immolative linkers,^49^ could facilitate the use of phenol cargo with lower p*K*_a_s. These approaches are currently being investigated by our lab. Considering the apparent stability of **NR-HOCl-TFMU** and the ability to directly visualize delivery of this cargo using confocal microscopy, we chose to move forward with characterization of **NR-HOCl-TFMU** in vitro and in living cells.

### HOCl-Gated Cargo Delivery from NR-HOCl-TFMU In Vitro

The results above and previous mechanistic analysis (Figure S2) indicate that phosphinate ester stability is dependent upon pH.^13^ Therefore, we initially assayed **NR-HOCl-TFMU** for the formation of **NR**_**666**_ **-MSA** and **TFMU** before and after the addition of HOCl in Britton–Robinson buffer with pH ranging from 2 to 10 (Figure S12). In the absence of HOCl, we observed no appreciable fluorescence from **NR**_**666**_ **-MSA**, indicating that the thioether gate in **NR-HOCl-TFMU** is insensitive to pH changes (Figure S12a). At pHs above 7, we observed a relatively weak increase in the fluorescence signal from **TFMU** (Figure S12b). This result is consistent with the bimolecular reaction kinetics of phosphinate esters and hydroxide (Figure S2). However, upon addition of HOCl, we observed a substantial turn-on fluorescence signal for both **NR**_**666**_ **-MSA** and **TFMU** in the biologically relevant pH range (>5, Figure S12). At lower pH values (3–5), the fluorescence of **NR**_**666**_ **-MSA** and **TFMU** did not increase significantly in the presence of HOCl. This result may be due to the reduced reaction of the spirocyclic thioether and/or lower hydrolysis rates of phosphinate esters at acidic pHs (Figure S2). Overall, these results show that **NR-HOCl-TFMU** is stable across the biologically relevant pH window prior to reaction with HOCl.

Next, we turned our attention toward characterizing the production of **NR**_**666**_ **-MSA** and **TFMU** from **NR-HOCl-4MU** upon reaction with HOCl (Figure 4a). Initial assays indicated that **NR-HOCl-4MU** remained intact until the reaction with HOCl, which resulted in a dramatic increase in fluorescence from both **NR**_**666**_ **-MSA** (698 nm) and **TFMU** (510 nm) that remained stable over time (Figure 4b,c). The apparent first-order reaction rates for the generation of **NR**_**666**_ **-MSA** and **TFMU** under these conditions were 0.077 and 0.066 s^–1^, respectively (Figure 4b), indicating that **TFMU** release occurred rapidly after the reaction with HOCl. Monitoring the formation of either **NR**_**666**_ **-MSA** or **TFMU** over time in the presence of HOCl using absorbance (Figure S13) or fluorescence (Figures S14 and 4d) showed an increase in both products up to 4.5 equiv of HOCl. Importantly, these results also imply that the extent of cargo delivery can be inferred from the fluorescence intensity of **NR**_**666**_ **-MSA**, which will be useful to monitor cargo delivery and to identify off-target effects in future efforts focused on drug delivery. At 4.5 equiv of HOCl, the fluorescence of both **NR**_**666**_ **-MSA** and **TFMU** plateaued with 1646- and 18-fold increases, respectively. To ensure that this plateau effect was not due to local bleaching of dyes upon addition of small volumes of highly concentrated HOCl to the reactions, this experiment was repeated by first preparing HOCl stocks at various concentrations and mixing equal volumes of these stocks with **NR-HOCl-TFMU** (Figure S15). Results from the experiment were consistent with those obtained in Figure 4d. To further investigate the trigger–release mechanism, we performed analytical HPLC separation and mass spectrometric analysis of the reaction mixture of **NR-HOCl-TFMU** and HOCl (Figure S16). Several key peaks were detected and identified, including an oxidized intermediate **NR-HOCl-TFMU**, the unhydrolyzed intermediate **NR**_**700**_ **-MSA-TFMU**, and the hydrolysis products **NR**_**666**_ **-MSA** and **TFMU** (Figure S16), consistent with our proposed reaction mechanism (Figures 1f and S2). Furthermore, the sensitivity of **NR-HOCl-TFMU** for HOCl was determined by **NR**_**666**_ **-MSA** (NIR channel, LOD = 7.1 nM) and **TFMU** (LOD = 31.0 nM) fluorescence (Figure S17), demonstrating comparable sensitivity to previously reported HOCl probes.^17,50^

**Figure 4:**
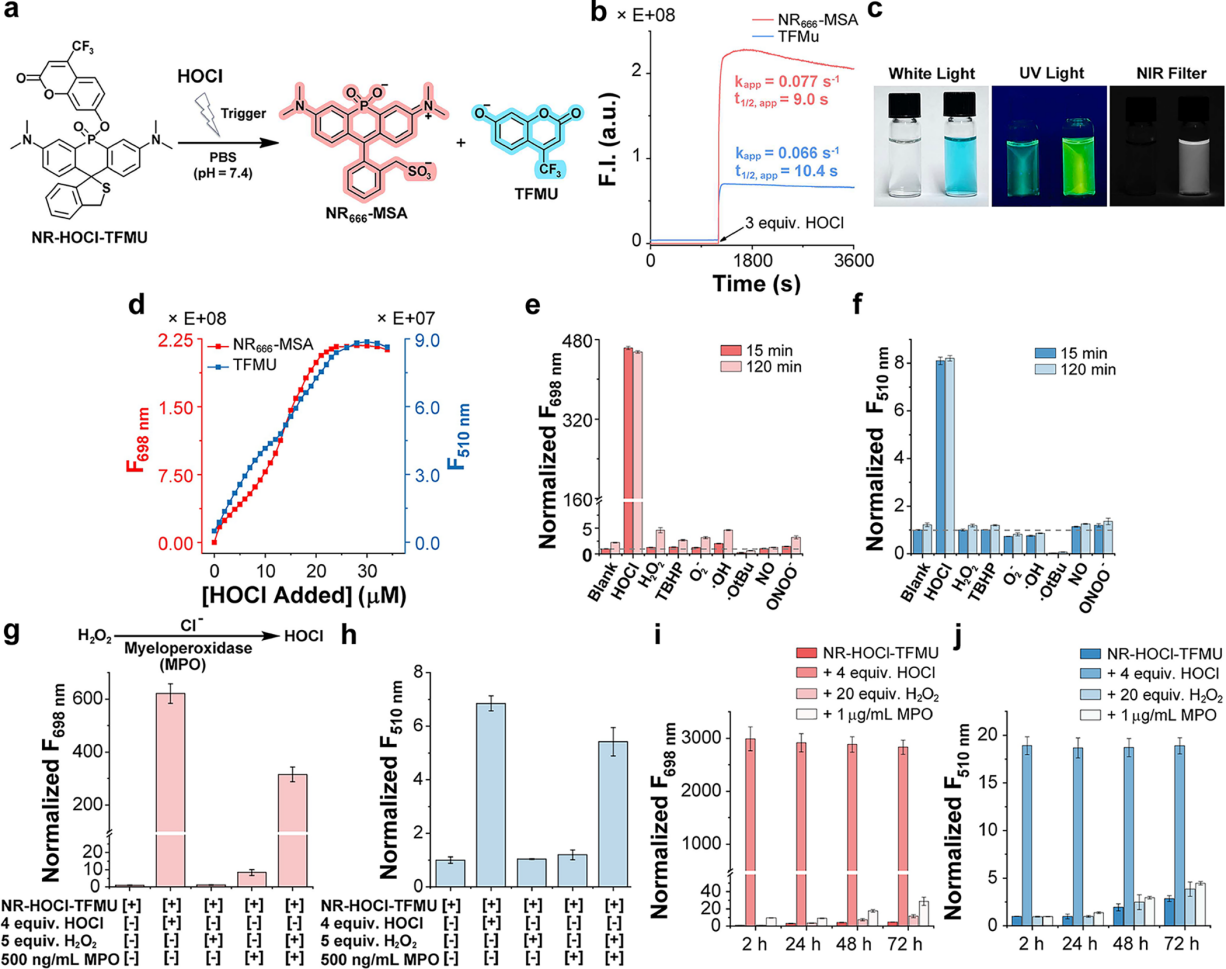
Gated hydrolysis of **NR-HOCl-TFMU** is selective for HOCl in vitro. (a) Reaction of **NR-HOCl-TFMU** with HOCl leads to hydrolysis of the phosphinate ester bond and formation of two fluorescent dyes, **NR**_**666**_ **-MSA** and **TFMU**. (b) Formation of **NR**_**666**_ **-MSA** (Ex: 666 nm, Em: 698 nm) and **TFMU** (Ex: 387 nm, Em: 510 nm) monitored by fluorescence after addition of 3 equiv. of HOCl to **NR-HOCl-TFMU** (5 μM) in PBS (pH = 7.4 with 1% DMF). (c) Images corresponding to **NR-HOCl-TFMU** (50 μM) in PBS (pH = 7.4 with 1% DMF) before (left) and after (right) mixing with 3 equiv. of HOCl. From left to right: white-light image, fluorescence image under a UV lamp (254 nm), and a NIR image with a 680 nm filter. (d) **NR**_**666**_ **-MSA** (Em: 698 nm) or **TFMU** (Em: 510 nm) formation as a function of HOCl concentration, monitored by fluorescence. Fluorescence from solutions containing **NR-HOCl-TFMU** (10 μM) in PBS (pH = 7.4 with 1% DMF) in the presence of the indicated ROS or RNS at the indicated time, fluorescence intensity for **NR**_**666**_ **-MSA** (698 nm, e) and **TFMU** (510 nm, f) is shown. Comparison of fluorescence from **NR-HOCl-TFMU** solutions (10 μM) in response to HOCl, H_2_O_2_, MPO, or MPO-derived HOCl in PBS (pH = 7.4 with 1% DMF), fluorescence intensity for **NR**_**666**_ **-MSA** (698 nm, **g**) and **TFMU** (510 nm, h) is shown after incubating for 1 h. The stability of **NR-HOCl-TFMU** (10 μM) incubated at 37 °C with or without HOCl (4 equiv), H_2_O_2_ (20 equiv), or MPO (1 μg/mL) in PBS (pH = 7.4 with 1% DMF) at the indicated time points. Fluorescence intensity of **NR**_**666**_ **-MSA** (Ex: 640 nm, Em: 698 nm, i) and **TFMU** (Ex: 387 nm, Em: 510 nm, j) was normalized to 0 h. Error bars represent the SD of three technical replicates.

To verify the selective generation of **NR**_**666**_ **-MSA** and **TFMU** upon reaction of **NR-HOCl-TFMU** with HOCl, we treated **NR-HOCl-TFMU** with HOCl, as well as a panel of ROS and RNS, including hydrogen peroxide (H_2_O_2_), *tert*-butyl hydroperoxide (TBHP), superoxide (O_2_^–^), hydroxide radical (·OH), tert-butoxide radical (·OtBu), nitric oxide (NO), and peroxynitrite (ONOO^–^) at a 5-fold excess compared to HOCl (Figure 4e,f). Gratifyingly, we observed no evidence of the formation of either **NR**_**666**_ **-MSA** or **TFMU** in the absence of HOCl for up to 2 h. To further validate the ability to detect enzymatically produced HOCl, we assayed the formation of **NR**_**666**_ **-MSA** and **TFMU** from **NR-HOCl-TFMU** in the presence of MPO with and without hydrogen peroxide (Figure 4g,h). These data demonstrate that the enzymatic activity of MPO, the generation of HOCl, is required for the production of **NR**_**666**_ **-MSA** and **TFMU** from **NR-HOCl-TFMU**. Time-dependent assays revealed that the generation of fluorescence signal was slower from MPO compared to the direct addition of HOCl to **NR-HOCl-TFMU**, potentially due to the time needed for MPO to enzymatically produce HOCl under these conditions (Figure S18).

Having directly verified the ability to release **TFMU** from **NR-HOCl-TFMU** in an HOCl-gated manner and the selectivity of this reaction, we next investigated the stability of **NR-HOCl-TFMU** over longer time periods. Incubation of **NR-HOCl-TFMU** in PBS over 72 h revealed that the phosphinate ester and spirocyclic thioether in this compound were highly stable. Indeed, the evolution of **NR**_**666**_ **-MSA** and **TFMU** fluorescence was selectively observed in samples treated with HOCl and not in the presence of hydrogen peroxide or MPO separately (Figure 4i,j). Taken together, these results clearly demonstrate the ability to gate cargo delivery from phosphinate ester dyes in vitro. Moreover, these data demonstrate that the enzymatic product of MPO, HOCl, is required for gating to occur. Lastly, these results demonstrate the potential stability of phosphinate esters prior to the reaction with a target analyte, providing motivation for the investigation of these reagents as selective drug delivery platforms.

### HOCl-Inducible Cargo Delivery from NR-HOCl-TFMU in Macrophages

RAW 264.7 cells are widely used in immunological research as a model to study macrophage function, including their response to various stimuli,^51^ cytokine production,^52^ and phagocytosis.^53^ For our purposes, this cell line provides an inducible system for production of HOCl since under normal cell culture conditions, these cells express almost no MPO.^54^ However, stimulation with lipopolysaccharide (LPS, a component of the outer membrane of Gram-negative bacteria)^51^ and phorbol 12-myristate 13-acetate (PMA, which stimulates H_2_O_2_ production)^55^ leads to the expression of MPO and subsequent production of HOCl in this cell line. Accordingly, we sought to investigate the ability to selectively deliver cargo to stimulate RAW 264.7 cells using **NR-HOCl-TFMU.**

As expected, cellular autofluorescence was observed in the **TFMU** channel (Ex: 405 nm) for untreated RAW 264.7 cells in the absence of **NR-HOCl-TFMU** (Figure 5a). Subsequent addition of **NR-HOCl-TFMU** to untreated cells resulted in no observable change in fluorescence in the **NR**_**666**_ **-MSA** or **TFMU** channels without washing (Figure 5a), indicating that **NR-HOCl-TFMU** is stable in cell culture media. However, the addition of **NR-HOCl-TFMU** to RAW 264.7 cells costimulated with LPS and PMA resulted in a clearly observable increase in both **NR**_**666**_ **-MSA** and **TFMU** fluorescence within cells (Figure 5a). Quantification of the imaging data demonstrated a 9.8-fold turn on in the **NR**_**666**_ **-MSA** channel and a 1.9-fold turn on in the **TFMU** channel (Figure 5b,c). Since previously reported fluorescence turn-on probes for HOCl have demonstrated the ability to detect HOCl production upon treatment of cells with PMA or LPS separately,^56,57^ we also performed single treatment experiments with each of these agents. Upon treatment with PMA alone, a weak fluorescence enhancement was observed in both channels (Figure 5), potentially due to a PMA-induced increase in the intracellular levels of H_2_O_2_,^55^ a known MPO substrate.^33^ In contrast, LPS stimulation, which is known to increase MPO expression,^58^ led to an intermediate fluorescence increase in the **NR**_**666**_ **-MSA** (5.4-fold) and **TFMU** (1.6-fold) channels (Figure 5). These results are consistent with previously reported fluorescence turn-on sensors for HOCl.^56,57^ While merged images demonstrated that both **NR**_**666**_ **-MSA** and **TFMU** fluorescence were intracellular, the signals from each of these species did not completely overlay. This could be due to the differences in the intrinsic subcellular location of each dye, which can diffuse within cells after the reaction of **NR-HOCl-TFMU** with HOCl. Together, these confocal imaging experiments provide the first direct evidence of the gated release of cargo from **NR** dyes within living cells.

**Figure 5:**
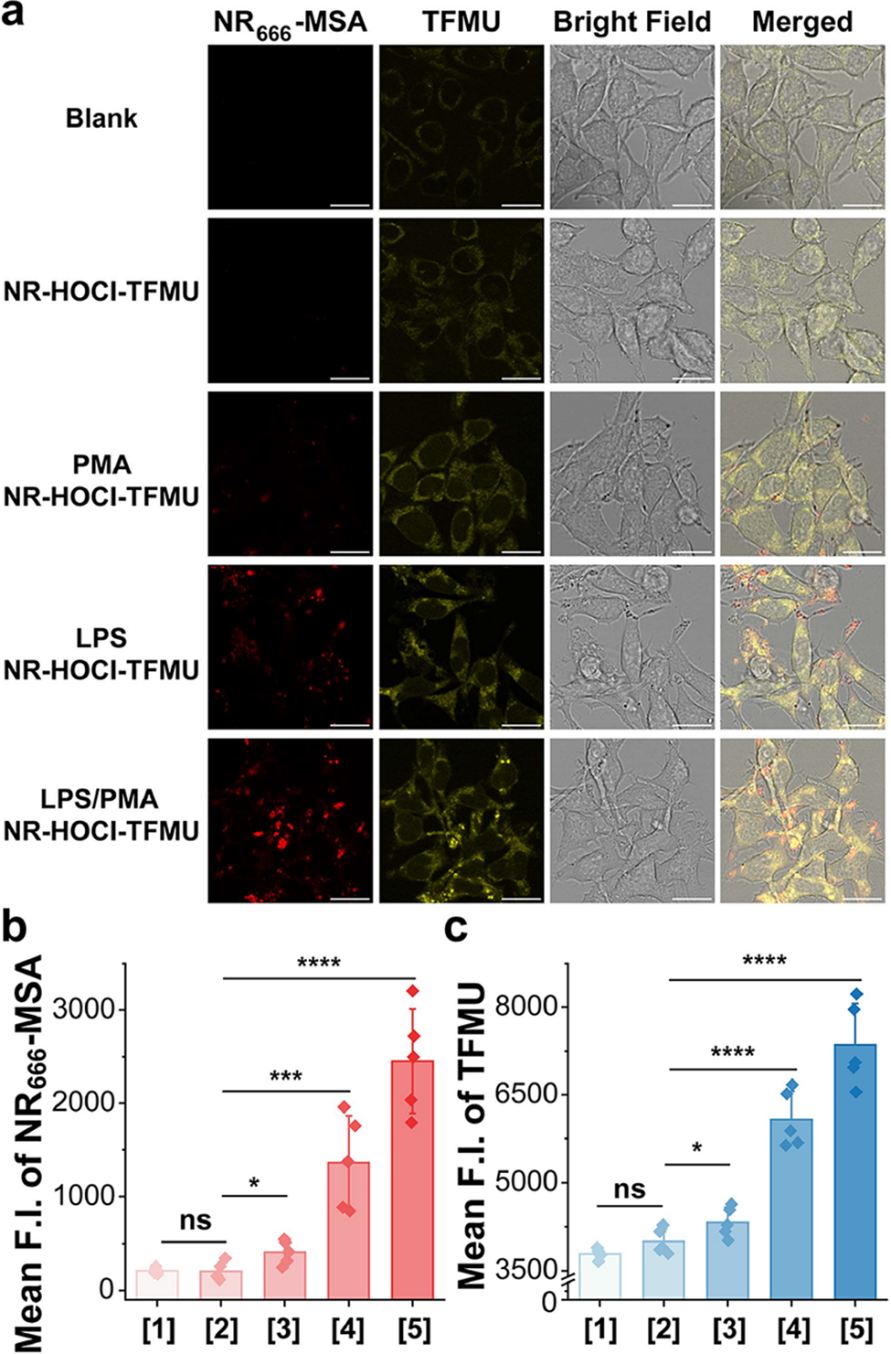
HOCl-inducible cargo delivery from **NR-HOCl-TFMU** in macrophages. (a) No-wash confocal fluorescence imaging of living RAW 264.7 cells. Blank: cells alone, **NR-HOCl-TFMU**: incubation with **NR-HOCl-TFMU** (10 μM) for 30 min, and PMA or LPS: stimulation with the relevant compound (1 μg mL^–1^) for 4 h, followed by the addition of 10 μM **NR-HOCl-TFMU** for 30 min. Scale bar: 15 μm. The mean pixel intensity of the **NR**_**666**_ **-MSA** (b) or **TFMU** (c) channel, numbering refers to samples from panel (a) in increasing order from top to bottom. Error bars represent the SD of the average total fluorescence of images from *n* = 5 biological replicates. Statistical significance was determined using a two-tailed unpaired *t-*test (ns, *p* > 0.05; *p* ≤ 0.05 (*); *p* ≤ 0.001 (***); *p* ≤ 0.0001 (****)).

### HOCl-Gated Cargo Delivery from NR-HOCl-TFMU in AML Cells

To investigate the ability of **NR-HOCl-TFMU** to deliver its cargo in response to disease-relevant levels of HOCl in AML, we chose the well-characterized HL-60 AML cell line, which has been reported to produce HOCl at steady-state concentrations of 1.03 μM^29^ and displays relatively high levels of HOCl relative to established AML cell lines (Figure 2). As a control cell line, we chose a chronic myeloid leukemia (CML) cell line known as K-562, which displays relatively low levels of MPO expression (Figure S5) and HOCl production (Figure S4). Compared with RAW 264.7 cells, confocal imaging of HL-60 or K-562 cells alone revealed cellular autofluorescence in the **TFMU** channel (Figure 6a). However, the addition of **NR-HOCl-TFMU** to HL-60 cells resulted in a 5.5- and 1.5-fold increase in cellular fluorescence in the **NR**_**666**_ **-MSA** and **TFMU** channels without washing, respectively (Figure 6a–c). Gratifyingly, no visible increase in **NR**_**666**_ **-MSA** or **TFMU** fluorescence was observed in K-562 cells incubated with **NR-HOCl-TFMU** (Figure 6a,d,e). Furthermore, incubation of **NR-HOCl-TFMU** with HL-60 cells treated with 4-aminobenzoyl hydrazide (4-ABAH), a known MPO inhibitor,^59^ or N-acetylcysteine (NAC), a ROS scavenger,^60^ reduced the fluorescence signal to background levels (Figure 6a–c). As observed for RAW 264.7 cells, merged images demonstrated that both **NR**_**666**_ **-MSA** and **TFMU** fluorescence were intracellular; however, signals from each of these species did not completely overlay (Figure 6a), potentially due to differences in the intrinsic subcellular location of each dye. These confocal imaging experiments provide the first direct evidence for the HOCl-mediated delivery of small molecules from **NR** dyes in response to endogenous HOCl produced in AML cells. Furthermore, these results demonstrate the selectivity of this gating approach for HOCl in the cellular context.

**Figure 6:**
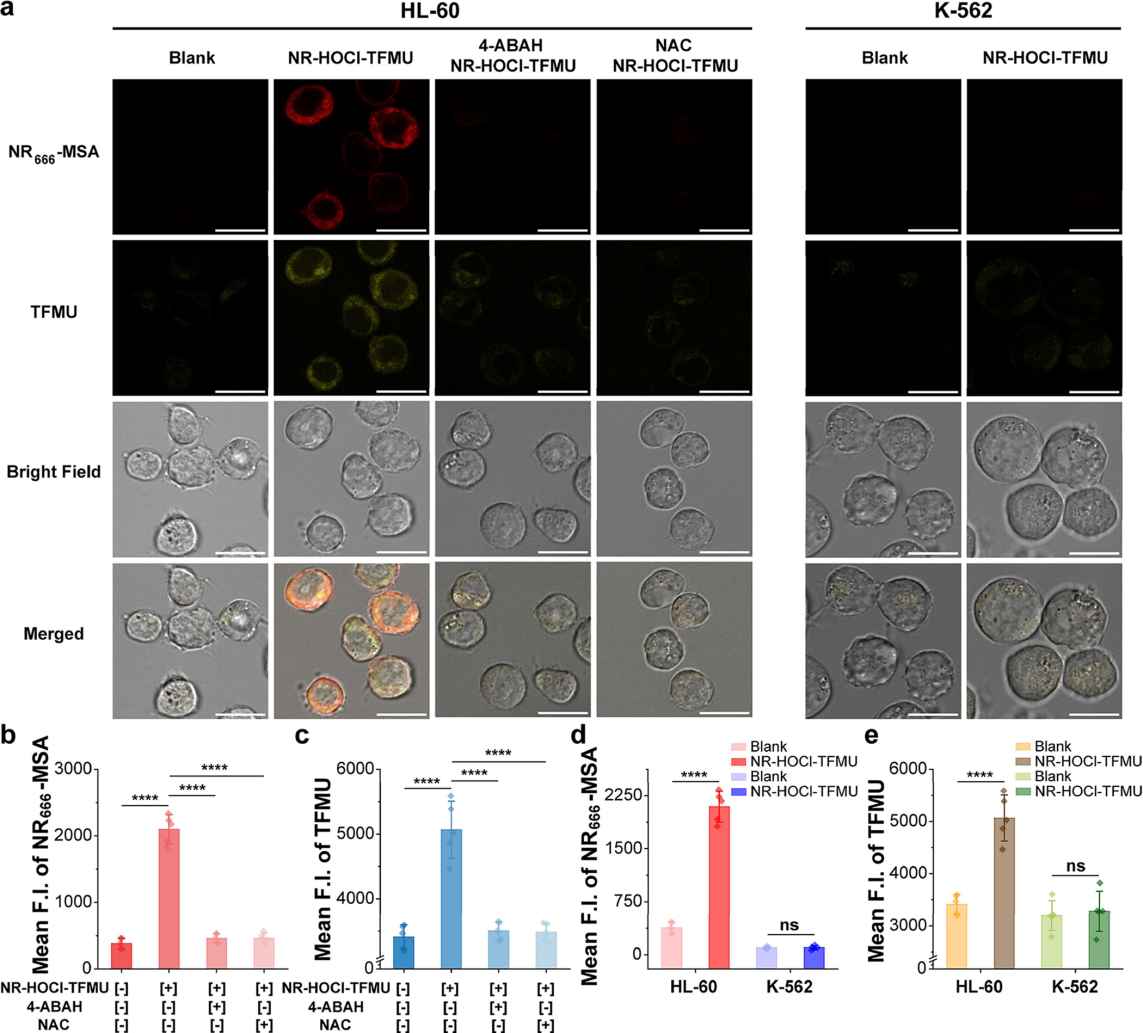
HOCl-gated delivery of **TFMU** from **NR-HOCl-TFMU** in AML cells. (a) No-wash confocal fluorescence imaging of living HL-60 and K-562 cells. (I) Blank, (II) incubated with 10 μM **NR-HOCl-TFMU** for 30 min, (III) treated with 200 μM 4-ABAH for 2 h, followed by incubation with 10 μM **NR-HOCl-TFMU** for 30 min, (IV) treated with 1 mM NAC for 2 h, followed by incubation with 10 μM **NR-HOCl-TFMU** for 30 min. Scale bar: 15 μm. The mean pixel intensity of the **NR**_**666**_ **-MSA** (b) or **TFMU** (c) channel for HL-60 cells for the indicated samples across 5 biological replicates. The mean pixel intensity of the **NR**_**666**_ **-MSA** (d) or **TFMU** (e) channel for HL-60 and K-562 cells for the indicated samples across 5 biological replicates. Error bars = SD for 5 biological replicates. Statistical significance was determined using a two-tailed *t-*test (ns indicates a *p*-value of >0.05, and **** indicates a *p*-value of ≤0.0001).

### HOCl-Gated Cargo Delivery from NR-HOCl-TFMU in a Localized Animal Model of AML

To further evaluate the in vivo feasibility of HOCl-triggered cargo release and delivery from **NR-HOCl-TFMU**, a localized tumor model was established by inoculating HL-60-Luc2 cells (high MPO and HOCl) or K-562-Luc2 (HOCl-negative control) in a basement membrane matrix into the right flank of NOD-SCID-gamma (NSG) mice (Figures S19 and S20). Prior to in vivo fluorescence imaging, a pilot test was conducted in vitro to determine the optimal excitation and emission filter combinations for detecting **NR**_**666**_ **-MSA** and **TFMU** (Figure S21). Fluorescence images were acquired before and after intratumoral injection of **NR-HOCl-TFMU** (Figures 7a and S22).^61^ Before injection, both HL-60-Luc2 and K-562-Luc2 tumors exhibited similar baseline fluorescence (Figure 7b). Following **NR-HOCl-TFMU** administration, a 1.9-fold increase in the **NR**_**666**_ **-MSA** fluorescence was observed in HL-60-Luc2 tumors (Figure 7b). In contrast, K-562-Luc2 tumors showed no significant increase in the **NR**_**666**_ **-MSA** fluorescence. Due to the blue-shifted excitation and emission of **TFMU**, its fluorescence could not be directly detected in vivo. Therefore, following NIR imaging, tumors were excised, flash frozen, and sectioned into 400 μm slices for ex vivo confocal fluorescence imaging (Figure 7c). Consistent with in vivo imaging results, NIR fluorescence from **NR**_**666**_ **-MSA** was observed in HL-60-Luc2 tumors but not in K-562-Luc2 controls. Importantly, **TFMU** fluorescence was also detected in HL-60-Luc2 tumors, confirming the HOCl-triggered cargo release and NIR signal generation from **NR-HOCl-TFMU**. Z-stack analysis of images from different depths in these tissues revealed that the intensity profiles of both **NR**_**666**_ **-MSA** and **TFMU** fluorescence colocalized (Figure 7d), further demonstrating the ability to use **NR**_**666**_ **-MSA** fluorescence to define the location of cargo delivery.

**Figure 7:**
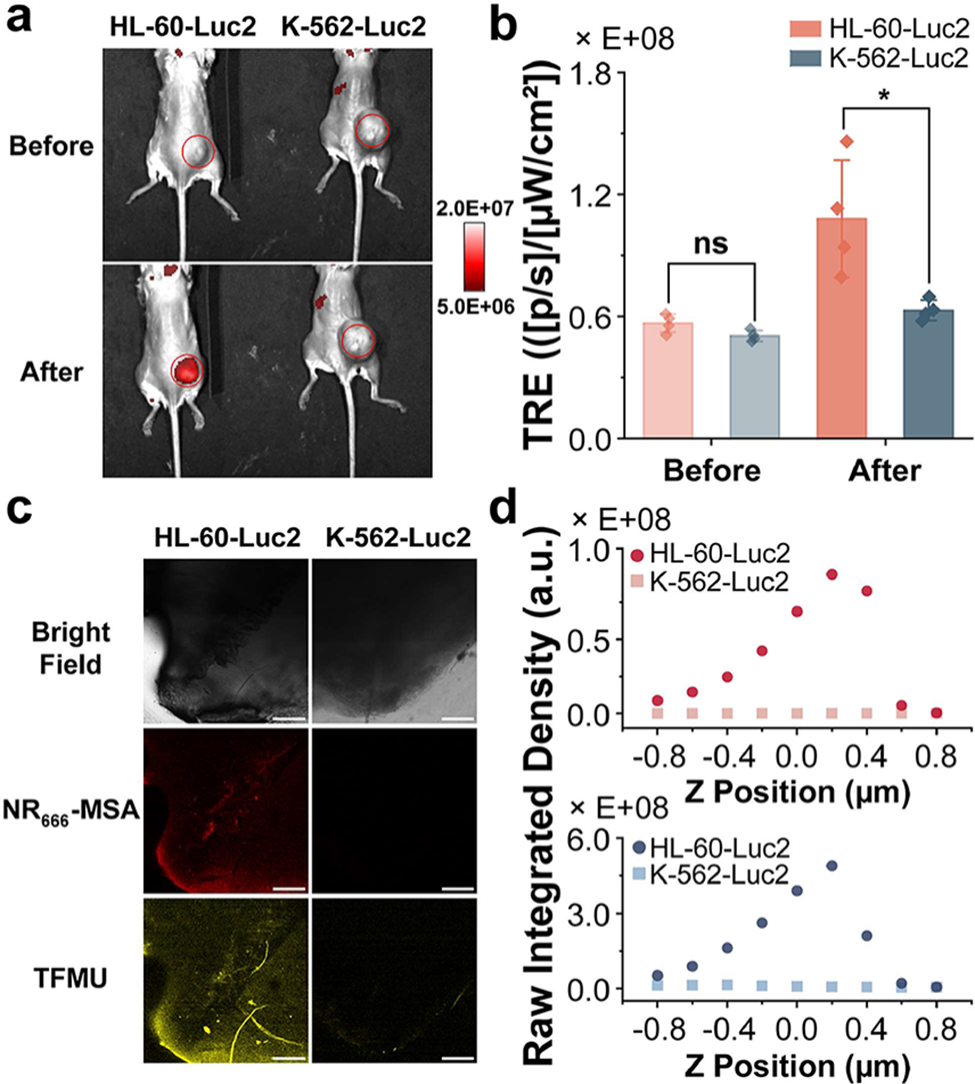
In vivo and ex vivo evaluation of HOCl-gated fluorescence activation and cargo release from **NR-HOCl-TFMU** using a localized animal model. (a) Representative in vivo NIR fluorescence images of HL-60-Luc2 and K-562-Luc2 tumor-bearing mice before and immediately after intratumoral injection of **NR-HOCl-TFMU** (100 μM in saline with 10% DMF). Images were acquired using 675 nm excitation and 760 nm emission filters. Red circles indicate tumor locations, and the color scale represents total radiant efficiency (TRE) in ([p/s]/[μW/cm^2^]). (b) Quantification of NIR fluorescence intensity in respective tumors before and after **NR-HOCl-TFMU** administration. Data represent mean ± SD from 4 biological replicates. Statistical analysis was performed using a two-tailed *t-*test (ns indicates a *p*-value of >0.05, and * indicates a *p*-value of ≤ 0.05). (c) Confocal fluorescence imaging of 400 μm thick slices from HL-60-Luc2 and K-562-Luc2 tumors following **NR-HOCl-TFMU** injection. Scale bar: 200 μm. (d) Z-stack quantification of confocal images from different depths (−0.8 to +0.8 μm relative to the focal plane) in the tissues from panel (c). Raw integrated density values are shown for **NR**_**666**_ **-MSA** (above) and **TFMU** (below) channels.

### Gated Toxicity of NR-HOCl-TFMU in AML Cells

Lastly, we evaluated the cytotoxicity of **NR-HOCl-TFMU** in the HL-60 and K-562 cell lines (Figure 8). For the concentration of **NR-HOCl-TFMU** used for imaging (10 μM), no significant toxicity was observed in either cell line up to 24 h. Interestingly, we observed selective toxicity of **NR-HOCl-TFMU** in HL-60 compared to K-562 cells at higher concentrations and longer incubation times, with the viability of HL-60 cells decreasing by 50% after 24 h of incubation with 50 μM **NR-HOCl-TFMU** (Figure 8). This result implies that the toxicity of **NR-HOCl-TFMU** is gated by HOCl as no significant toxicity was observed in K-562 cells at equal concentrations and incubation times. To identify whether **NR**_**666**_ **-MSA** or **TFMU** was responsible for this observed effect, we incubated HL-60 and K-562 cells with **TFMU** alone (Figure S23). Under these conditions, we observed no cytotoxicity for **TFMU** in either cell line using the same concentrations and incubation times as those for **NR-HOCl-TFMU**. However, it should be noted that the cellular permeability of free **TFMU** is likely relatively low due to the phenol p*K*_a_ of this dye. Since **NR**_**666**_ **-MSA** is not cell permeable, we chose to generate **NR**_**666**_ **-MSA** in HOCl-producing HL-60 cells by incubation with **NR-HOCl** (Figure 2), which we have previously shown is highly selective for HOCl and can cross the cell membrane and form **NR**_**666**_ **-MSA** upon reaction with endogenous HOCl (Figure 2).^7,11^ Strikingly, the toxicity profile of **NR-HOCl** matched that of **NR-HOCl-TFMU**, with *a* > 50% reduction in the viability of HL-60 cells after 24 h of incubation with 50 μM **NR-HOCl** (Figure S24). As with **NR-HOCl-TFMU**, no cellular toxicity was observed for 50 μM **NR-HOCl** in the non-HOCl-producing K-562 cell line under identical conditions.

**Figure 8:**
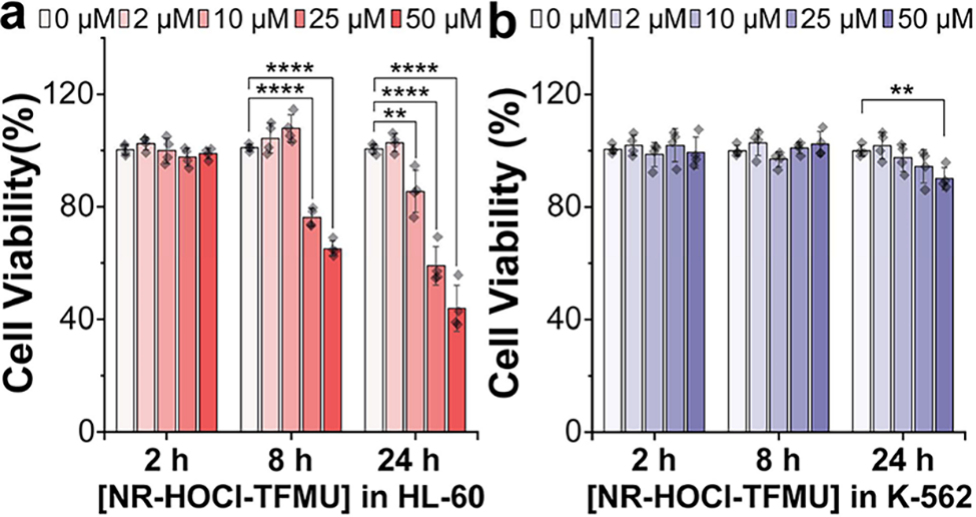
HOCl-gated toxicity of **NR-HOCl-TFMU** in HL-60 cells (a) compared to K-562 (b) cells. Cells were incubated with the indicated concentration of **NR-HOCl-TFMU** (1% DMF) in the corresponding cell culture media without phenol red. Cell viability was assessed at the indicated time point using the commercially available CCK-8 assay. Data represent four biological replicates. Error bars indicate mean ± SD. Statistically significant differences were determined using a two-tailed *t-*test and are indicated as ** for *p* ≤ 0.01, *** for *p* ≤ 0.01, and **** for *p* ≤ 0.0001.

Given that cytotoxicity is observed at relatively high concentrations (>25 μM) and long exposure times (>8 h) for **NR-HOCl-TFMU**, we considered the possibility that selective **NR-HOCl-TFMU**-mediated cell death in HL-60 cells might result from depletion of HOCl or differences in compound uptake between HL-60 and K-562 cells. To investigate both of these issues, we generated an MPO-knockout HL-60-Luc2 cell line using CRISPR/Cas9 gene editing (Figure S25) and confirmed the loss of MPO expression by Western blotting compared to the wild-type cells (Figure S26). Importantly, the ability to obtain an MPO-knockout HL-60-Luc2 cell line demonstrates that MPO and its enzymatic product (HOCl) are not essential for cell survival. We then evaluated the cytotoxicity of **TFMU**, **NR-HOCl**, and **NR-HOCl-TFMU** in both wild-type and MPO-knockout HL-60-Luc2 cells across a range of concentrations and incubation times (Figures S27–S29). Consistent with results in HL-60 and K-562 cells (Figures 8, S23 and S24), **TFMU** did not induce toxicity in either wild-type or MPO-knockout HL-60-Luc2 cells. In contrast, both **NR-HOCl** and **NR-HOCl-TFMU** caused >50% cell death in wild-type HL-60-Luc2 cells at 50 μM compound after 24 h, while the MPO-knockout cells remained virtually unaffected (equivalent to K-562 cells, Figures 8 and S24) under the same conditions. Considering that the only difference between wild-type and MPO-knockout HL-60-Luc2 cells is the expression of MPO and subsequent production of HOCl, and the selectivity of **NR-HOCl-TFMU** for HOCl (Figures 4–7), we attribute the toxicity of **NR-HOCl-TFMU** in HL-60 cells (Figure 8) to the HOCl-gated formation of **NR**_**666**_ **-MSA**. These unexpected results clearly demonstrate the ability to gate compound delivery, as well as cytotoxicity in HOCl-producing cells. Although the current potency of **NR**_**666**_ **-MSA** in HOCl-producing cells is low, attachment of a potent cytotoxic cargo to the phosphinate ester of **NR-HOCl** is expected to dramatically improve efficacy for HOCl-producing cells with the approach described above.

## Conclusions

Herein, we provide the first direct evidence for the gated delivery of small-molecule cargo from phosphinate ester dyes. We define the optimal p*K*_a_ for phenol-containing cargo as ≥7.2 and leverage a mechanistic understanding of phosphinate ester hydrolysis to construct **NR-HOCl-TFMU**, which is capable of producing both NIR (**NR**_**666**_ **-MSA**) and blue (**TFMU**) fluorescence upon reaction with HOCl. Importantly, the fluorescence of the NIR reporter, **NR**_**666**_ **-MSA**, is proportional to the amount of cargo (**TFMU**) released (Figure 4d), and **NR-HOCl-TFMU** is stable for days prior to reaction with HOCl (Figure 4i,j). We further demonstrate that gated small-molecule delivery in this system is highly selective for HOCl in vitro, in living cells, and in a localized mouse model of AML (Figures 4e,f and 5–7). Given that continuous production of HOCl is restricted to AML cells,^27–29^ this intracellular analyte represents a potentially underexplored trigger for the selective release of cytotoxic agents in AML cells. Indeed, we unexpectedly observed selective toxicity for **NR-HOCl-TFMU** in HOCl-producing HL-60 cells (Figure 8), providing a proof-of-principle for this application. Ongoing work in our lab is focused on replacing the cargo in **NR-HOCl-TFMU** with potent cytotoxic compounds and evaluating the toxicity of these reagents in HOCl-positive AML cells, as well as normal cells. Since HOCl is produced in activated granulocytes during the normal immune response to bacterial infection,^29,31–33^ we expect to observe off-target activity in activated granulocytes. However, given that most AML patients present with neutropenia at the time of diagnosis^37,38^ and the relatively severe off-target effects of cytotoxic chemotherapy,^26^ we do not envision off-target activity in activated granulocytes to be a significant impediment to further development of this approach. Preclinical testing in animal models will uncover any potential off-target toxicity due to xenobiotic metabolism of reagents similar to that of **NR-HOCl-TFMU**. In the long term, we envision the potential selective delivery of cytotoxic agents to AML cells using the approach. While this proof-of-principle study demonstrates the ability to gate the hydrolysis of phosphinate ester-containing dyes in living cells and represents an important step toward this goal, several limitations will need to be overcome to achieve delivery of therapeutic agents using this approach. These limitations include stability of phosphinate ester-containing dye conjugates in the bloodstream and their ability to reach target cells, off-target phosphinate ester hydrolysis in normal cells and tissues, and on-target delivery of cytotoxins in normal cells producing HOCl (e.g., activated neutrophils). The current proof-of-principle work indicates that gated hydrolysis of the phosphinate ester-containing dyes can be achieved in cells (Figures 5 and 6) and in the tumor microenvironment (Figure 7). Since this strategy integrates the reporter, reactive linker, and targeting group into a single unit (a phosphinate ester dye), the resulting compounds may display more desirable pharmacokinetic properties compared to traditional theranostics while preserving the ability to image cargo delivery to target cells. Additionally, the ability to image drug delivery will aid in the identification and suppression of off-target effects in normal cells and tissues. Lastly, since MPO enzymatic activity in phagocytic white blood cells (e.g., neutrophils and macrophages) is the major source of HOCl in normal human physiology^62–65^ and many AML patients are neutropenic at diagnosis,^37–39^ we do not anticipate substantial issues associated with on-target delivery of cytotoxins to normal cells or activated neutrophils. Building on this proof-of-principle work, current efforts in our laboratory are focused on continuing to address these potential limitations in the context of phosphinate ester-containing dyes bearing cytotoxic cargos.

Beyond AML, these results indicate that a broad array of activity-based sensing approaches for small-molecule analytes or enzymatic activities could be used to gate phosphinate ester hydrolysis.^14,15,17,66^ Ongoing work in our lab is also pursuing a variety of gated reagents for different disease states, the ability to utilize attachments other than alcohols for cargos, and the influence of structural modifications to the **NR** dye core on the rate of cargo delivery. Thus, we believe that the novel theranostic approach described herein will find broad application in a variety of disease and biomedical research contexts.

## Supplementary Material



## Notes

Experimental details, including synthetic procedures, structural characterization, and supporting figures (PDF)

## ◆

Department of Chemistry, University of California, Berkeley, California 94720, United States

## ¶

Department of Biomedical Engineering, Oregon Health and Science University, Portland, Oregon 97201, United States.
