# Supplementary material for: Hypochlorous Acid-Gated Hydrolysis of a Phosphinate Ester Dye in Living Cells
Source: J Am Chem Soc. 2025 Oct 22;147(44):40590–602. doi: 10.1021/jacs.5c12615 (PMC12588358; doi:10.1021/jacs.5c12615)
Supplement: Supplementary file 1 [file ja5c12615_si_001.pdf]

Supporting Information

Hypochlorous Acid-Gated Hydrolysis of a Phosphinate Ester Dye in Living Cells

Yuan Fang,[‡a] Xinqi Zhou,[‡b,c] Julia L. McAfee,[a] Benjamin M. Faulkner,[a] Lauren Lesiak,[b,c] Yuchen He,[a] Frederik Brøndsted,[a,d] Hao Fan,[e,f] Eric D. Donarski,[a] Xiaoyan Hu,[g,h] B. Jill Venton,[a] Steven Grant,[g,h] Francine E. Garrett-Bakelman,[e,f,i] and Cliff I. Stains[a,i,j]*

[a]Department of Chemistry, University of Virginia, Charlottesville, VA 22904, USA

[b]Department of Chemistry, University of Nebraska-Lincoln, Lincoln, NE 68588, USA

[c]Current Address: Department of Chemistry, University of California, Berkeley, CA 94720, USA

[d]Current Address: Department of Biomedical Engineering, Oregon Health & Science University, Portland, OR 97201, USA

[e]Department of Medicine, University of Virginia, Charlottesville, VA 22904, USA

[f]Department of Biochemistry and Molecular Genetics, University of Virginia, Charlottesville, VA 22904, USA

[g]Division of Hematology/Oncology, Department of Medicine, Virginia Commonwealth University, 23298, Richmond, VA, USA.

[h]Massey Cancer Center, Virginia Commonwealth University, 23298, Richmond, VA, USA.

[i]University of Virginia Cancer Center, University of Virginia, Charlottesville, VA 22908, USA

[j]Virginia Drug Discovery Consortium, Blacksburg, VA 24061, USA

‡These authors contributed equally to this work.

*E-mail: cstains@virginia.edu

Table of Contents

General Experimental Details....................4
Reagents and Instrumentation....................4
Stock Preparations of **NR** Dye Conjugates....................5
Determination of HOCl and H_2O_2 Stock Concentrations....................6
Determination of Apparent Hydrolysis Rates....................6
pK_a Determination and pH-Dependent Activation....................6
Selectivity Assay....................6
Flow Cytometry Analysis....................7
Western Blotting Analysis....................7

Cell Imaging 7
Localized AML Tumor Mice Imaging 8
Generation of HL-60-Luc2 MPO-knockout cells by CRISPR/Cas9 9
Cell Toxicity Assay 9
Synthesis Procedures 10
Synthesis of **NR_{666}-HOCl** 10
General Synthetic Conditions for CDI Coupling 10
Synthesis of **NR-HOCl-4MU** 10
Synthesis of **NR-HOCl-TFMU** 11
Figure S1 12
Figure S2 13
Figure S3 14
Figure S4 17
Figure S5 18
Figure S6 19
Figure S7 20
Figure S8 21
Figure S9 22
Figure S10 23
Figure S11 24
Figure S12 25
Figure S13 26
Figure S14 27
Figure S15 28
Figure S16 29
Figure S17 31
Figure S18 32
Figure S19 33
Figure S20 34
Figure S21 35
Figure S22 36
Figure S23 37
Figure S24 38

Figure S25 39
Figure S26 40
Figure S27 41
Figure S28 42
Figure S29 43
Copies of ^{1}H, ^{13}C, ^{19}F and ^{31}P NMR Spectrum 44
References 49

General Experimental Details

Reagents and Instrumentation

Unless otherwise noted, all reagents and solvents were used as commercially supplied. Reaction progress was monitored by using thin layer chromatography (TLC, silica gel, F254, 250 μm) and products were purified by flash chromatography using Merck silica gel 60 (230 - 400 mesh). High-performance liquid chromatography (HPLC) purification was conducted using a Waters 1525 Binary HPLC pump with a 2489 UV/Vis detector. Large scale purification was done with a semi-prep column (YMC-Pack ODS-A, 5 μm, 20 × 250 mm) using a gradient of 5 - 95% acetonitrile containing 0.1% trifluoroacetic acid (TFA) over 40 mins in water containing 0.1% TFA. Analytical HPLC was conducted with an analytical column (YMC-Pack ODS-A, 5 μm, 4.6 x 250 mm) using a gradient of 5 - 95% acetonitrile containing 0.1% trifluoroacetic acid (TFA) over 30 mins in water containing 0.1% TFA. Final compounds were lyophilized in a Labconco™ FreeZone™ (4.5 L, -84 °C) after semi-prep HPLC. Low-resolution ESI mass spectrometry was performed with an Advion CMS-S01 ESI mass spectrometer, and high-resolution mass spectrometry was obtained using an Agilent 6545 Q-TOF paired with an Agilent 1260 Infinity II Prime liquid chromatography (LC) system. Mass data are reported in units of m/z for $[M+H]^+$. ^{1}H-NMR, ^{13}C-NMR, ^{31}P-NMR, and ^{19}F-NMR were performed in DMSO-d_6 at room temperature, spectra were recorded on Varian VNMRS 600 MHz and data were processed with MestReNova software. For ^{19}F-NMR spectra, trifluoroacetic acid (TFA) was used as an internal standard with a chemical shift (δ) of -76.55, and ^{31}P-NMR spectra are reported using H_3PO_4 (δ 0.00) as an external standard. UV-Vis-NIR absorbance spectra were recorded on a Jasco V-780. UV-Vis band width was set to 1 nm, and NIR band width was set to 2 nm. Response time for both UV/Vis and NIR was 0.96 s. Scanning interval was set as 1 nm and scan speed was 400 nm/min (continuous mode). Fluorescence spectra were acquired on a Horiba Fluorolog-QM. This fluorimeter was equipped with a 75 W Xenon Arc lamp with PowerArc™ lamp housing (OB-75X) and a photomultiplier tube (920 PMT) detector. For collecting spectra, assays were conducted in 3.5 mL standard rectangular quartz cuvettes or sub-micro spectrophotometer quartz cells (Z = 15 mm) with 1 cm optical path length. Determination of pK_as, hydrolysis rates, selectivity and stability assays, and cell toxicity were performed on a Synergy H1 Hybrid Multi-Mode Reader (BioTek Instruments) or Tecan Spark® Multimode Microplate Reader with flat bottom clear 96-well assay plates (Corning® CLS3370, Thermo Fisher Scientific 167574) and flat bottom half-area black 96-well microplates (Corning® CLS3694), respectively. Visual photographs were taken using a Canon EOS 5D Mark III. NIR fluorescence photographs were taken using a homemade full-spectrum DSLR camera (Canon Rebel XSi) with a 720 nm NIR filter (Hoya 67 mm RM72 Infrared Filter). Image acquisition for Western blotting was captured with a ChemiDoc XRS + Gel imaging system (Bio-Rad). Confocal fluorescence imaging was performed using a Leica STELLARIS 8 Laser scanning confocal/spectral imaging microscope equipped with a tunable white light laser. The excitation laser line was set at 650 nm with an emission PMT filter of 680 - 780 nm for the **NR_{666}-MSA** channel. A 405 nm diode light source with an emission PMT filter of 450 - 600 nm was used for the 7-Hydroxy-4-(trifluoromethyl)coumarin (**TFMU**) channel. Images were acquired using LAS-AF software and data analysis was performed in Fiji software (ImageJ). Flow cytometry data were acquired using an Invitrogen™ Attune™ NxT

Flow Cytometer with 96-well U-bottom plates (Greiner Bio-One, Cat. No. 650101). NIR fluorescence was detected using the RL2 channel (excitation at 637 nm, emission filter 720/30 nm). Data was analyzed using FCS Express 7 Research Edition (De Novo Software). Mice imaging was conducted using an IVIS Spectrum *in vivo* Imaging System (PerkinElmer) with 675 nm excitation and 760 nm emission filters. Fluorescence imaging data were analyzed using Aura Imaging Software (Spectral Instruments Imaging). For *ex vivo* imaging, tumor tissue slices of 400 μm thickness were prepared using a Leica VT1000 S vibrating microtome.

Gibco™ PBS (Phosphate Buffered Saline, pH = 7.4, Thermo Fisher Scientific, 10010023)

DMF (Dimethylformamide, Sigma, 227056 (anhydrous) and D4551 (molecular biology))

DMSO (Dimethyl sulfoxide, Sigma, 276855 (anhydrous))

Britton-Robinson buffer (made following a published protocol)[1]

MPO (Myeloperoxidase, Sigma, M6908)

Gibco™ DMEM (Dulbecco's Modified Eagle Medium, Thermo Fisher Scientific, 10569010)

Gibco™ IMDM (Iscove's Modified Dulbecco's Medium, Thermo Fisher Scientific, 31980030 (with phenol red) and 21056023 (no phenol red))

Gibco™ FBS (Fetal Bovine Serum, Thermo Fisher Scientific, 16000044)

Gibco™ Anti-Anti (Antibiotic-Antimycotic, Thermo Fisher Scientific, 15240112)

Gibco™ HBSS (Hanks' Balanced Salt Solution with calcium and magnesium, Thermo Fisher Scientific, 14025092)

LPS (Lipopolysaccharide, Sigma, L4391, made as 5 mg/mL stock in H_2O and stored at -80 °C)

PMA (Phorbol 12-myristate 13-acetate, Sigma, P1585, made as 5 mg/mL stock in ethanol and stored at -80 °C)

4-ABAH (4-Aminobenzoic Acid hydrazide, Cayman Chemical Company, 14845, made as 20 mM stock in anhydrous DMF and stored at -80 °C)

NAC (N-acetyl-L-Cysteine, Cayman Chemical Company, 20261, made as 100 mM stock in anhydrous DMF and stored at -80 °C)

CCK-8 (Cell Counting Kit-8, ApexBio, K1018)

Stock Preparations of NR Dye Conjugates

The lyophilized NR dye conjugates (solid) were dissolved in anhydrous DMF and prepared as 1 mM, 5 mM, and 10 mM stock solutions, aliquoted and stored at -80 °C for later use. The 10 mM DMF stock of **NR-HOCl-TFMU** was stable at -80 °C for over 6 months.

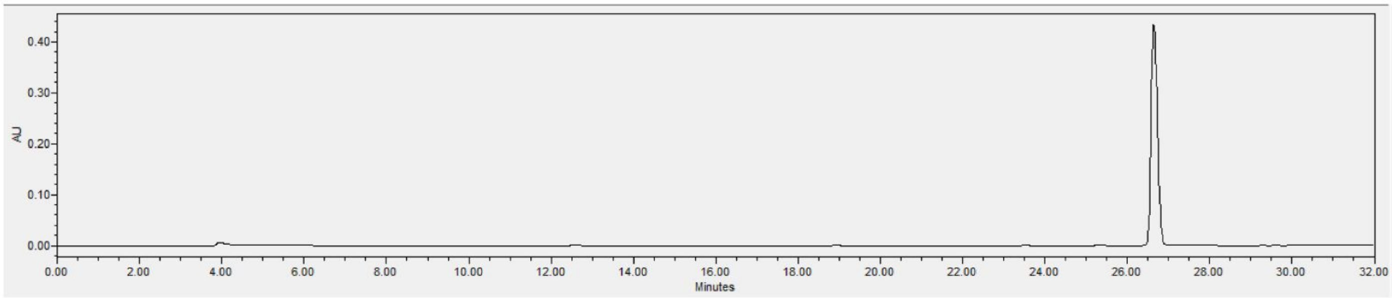

Analytical HPLC purity check of 10 mM **NR-HOCl-TFMU** DMF stock (stored at -80 °C for 6 months at 254 nm).

Determination of HOCl and H_2O_2 Stock Concentrations

HOCl stock solution was freshly prepared before each assay by diluting sodium hypochlorite solution (Sigma, 239305) in PBS, the stock concentration was determined by the absorbance intensity of ^{-}OCl at 292 nm using an extinction coefficient (ε) of 350 $M^{-1}\cdot cm^{-1}$.[2] The stock was then stored on ice, avoiding exposure to light, for further use. H_2O_2 stocks were prepared in the same way as HOCl stocks. H_2O_2 (Sigma, 516813) was diluted in PBS, and the stock concentration was calculated using ε = 43.6 $M^{-1}\cdot cm^{-1}$ at 240 nm.[2]

Determination of Apparent Hydrolysis Rates

Assays (150 µL each) were performed in a half-area black 96-well microplate with 5 µM dye in PBS (pH = 7.4, 1% DMSO). The fluorescent intensity of the corresponding hydrolyzed product was monitored over time and measured in triplicate. The fluorescence increase over time was fit to the equation $y = A_1 \times \exp(-x/t_1) + y_0$, where A_1 = amplitude, t_1 = time constant, and y_0 = offset. The apparent half-life was calculated by $t_{1/2,\ app} = \ln(2) \times t_1$.

p*K*a Determination and pH-Dependent Activation

For pK_a determination, fluorophore solutions (10 µM) were made with Britton-Robinson buffer (1% DMSO) at the pH range from 2-12, absorbance spectra were recorded using clear bottom 96-well plates (200 µL per well). For pH-dependent activation, **NR-HOCl-TFMU** (10 µM) was mixed with different pH Britton-Robinson buffer ranging from 2-10 (1% DMF) with and without adding 4 equiv. HOCl. Fluorescent emission intensity was evaluated at 698 nm (Ex: 640 nm) and 510 nm (Ex: 387 nm) using flat bottom half-area black 96-well microplates (150 µL per well). Data at each pH was measured in triplicate using a plate reader.

Selectivity Assay

All ROS/RNS solutions were prepared according to previous literature procedures.[2-3] The concentration of HOCl was kept at 40 µM and all the other ROS concentrations were kept at 200 µM. **NR-HOCl-TFMU** was prepared at 10 µM in PBS (pH = 7.4 with 1% DMF) containing different concentrations of ROS/RNS. Assays were performed in a half-area black 96-well plates (150 µL each well) and measured in triplicate. Fluorescence emission intensity was monitored at 698 nm for **NR_{666}-MSA** (Ex: 640 nm) and 510 nm for **TFMU** (Ex: 387 nm) at room temperature after 15 min or 120 min incubation time. All the data was normalized by the intensity of the blank at time 15 min.

Flow Cytometry Analysis

All AML cell lines were cultured according to ATCC protocols. Cells were resuspended in PBS and 100 μL of the suspension (1×10^6 cells/mL) was added to each well of a 96-well U-bottom plate. An additional 100 μL of PBS containing either 1% DMF (blank control) or 10 μM **NR-HOCl** with 1% DMF (treatment group) was added to each well. The plate was then incubated at 37 °C in a CO_2 incubator for 1 h. After incubation, each well was thoroughly mixed using a multichannel pipette to ensure uniform suspension. Flow cytometry data were acquired using an Invitrogen™ Attune™ NxT Flow Cytometer at a flow rate of 500 μL/min. NIR fluorescence was detected using the RL2 channel (excitation at 637 nm, emission filter 720/30 nm). Data acquisition was stopped after 10,000 events. Each condition was prepared in triplicate.

Western Blotting Analysis

Cells were grown to 1×10^6 cells/mL. After centrifugation at 4 °C with 100 × g for 10 min, cell culture medium was removed by vacuum. The cell pellets were then lysed by addition of the following lysis buffer: 50 mM Tris-Cl (pH = 7.5 at 25 °C), 150 mM NaCl, 50 mM β-glycerophosphate, 10 mM sodium pyrophosphate, 30 mM NaF, 1% Triton X-100, 2 mM EGTA, 100 μM Na_3VO_4, 1 mM DTT, protease inhibitor cocktail III (10 μL/mL, Calbiochem, 539134), and phosphatase inhibitor cocktail 1 (10 μL/mL, Sigma, P2825). The lysates were placed on ice for 15 min followed by centrifugation at 17,000 g for 10 min at 4 °C. A Bradford assay (Bio-Rad, 5000201) was performed to determine total protein concentrations using BSA as a standard, which were then normalized for downstream experiments. For Western blotting, cell lysates (5 μg total protein) were separated using 12% SDS-PAGE gels and transferred onto nitrocellulose membranes. If Ponceau staining was used, the membrane was incubated in Ponceau S Staining Solution (Cell Signaling Technology, #59803) for 10 minutes at room temperature followed by washing with ddH_2O and 1 × TBST. The membranes were blocked with 5% BSA (Bovine Serum Albumin, Sigma, A1470) in 1 × TBST (Tris-buffered saline with 0.1% Tween® 20) at room temperature for 1 h with shaking. After washing with 1 × TBST three times. The membranes for GAPDH and MPO were separately probed with diluted GAPDH (14C10) Rabbit mAb (HRP Conjugate, 1:1000, Cell Signaling Technology, #3683) or Myeloperoxidase (E1E7I) XP® Rabbit mAb (1:1000, Cell Signaling Technology, #14569T) in 5% BSA, 1 × TBST at 4 °C with gentle shaking overnight. The membranes were washed with 1 × TBST three times and further incubated with diluted Peroxidase (HRP) Anti-Rabbit IgG Goat Secondary Antibody (1:2000, Cell Signaling Technology, #7074S) in 5% BSA, 1 × TBST with gentle agitation for 1 h at room temperature. The membranes were then washed with 1 × TBST three times for 5 min. Detection was achieved by using SuperSignal™ West Dura Extended Duration Chemiluminescent Substrate (Thermo Fisher Scientific, 34076) and images were acquired using a gel imaging system.

Cell Imaging

HeLa (ATCC, CCL-2) cells were grown to 80% confluency in DMEM with 10% FBS and 1 × Anti-Anti in 35 mm dishes with No. 1.5 Poly-D-Lysine coated coverslip (MatTek Corporation, P35GC-1.5-14-C) at 37 °C in a humidified atmosphere with 5% CO_2. The media was removed, and cells were washed with pre-warmed HBSS.

Cells were then incubated with or without the 10 μM **4MU** (1% DMSO) in HBSS for 30 min. After washing with pre-warmed HBSS three times, cells were used directly for imaging in HBSS with temperature control (37 °C).

RAW 264.7 (ATCC, TIB-71) cells were grown using the same culturing condition as HeLa cells. After washing with the pre-warmed HBSS, cells in some dishes were stimulated with LPS (1 μg/mL) or PMA (1 μg/mL) or co-stimulated with LPS and PMA (1 μg/mL each) for 4 h. Then cells in all the dishes were further incubated with or without **NR-HOCl-TFMU** (10 μM, 1% DMF) in HBSS for 30 min. Cells were used directly for imaging at 37 °C without washing.

HL-60 (ATCC, CCL-240) cells were cultured in IMDM with 20% FBS and 1 × Anti-Anti. After centrifugation at 100 × g for 10 min, the cell culture medium was removed by vacuum and the cell pellets were re-suspended with HBSS and the distributed into 12-well glass bottom plates with high performance #1.5 cover glass (Cellvis, P12-1.5H-N). Cells in some wells were treated with 200 μM 4-ABAH or 1 mM NAC for 2 h. Cells in all the wells were further incubated with or without **NR-HOCl-TFMU** (10 μM, 1% DMF) for 30 min and fluorescence imaging was performed at 37 °C without washing.

K-562 (ATCC, CCL-243) cells were cultured in IMDM with 10% FBS and 1 × Anti-Anti. Cells were collected in an identical manner to HL-60 cells and the cells were re-suspended with HBSS and the distributed into 12-well glass bottom plates. The cells were further incubated with or without **NR-HOCl-TFMU** (10 μM, 1% DMF) in HBSS for 30 min and imaged without washing at 37 °C.

<u>Localized AML Tumor Mice Imaging</u>

All animal experiments were performed with the approval of the Institutional Animal Care and Use Committee (IACUC) of Virginia Commonwealth University, following principles outlined by the American Physiological Society on research animal use.

MPO-positive HL-60-Luc2 (ATCC, CCL-240-LUC2) cells were cultured in IMDM + 20% FBS + 1 × Anti-Anti + 8 μg/mL Gibco™ Blasticidin (Thermo Fisher Scientific, A1113903), and MPO-negative K-562-Luc2 (ATCC, CCL-243-LUC2) cells were grown in IMDM + 10% FBS + 1 × Anti-Anti + 8 μg/mL Blasticidin. A localized tumor model was established by subcutaneous inoculation of HL-60-Luc2 or K-562-Luc2 cells (1 × 10^7 cells in 100 μL PBS) mixed 1:1 (v/v) with a basement membrane matrix (Matrigel®, Corning® 354248) into the right flank of each NOD-SCID-gamma (NSG) mice (n = 8, 4 females and 4 males, with 2 of each sex assigned to HL-60-Luc2 or K-562-Luc2 cell groups). Successful engraftment was confirmed by monitoring tumor progression over time using bioluminescence imaging (BLI) on an IVIS system following intraperitoneal injection of luciferin.

A preliminary pilot test of **NR-HOCl-TFMU** imaging in the presence of HOCl was conducted in Eppendorf tubes to determine optimal combinations of excitation wavelengths and emission filters. Mice were given intratumoral (right flank) injections with **NR-HOCl-TFMU** (100 μL of 100 μM in sterile saline containing 10% DMF), fluorescence images were acquired before and immediately after injections to assess activation and distribution. After *in vivo* imaging, mice were sacrificed according to institutional guidelines. Tumors were excised,

immediately flash frozen in liquid nitrogen, and stored at -80 °C. Tumor tissue slices (400 μm thick) were prepared using a Leica VT1000 S vibrating microtome. The slices were then imaged with a 10 × objective using a Leica STELLARIS 8 laser scanning confocal/spectral imaging microscope to assess fluorescence in HL-60-Luc2 and K-562-Luc2 tumor tissues.

Generation of HL-60-Luc2 MPO-knockout cells by CRISPR/Cas9

To knockout Myeloperoxidase (MPO) by CRISPR/Cas9 in HL-60-Luc2 cells, a sense-stranded guide RNA targeting exon 3 of the MPO gene 5'-ATCCTACTTCAAGCAGCCGG-3' (IDT) was used. Briefly, 100 pmol MPO exon 3 S sgRNA along with 1 μg CleanCap Cas9 mRNA (TriLink) was electroporated into approximately 1 × 10^6 HL-60-Luc2 cells in a 10 μL tip in a Neon NxT electroporator (Invitrogen) and allowed to recover for 4 days at which time a portion of the population was harvested to determine the CRISPR editing efficiency at the MPO exon 3 target site. To determine successful CRISPR/Cas9 editing of MPO exon 3, PCR was performed using two primers that span exon 3 of MPO, MPO exon 3 TIDE ScrF 5'-CCAGGCCTTTCAGAGAAGCA-3' and MPO exon 3 TIDE ScrR 5'-CCTGAGACTCCCTGGAGGAA-3' to produce a 596 bp amplicon by PCR. Resulting amplicons from the CRISPR edited and unedited populations were submitted for Sanger sequencing (Genewiz). Successful editing was confirmed by TIDE analysis showing an editing efficiency of 20%. The successfully edited population was then subcloned by limited dilution at one cell per well onto 4 × 96-well tissue culture plates. Of 29 candidate clones screened, a total of four MPO KOs (#13, #22, #24 and #29) were identified by PCR and Sanger sequencing to contain out-of-frame indels in MPO exon 3. In this study, clones #24 was used which contains homozygous 10 bp deletions produced by a 6 bp (GCAGCC) microhomology sequence at the CRISPR cut site.

Cell Toxicity Assay

HL-60 (30,000 cells/well in IMDM without phenol red + 20% FBS + 1 × Anti-Anti) or K-562 (10,000 cells/well in IMDM without phenol red + 10% FBS + 1 × Anti-Anti) were loaded to the Nunc™ Edge 2.0 96-well plates (Thermo Fisher Scientific, 14-387-220, 100 μL per well), the surrounding moats were filled with sterile water. After incubating at 37 °C in a humidified atmosphere with 5% CO_2 overnight, 100 μL of the corresponding cell culture media containing different concentrations of the indicated probe (DMF stock) was added to each well to achieve a final concentration of 0, 5, 10, 25, 50 μM probe in the cell culture media with 1% DMF. After mixing well and incubating for the indicated time at 37 °C with 5% CO_2, 15 μL CCK-8 was added to each well and the plate was shaken on a mixer for 5 min and then incubated at 37 °C with 5% CO_2 for 2 h. Absorbance intensity at 450 nm was recorded on a microplate reader. Each condition was performed in four replicates and all data were background corrected using the absorbance intensity of CCK-8 in pure cell culture medium (1% DMF) without cells and treated in the same manner. The same toxicity assay protocol was applied to both HL-60-Luc2 wild-type cells (30,000 cells/well in IMDM without phenol red, supplemented with 20% FBS and 1× Anti-Anti) and HL-60-Luc2 MPO-knockout cells.

Synthesis Procedures

Synthesis of NR_{666}-HOCl

NR-HOCl was synthesized following the previously reported method.[4] **NR-HOCl** (400 mg) was dissolved in 6 M HCl, and the solution was refluxed at 100 °C overnight. The aqueous solution was removed by high vac rotary evaporation and the final compound **NR_{666}-HOCl** was further purified through semi-prep HPLC, monitoring at 254 nm. A pale green-white solid was obtained after lyophilization (346 mg, yield: 92%).

^{1}H-NMR (600 MHz, DMSO-d_6): δ 7.50 (d, J = 7.7 Hz, 1H), 7.38-7.34 (m, 1H), 7.24 (t, J = 7.3 Hz, 1H), 7.08 (dd, J = 14.2, 2.9 Hz, 2H), 6.79 (dd, J = 9.1, 2.9 Hz, 2H), 6.74-6.64 (m, 3H), 4.45 (s, 2H), 2.93 (s, 12H).

^{13}C-NMR (151 MHz, DMSO-d_6): δ 148.21, 148.15, 148.06, 141.59, 136.34, 136.29, 130.38, 130.36, 130.28, 129.54, 127.57, 127.44, 126.56, 124.90, 116.00, 110.02, 109.98, 39.97, 37.21.

^{31}P-NMR (243 MHz, DMSO-d_6): δ 11.65.

HRMS (ESI) m/z calculated for Chemical Formula: $C_{24}H_{26}N_2O_2PS$ $[M+H]^+$ 437.1453, found 437.1456.

General Synthetic Conditions for CDI Coupling

NR_{666}-HOCl (0.1 mmol, 1 equiv.), phenol containing fluorophore (1 mmol, 10 equiv.), and carbonyldiimidazole (CDI, 162 mg, 1 mmol, 10 equiv.) were mixed in a vial with the pressure relief cap.[5] The vial evacuated under vacuum and recharged with nitrogen. Anhydrous acetonitrile (3 mL) was then added to dissolve the mixture followed by dropwise addition of 127 µL N,N-dimethylaniline (1 mmol, 10 equiv.). The vial was evacuated by vacuum and recharged with nitrogen again before heating to 50 °C. After overnight reaction, the solvent was removed by rotovap, and then the mixture was dissolved in HPLC buffer (50% acetonitrile in water with 0.1% trifluoroacetic acid). After centrifugation and filtering, the sample was purified through semi-prep HPLC, monitoring at 254 nm, and the product peak from HPLC was lyophilized to afford the final product.

Synthesis of NR-HOCl-4MU

NR_{666}-HOCl (0.1 mmol, 43.6 mg) was reacted with **4MU** (1 mmol, 176 mg) and the product peak from HPLC was lyophilized to afford **NR-HOCl-4MU** as a pale white-yellow solid (46.4 mg, 78%).

^{1}H-NMR (400 MHz, DMSO-d_6): δ 7.74 (d, J = 8.8 Hz, 1H), 7.54 (d, J = 8.2 Hz, 1H), 7.42 (t, J = 7.5 Hz, 1H), 7.30 (t, J = 7.2 Hz, 1H), 7.25-7.20 (m, 1H), 7.17-7.09 (m, 3H), 6.87 (dd, J = 9.2, 3.1 Hz, 2H), 6.76 (d, J = 7.7 Hz, 1H), 6.64 (t, J = 8.4 Hz, 2H), 6.37-6.26 (m, 1H), 4.46 (s, 2H), 2.90 (s, 12H), 2.38 (s, 3H).

^{13}C-NMR (101 MHz, DMSO-d_6): δ 159.56, 158.50, 158.12, 153.53, 153.39, 153.31, 152.97, 148.13, 147.99, 146.82, 142.34, 137.07, 136.99, 130.25, 130.13, 127.87, 127.77, 126.76, 126.73, 125.74, 125.03, 124.46, 117.33, 117.29, 117.20, 116.46, 113.14, 111.18, 111.11, 108.51, 108.46, 65.22, 65.12, 37.09, 18.11.

^{31}P-NMR (162 MHz, DMSO-d_6): δ 19.05.

HRMS (ESI) m/z calculated for Chemical Formula: $C_{34}H_{32}N_2O_4PS$ $[M+H]^+$ 595.1820, found 595.1825.

Synthesis of NR-HOCl-TFMU

NR$_{666}$-HOCl (0.1 mmol, 43.6 mg) and **TFMU** (1 mmol, 230 mg) were used for this reaction. After lyophilization, 47.3 mg of a pale white-green solid was afforded with a yield of 73%.

^{1}H-NMR (600 MHz, DMSO-d_6): δ 7.16 (d, J = 7.6 Hz, 1H), 6.99 (d, J = 7.7 Hz, 1H), 6.89-6.85 (m, 1H), 6.82 (dd, J = 8.9, 2.1 Hz, 1H), 6.77-6.73 (m, 1H), 6.69 (d, J = 2.0 Hz, 1H), 6.59 (dd, J = 15.0, 2.9 Hz, 2H), 6.42 (s, 1H), 6.31 (dd, J = 9.1, 3.0 Hz, 2H), 6.22 (d, J = 7.6 Hz, 1H), 6.07 (dd, J = 8.9, 8.0 Hz, 2H), 3.89 (s, 2H), 2.35 (s, 12H).

^{13}C-NMR (151 MHz, DMSO-d_6): δ 158.36, 158.18, 158.12, 154.59, 154.16, 148.21, 148.12, 146.66, 142.41, 136.96, 136.90, 130.17, 130.09, 127.89, 127.76, 126.77, 126.22, 126.22, 125.36, 125.04, 124.51, 118.17, 118.14, 117.19, 116.02, 115.98, 111.11, 109.90, 109.24, 109.20, 65.10, 39.77, 37.05.

^{19}F-NMR (564 MHz, DMSO-d_6): δ -65.37, -76.55 (TFA).

^{31}P-NMR (243 MHz, DMSO-d_6): δ 19.54.

HRMS (ESI) m/z calculated for $C_{34}H_{29}F_3N_2O_4PS$ $[M+H]^+$ 649.1538, found 649.1542.

Figure S1

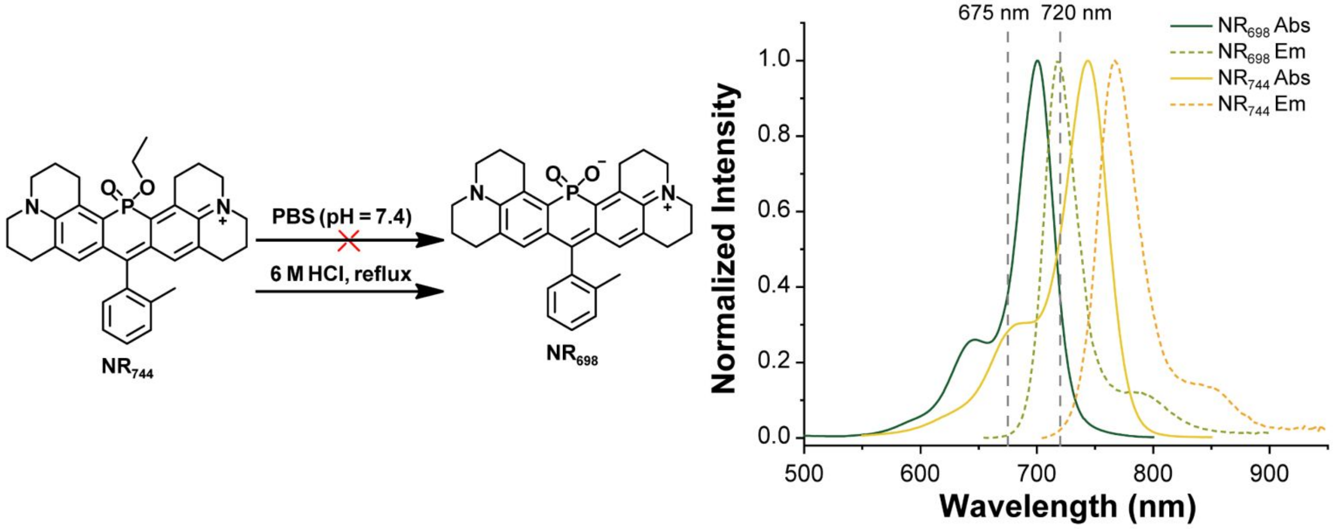

The phosphinate ester in $\mathbf{NR_{744}}$ is stable in PBS (pH = 7.4) and requires reflux in 6 M HCl to form appreciable amounts of the corresponding phosphinate, $\mathbf{NR_{698}}$ (left). Normalized absorbance and emission spectra of $\mathbf{NR_{744}}$ (PBS at pH = 7.4 with 1% DMSO) or $\mathbf{NR_{698}}$ (acquired by refluxing $\mathbf{NR_{744}}$ in 6 M HCl) are shown (right).

Figure S2

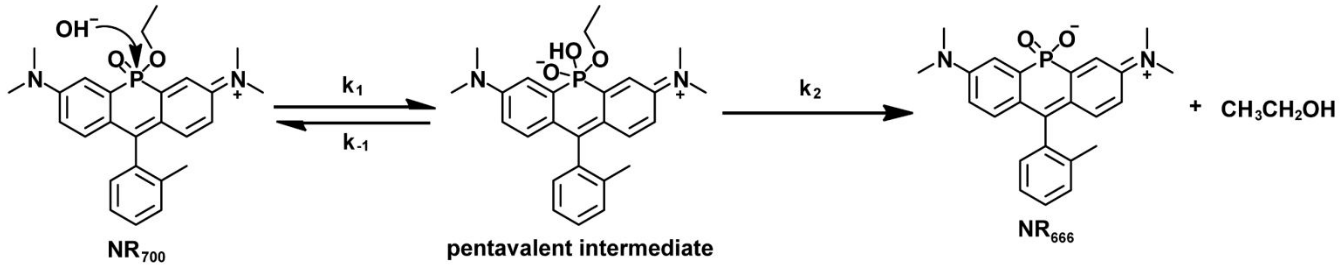

Proposed mechanism for hydrolysis of the phosphinate ester in **NR_{700}** via nucleophilic attack of hydroxide anion in aqueous solution.

Figure S3

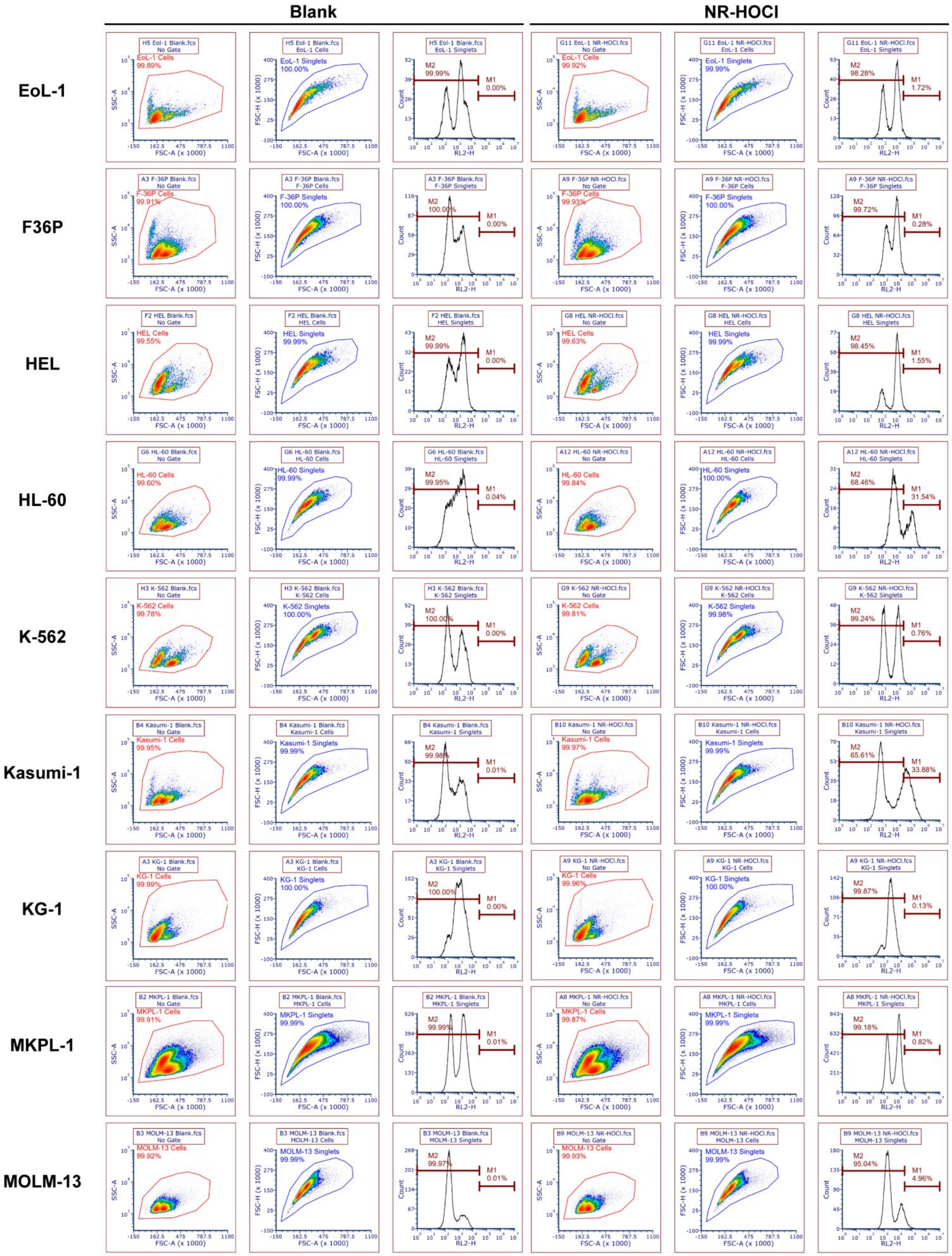

Blank
NR-HOCl
EoL-1
F36P
HEL
HL-60
K-562
Kasumi-1
KG-1
MKPL-1
MOLM-13

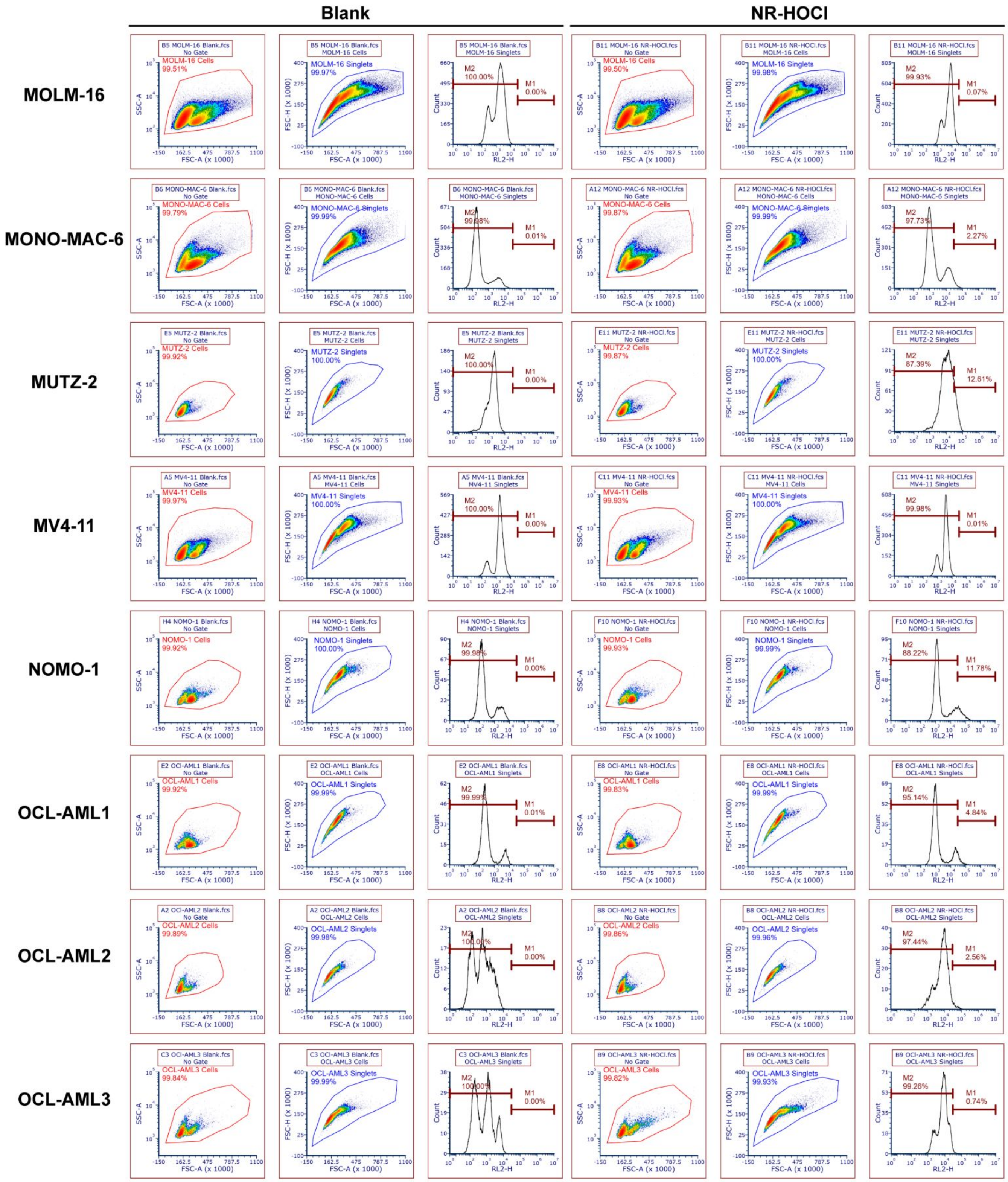
Blank
NR-HOCl
MOLM-16
MONO-MAC-6
MUTZ-2
MV4-11
NOMO-1
OCL-AML1
OCL-AML2
OCL-AML3

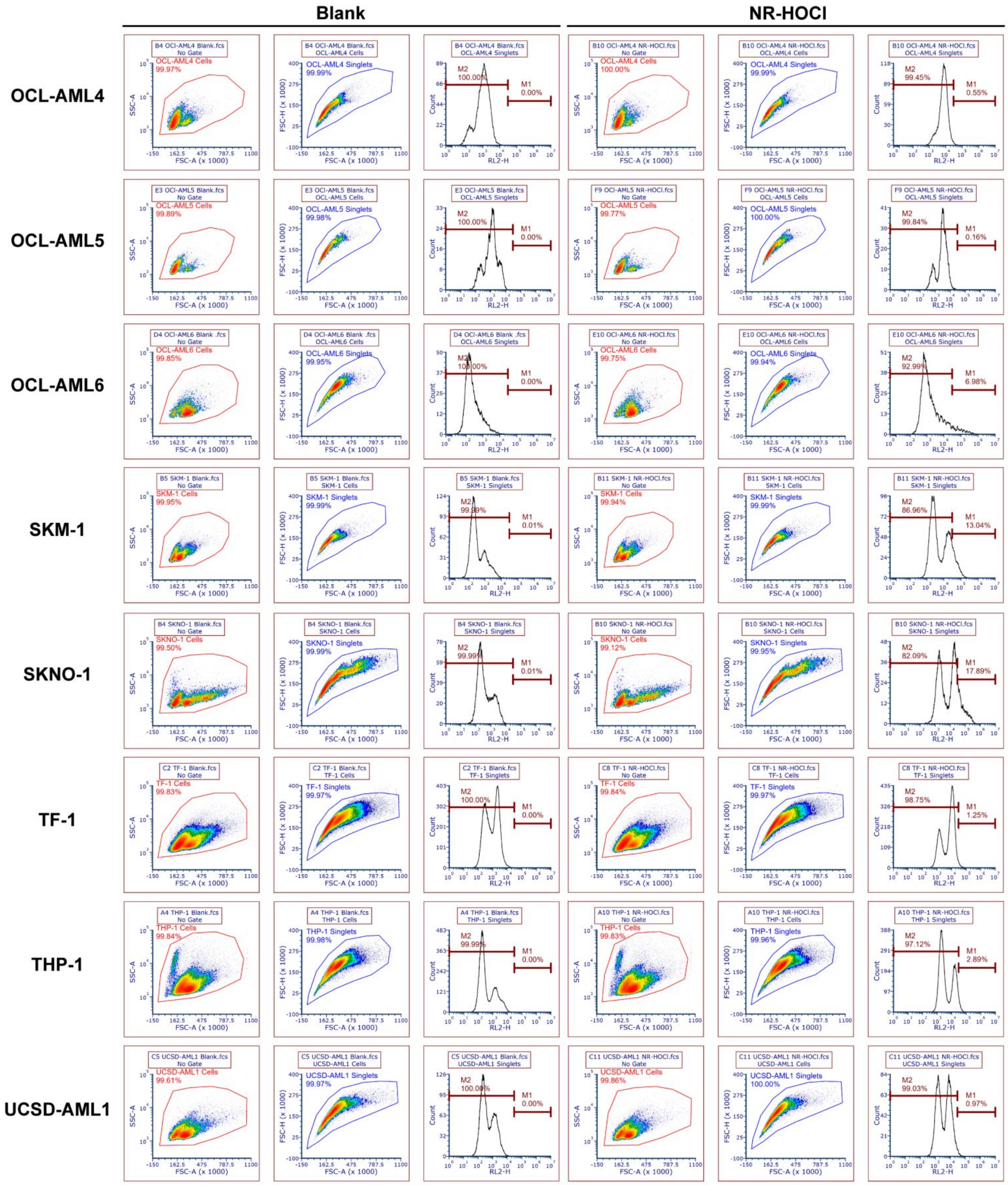

Flow cytometry analysis of the indicated AML cell lines incubated with or without **NR-HOCl** for 1 h at 37 °C in a CO_2 incubator to assess the percentage of NIR fluorescent cells. For each AML cell line, flow cytometry plots include forward scatter (FSC-A) vs side scatter (SSC-A) for cell population gating, FSC-H vs FSC-A for singlet gating, and histograms showing RL2-H NIR fluorescence intensity (RL2 channel: excitation at 637 nm, emission filter 720/30 nm) versus cell count. The gated region **M1** represents the percentage of NIR fluorescent cells.

Figure S4

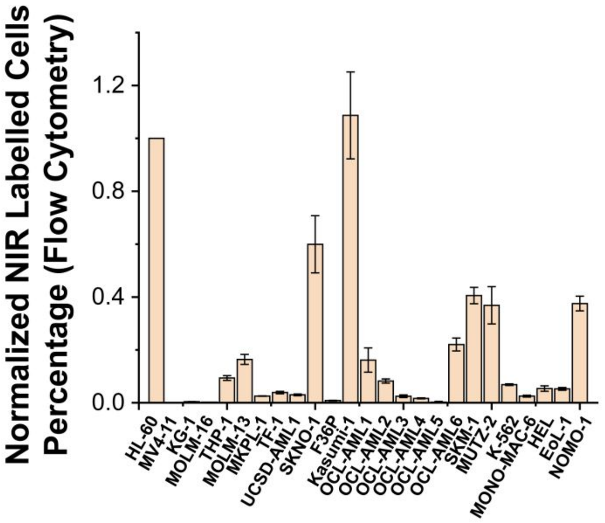

Quantification of relative NIR fluorescence from **NR-HOCl** across different AML cell lines by flow cytometry. Data were obtained from **Figure S3** and normalized to HL-60. Error bars represent mean ± SD from three independent experiments.

Figure S5

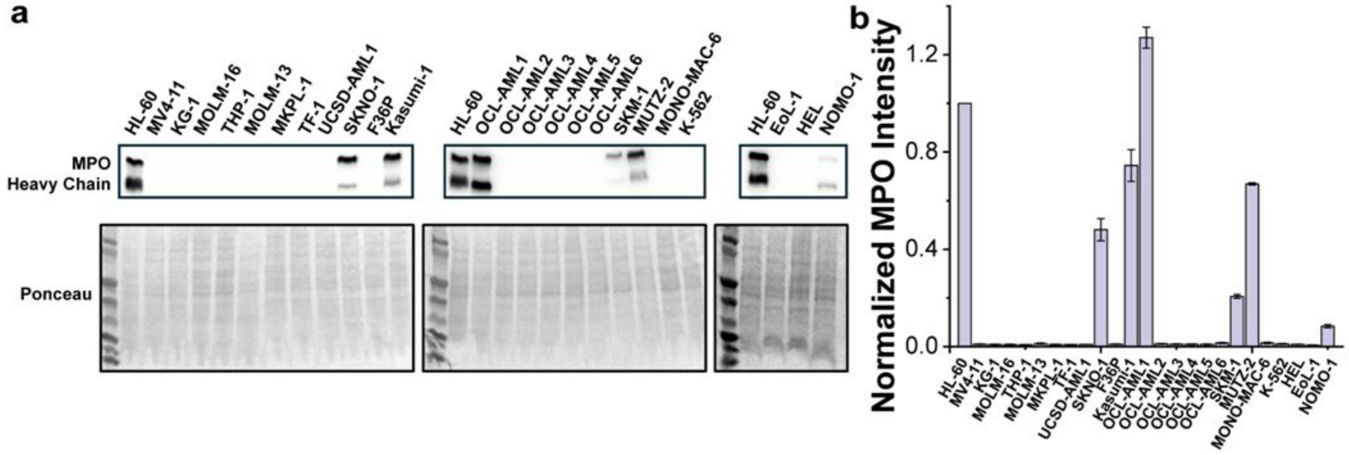

Western blot analysis of MPO expression in the indicated AML cell lines. (**a**) Lysates from AML cell lines were analyzed for MPO expression using the Myeloperoxidase (E1E7I) XP® Rabbit mAb (top). Equal protein loading was verified by Ponceau S staining (bottom). (**b**) Quantification of MPO band intensity by densitometry, cell lines are normalized to HL-60. Data represent mean ± SD from three independent experiments.

Figure S6

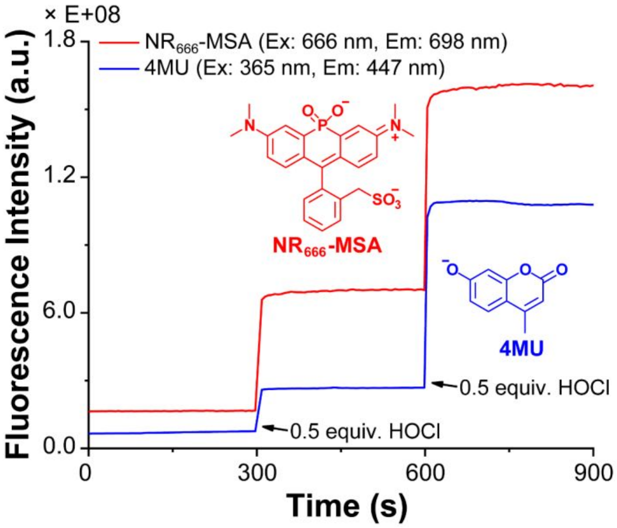

Sequential addition of 0.5 equiv. HOCl to **NR-HOCl-4MU** (5 μM) in PBS (pH = 7.4 with 1% DMF). Fluorescence intensity of the reaction products, **NR_{666}-MSA** and **4MU**, was monitored over time.

Figure S7

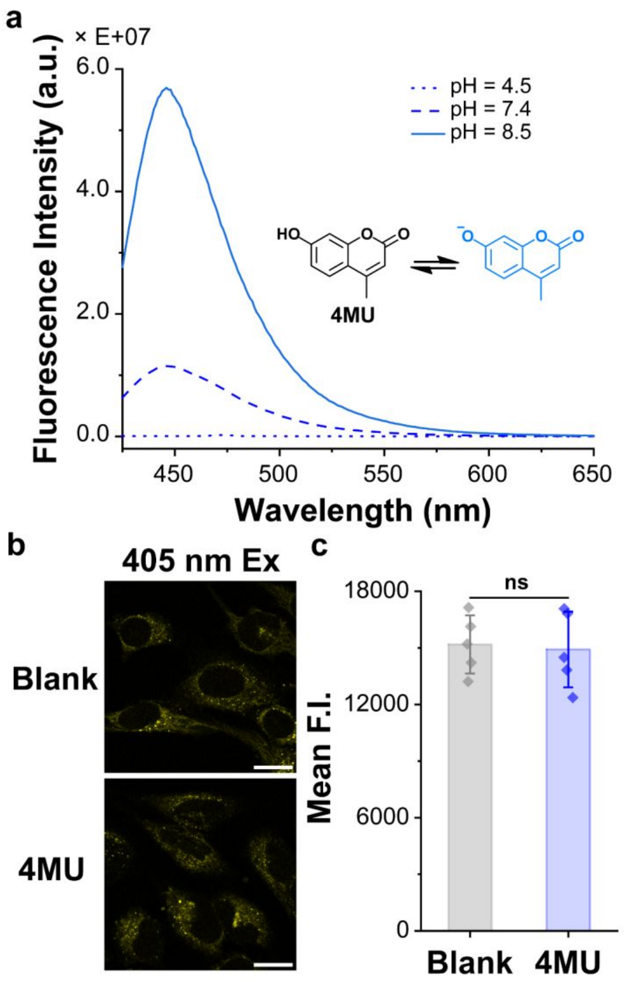

4MU fluorescence is difficult to distinguish from cellular auto fluorescence. (**a**) Fluorescence spectra of **4MU** (10 μM) in different pH buffer solutions (1% DMSO, Ex: 405 nm). (**b**) Confocal fluorescence images of HeLa cells stained without **4MU** (0 μM, 1% DMSO, Blank) or with **4MU** (10 μM, 1% DMSO), scale bar: 20 μm. (**c**) The mean pixel intensity of images with or without staining with **4MU** across 5 biological replicates. Error bars = SD of mean pixel intensity for each image across 5 biological replicates. Statistical significance was determined using a two-tailed t-test (ns indicates a p-value of > 0.05)

Figure S8

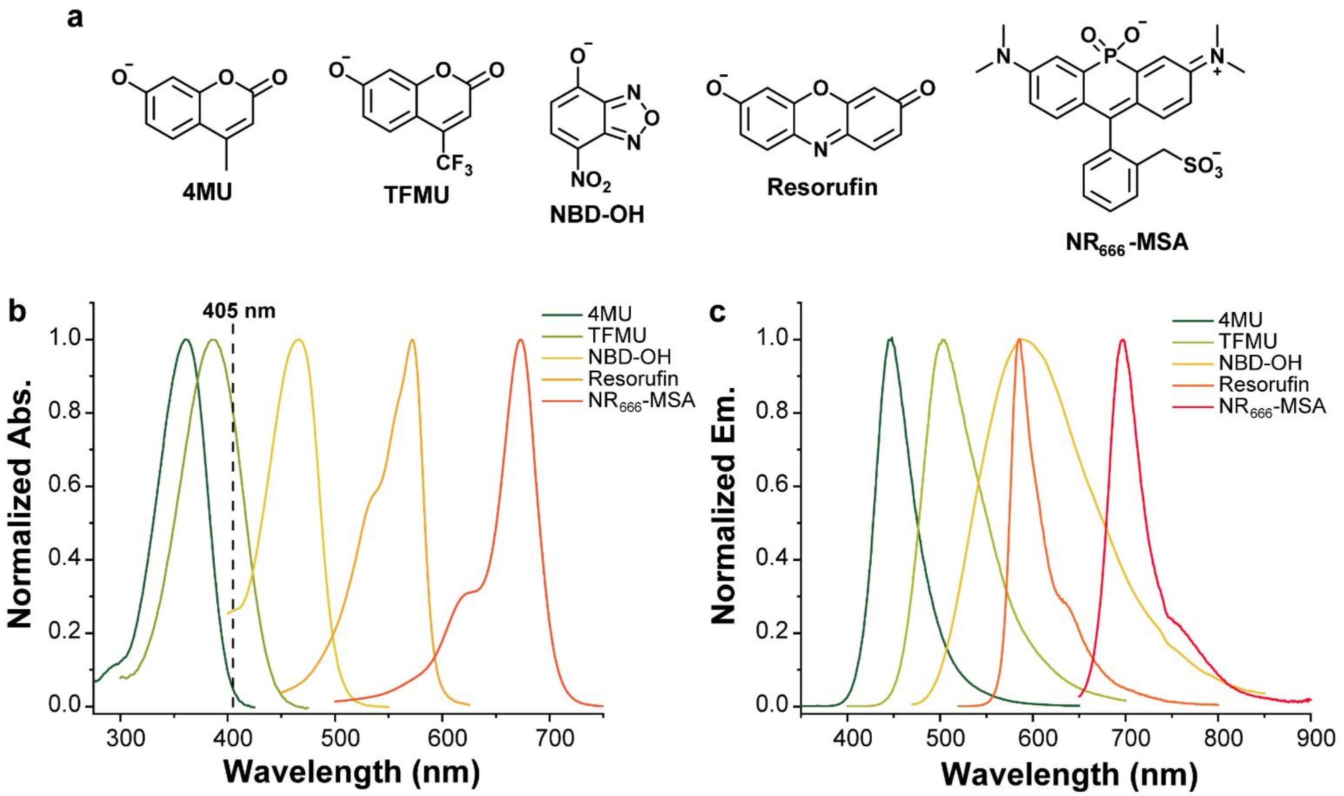

Excitation and emission spectra of phenol-containing cargo dyes versus **NR$_{666}$-MSA**. (**a**) Chemical structures of phenol-containing fluorophores and **NR$_{666}$-MSA**. (**b**) Normalized absorbance spectra of fluorophores from panel **a** in pH = 9.0 buffer. (**c**) Normalized emission spectra of fluorophores form panel **a** in pH = 9.0 buffer.

Figure S9

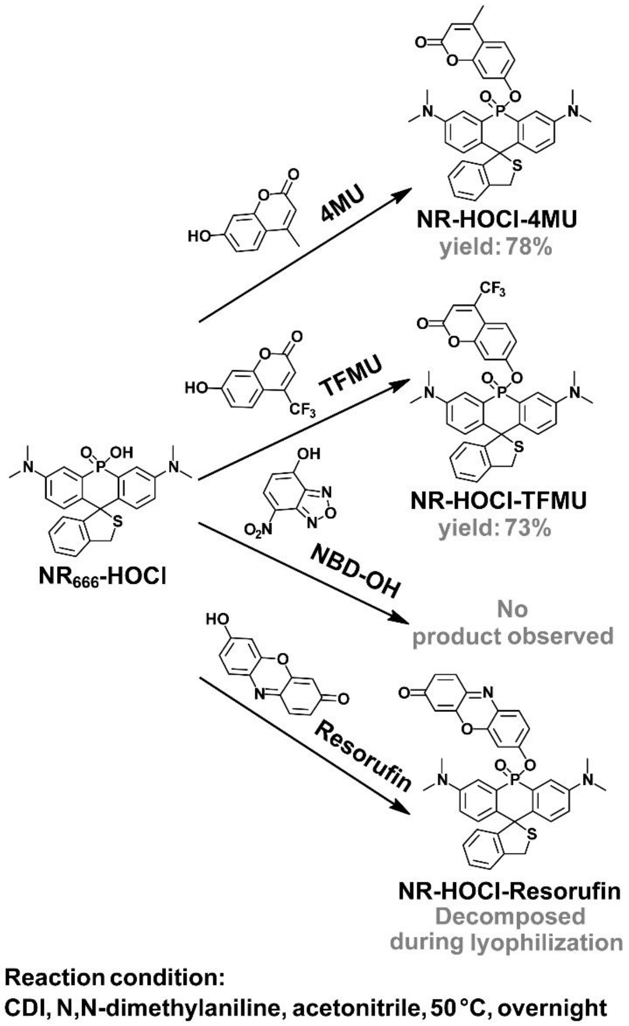

Synthetic scheme for CDI coupling of **NR_{666}-HOCl** with different phenol-containing fluorophores.[5]

Figure S10

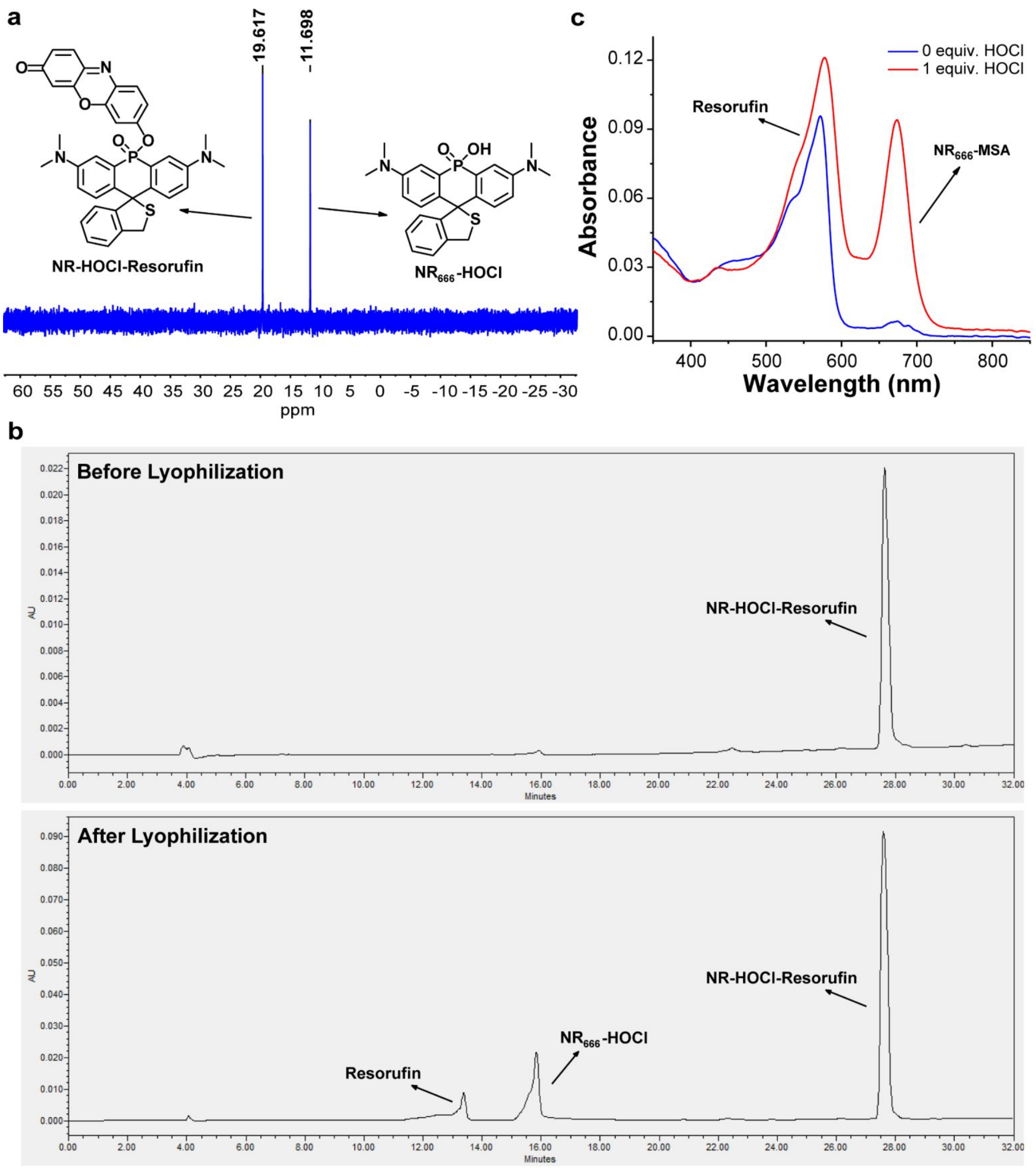

Decomposition of **NR-HOCl-Resorufin** during lyophilization. (**a**) ^{31}P-NMR of lyophilized solid **NR-HOCl-Resorufin** from HPLC in DMSO-d_6 (243 MHz). (**b**) Analytical HPLC of **NR-HOCl-Resorufin** collected from semi-prep HPLC purification before and after lyophilization, monitored at 254 nm. (**c**) Absorbance spectra of **NR-HOCl-Resorufin** (10 μM) in PBS (pH = 7.4 with 1% DMF) with and without adding 1 equiv. HOCl.

Figure S11

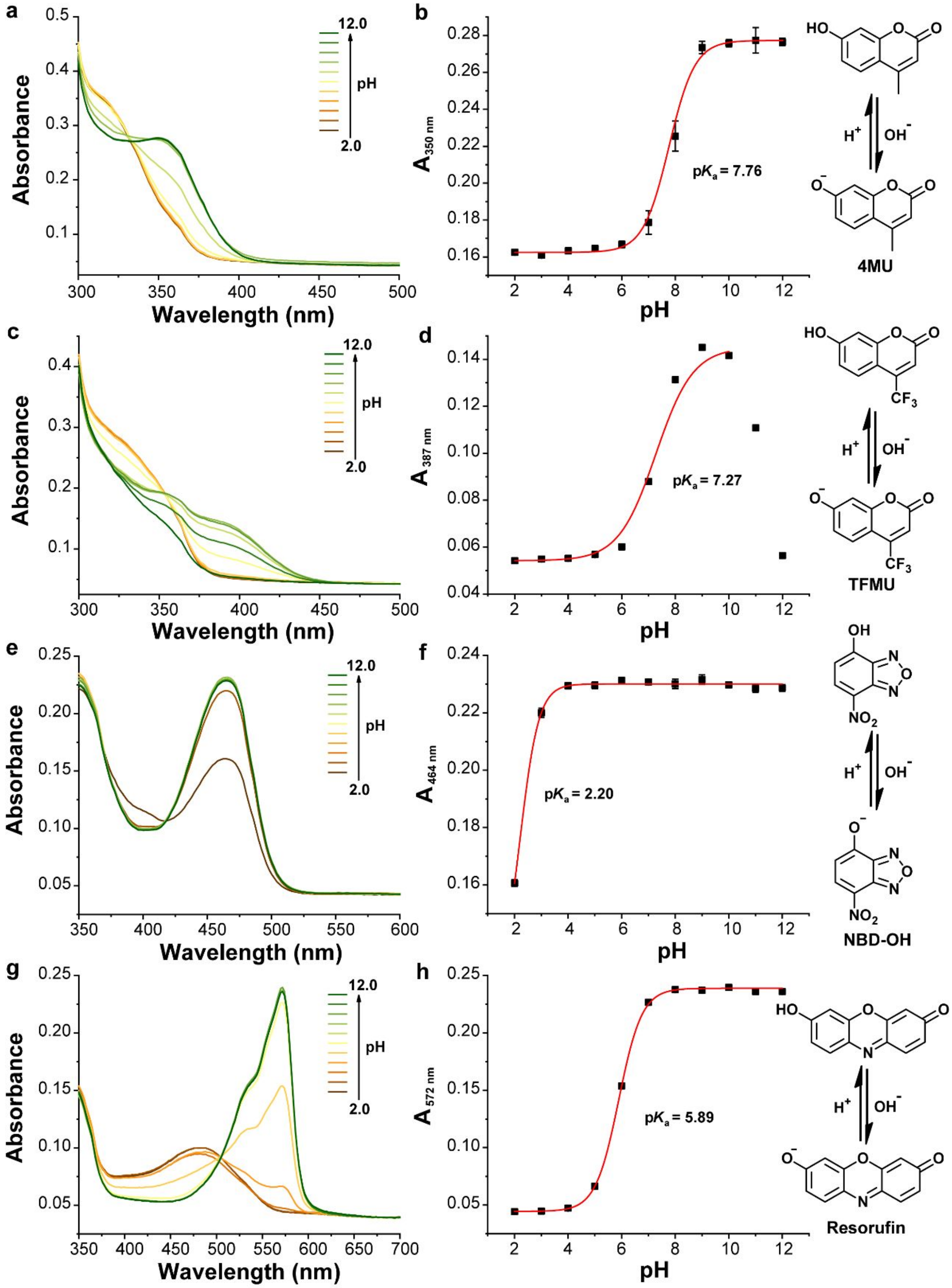

The absorbance of phenol-containing fluorophores (10 μM with 1% DMSO) in Britton-Robinson buffer at the indicated pH. (**a**) (**c**) (**e**) (**g**) are full absorbance spectra. (**b**) (**d**) (**f**) (**h**) show the change in maximal absorbance with pH and the corresponding phenol pK_a.

Figure S12

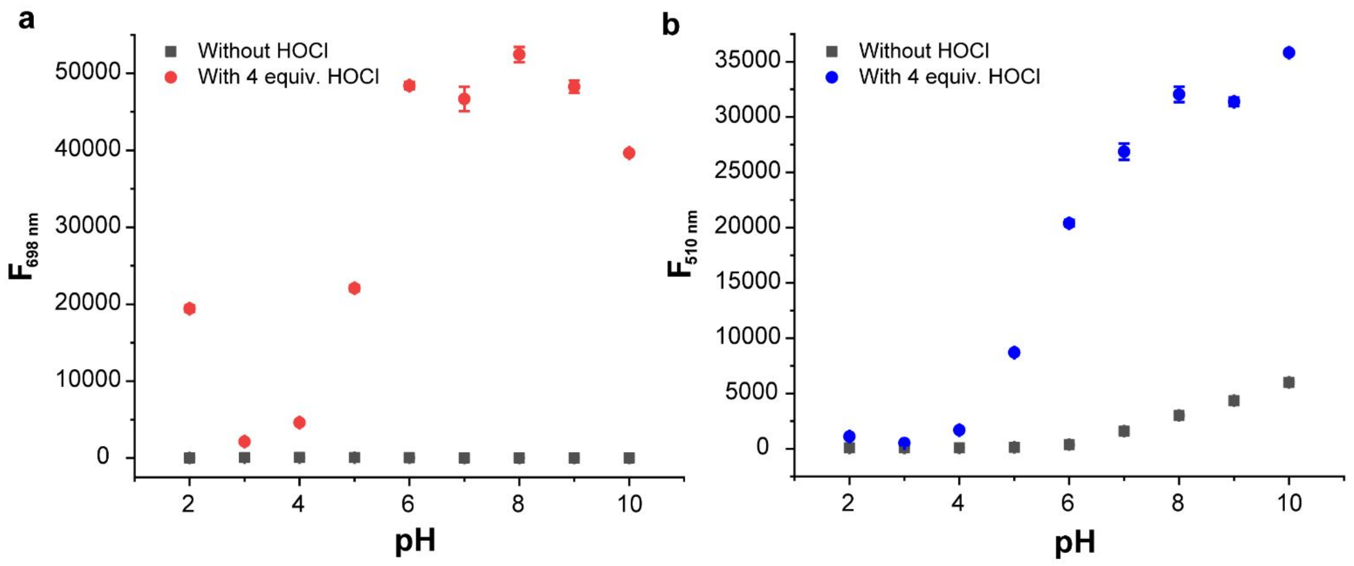

pH-dependent assay of **NR-HOCl-TFMU** (10 μM) with or without 4 equiv. HOCl in different pH buffers (containing 1% DMF). (**a**) **NR_{666}-MSA** (Ex: 640 nm, Em: 698 nm) and (**b**) **TFMU** (Ex: 387 nm, Em: 510 nm).

Figure S13

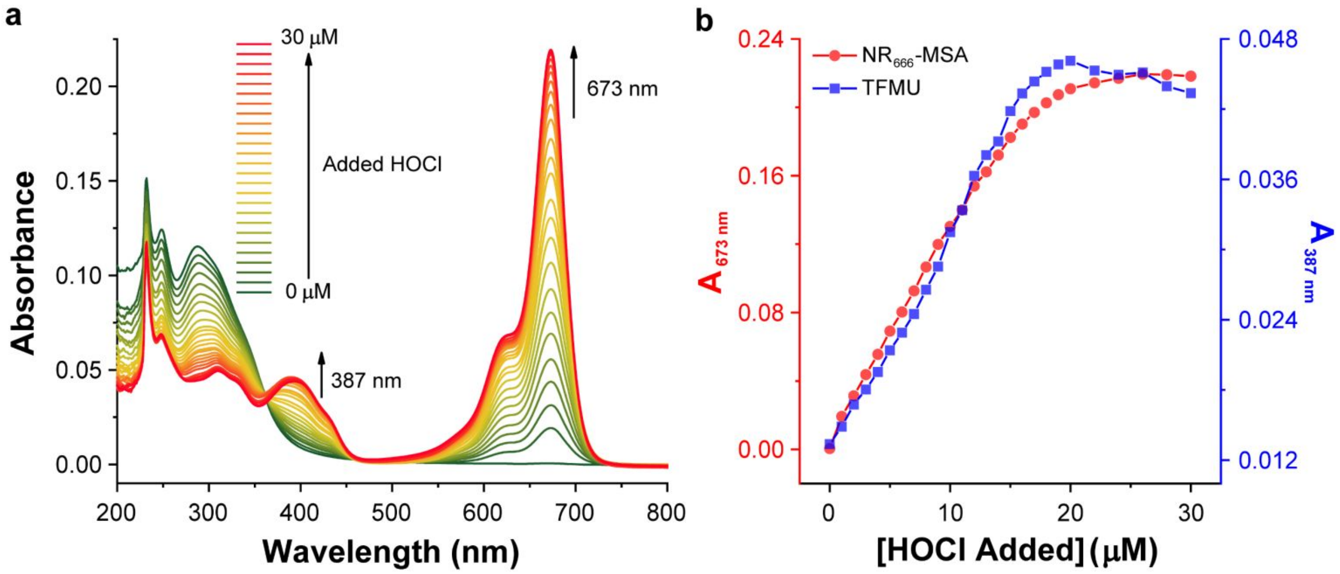

HOCl-gated formation of **TFMU** and **NR_{666}-MSA** from **NR-HOCl-TFMU** as monitored by absorbance. (**a**) The change in absorbance of **NR-HOCl-TFMU** (5 μM) in PBS (pH = 7.4 with 1% DMF) in the presence of different concentrations of HOCl (0 - 6 equiv.). (**b**) The absorbance intensity of **NR_{666}-MSA** (673 nm) and **TFMU** (387 nm) as a function of HOCl concentration from panel **a**.

Figure S14

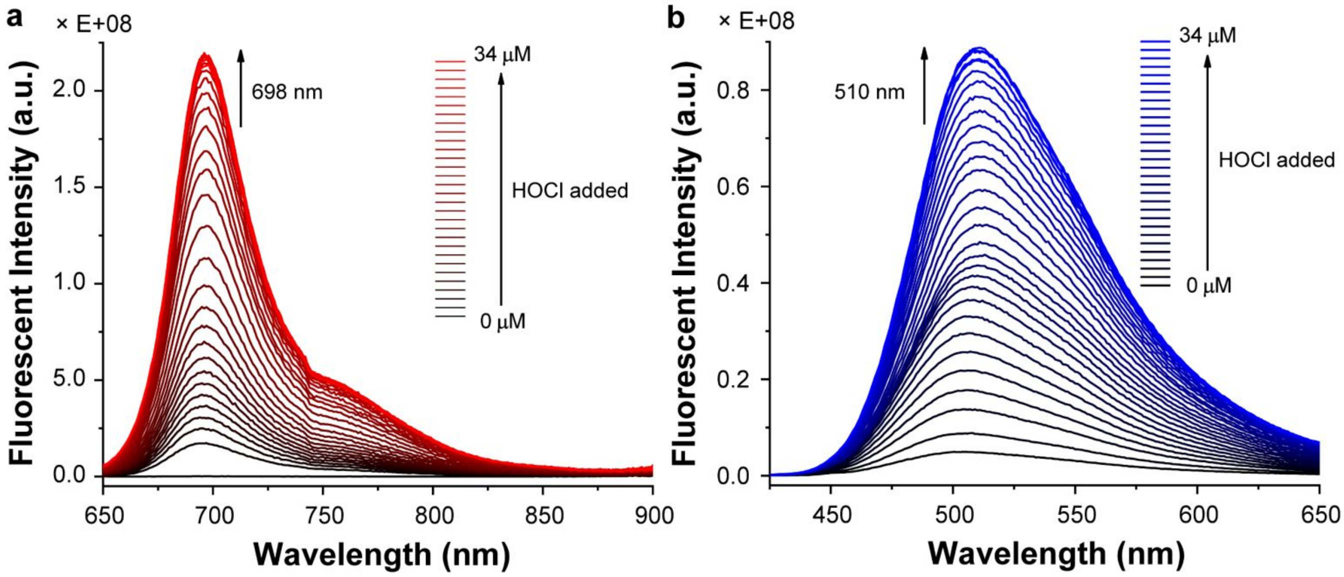

Fluorescence emission spectra of (**a**) **NR_{666}-MSA** (Ex: 640 nm) and (**b**) **TFMU** (Ex: 387 nm) upon addition of different amounts of HOCl (0 - 6.8 equiv.) to **NR-HOCl-TFMU** (5 μM) in PBS (pH = 7.4 with 1% DMF).

Figure S15

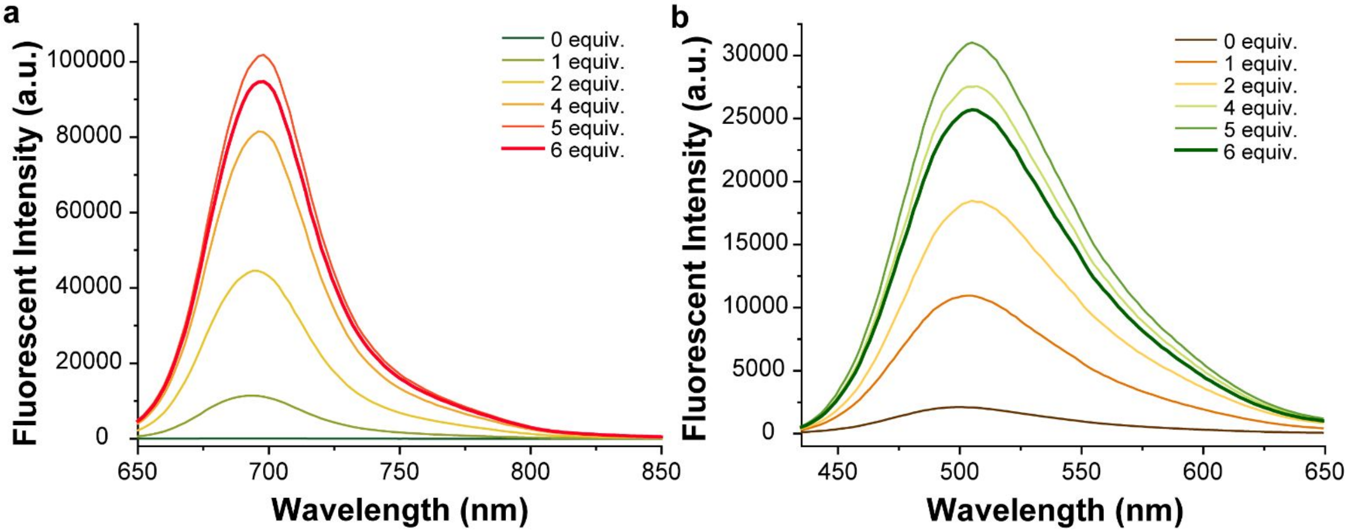

Fluorescence response of **NR-HOCl-TFMU** (10 μM) obtained by mixing equal volumes of **NR-HOCl-TFMU** with the indicated equivalent of HOCl. Experiments were conducted in PBS (pH = 7.4 with 1% DMF). (**a**) **NR_{666}-MSA** (Ex: 605 nm) and (**b**) **TFMU** (Ex: 387 nm).

Figure S16

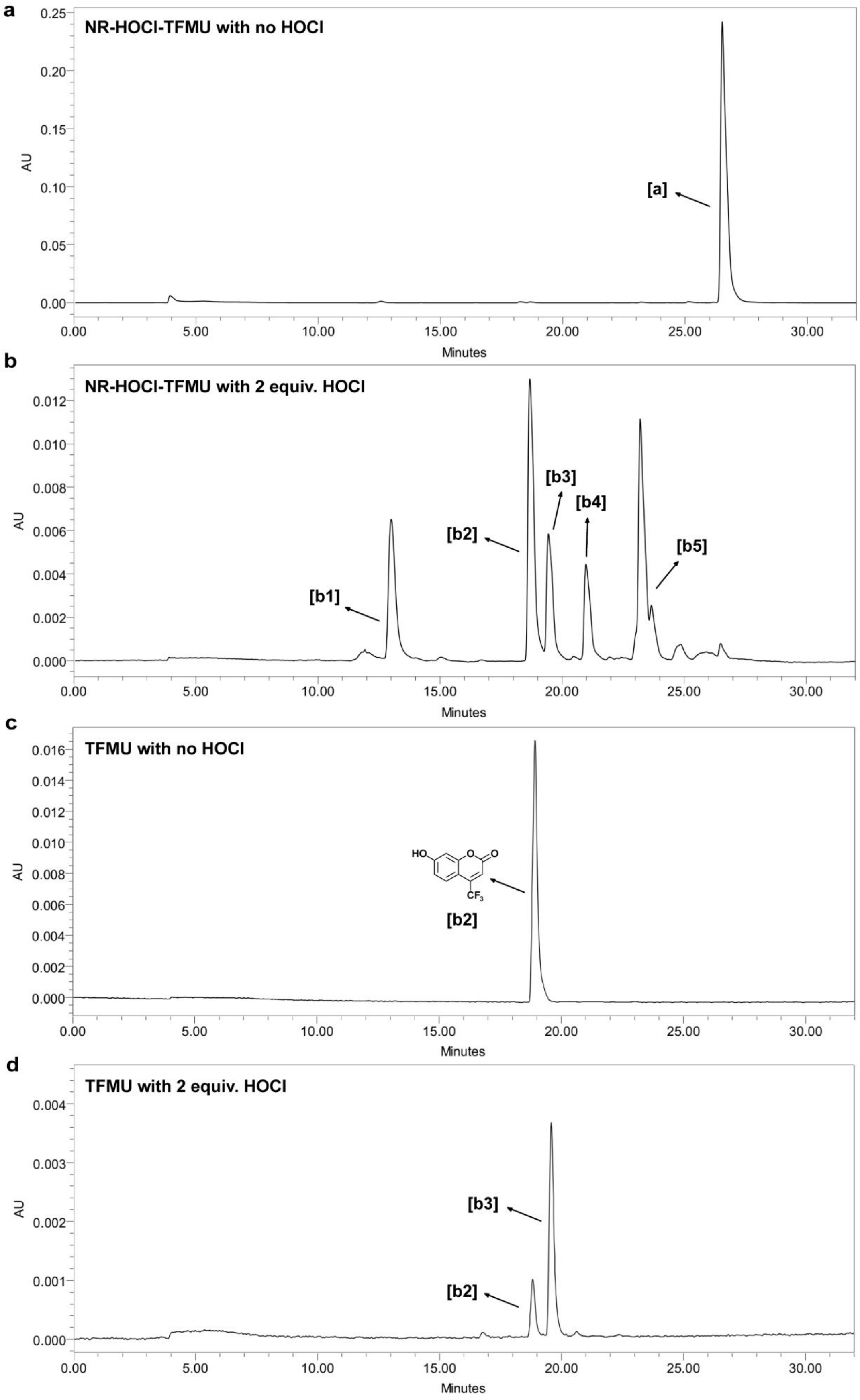
a
NR-HOCl-TFMU with no HOCl
0.25
0.20
0.15
0.10
0.05
0.00
AU
[a]
0.00
5.00
10.00
15.00
20.00
25.00
30.00
Minutes
b
NR-HOCl-TFMU with 2 equiv. HOCl
0.012
0.010
0.008
0.006
0.004
0.002
0.000
AU
[b1]
[b2]
[b3]
[b4]
[b5]
0.00
5.00
10.00
15.00
20.00
25.00
30.00
Minutes
c
TFMU with no HOCl
0.016
0.014
0.012
0.010
0.008
0.006
0.004
0.002
0.000
AU
HO
O
O
CF3
[b2]
0.00
5.00
10.00
15.00
20.00
25.00
30.00
Minutes
d
TFMU with 2 equiv. HOCl
0.004
0.003
0.002
0.001
0.000
AU
[b3]
[b2]
0.00
5.00
10.00
15.00
20.00
25.00
30.00
Minutes

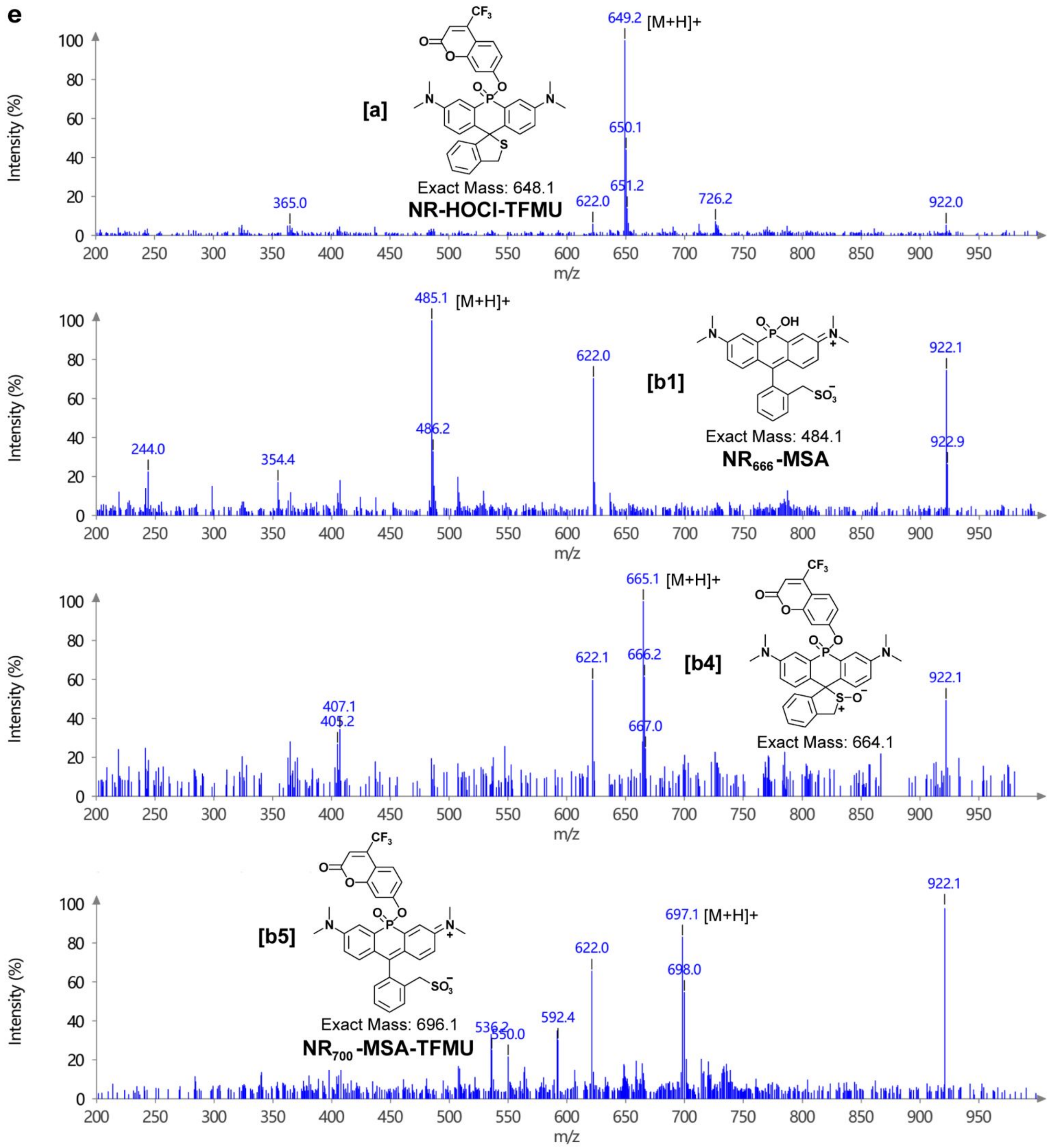

Conversion of **NR-HOCl-TFMU** to products upon treatment with HOCl. Analytical HPLC purification of 500 µM **NR-HOCl-TFMU** before (**a**) and after (**b**) treatment with 2 equiv. of HOCl. Based on retention time comparison with authentic **TFMU** (**c**), [b2] is assigned as the released payload **TFMU**, and [b3] is tentatively assigned as a side product formed between **TFMU** and HOCl (**d**). (**e**) Corresponding MS spectra and proposed chemical structures (not confirmed by NMR) are shown for collected peaks [a], [b1], [b4], and [b5].[6] Peaks [b2] and [b3] displayed very low MS intensity and are not shown. Analytical HPLC was performed using a water/acetonitrile gradient containing 0.1% TFA, v/v starting from 95:5 to 5:95 over 30 min, monitored at 254 nm.

Figure S17

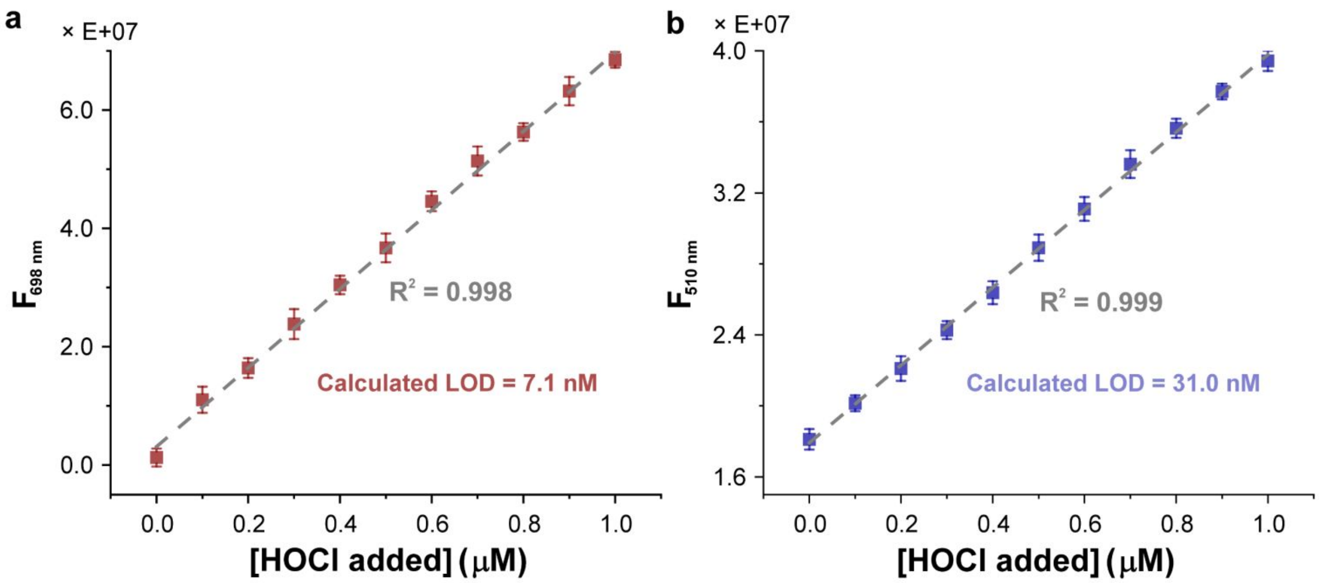

Determination of the detection limit of **NR-HOCl-TFMU** (5 μM in PBS, pH = 7.4 with 1% DMF) for HOCl. **NR-HOCl-TFMU** was incubated with increasing concentrations of HOCl (0-1 μM), and the resulting fluorescence intensity was measured at 698 nm for **NR_{666}-MSA** (**a**) and at 510 nm for **TFMU** (**b**). The detection limit (LOD) was calculated using the 3σ/k method, where σ is the standard deviation of the blank and k is the slope of the linear calibration curve. Error bars represent the standard deviation of triplicate measurements.

Figure S18

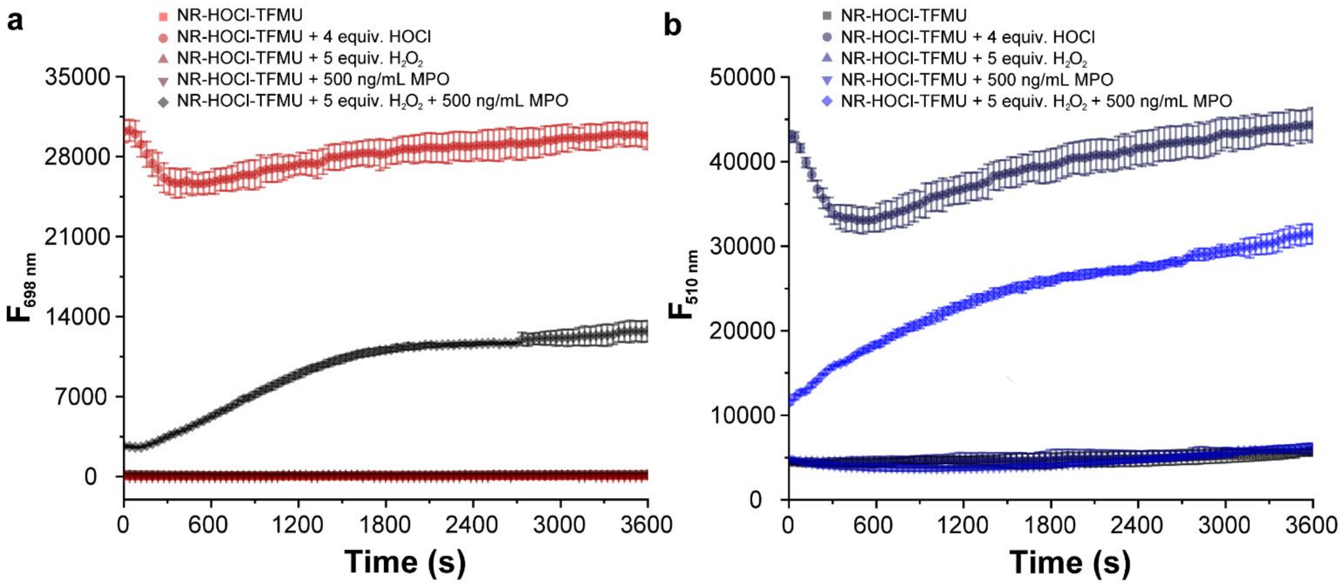

Fluorescence response of **NR-HOCl-TFMU** (10 μM) to HOCl (4 equiv.), H_2O_2 (5 equiv.), MPO (500 ng/mL), or HOCl generated by H_2O_2 (5 equiv.) and MPO (500 ng/mL) in PBS (pH = 7.4 with 1% DMF). (**a**) **NR_{666}-MSA** fluorescence (Ex: 640 nm, Em: 698 nm) and (**b**) **TFMU** fluorescence (Ex: 387 nm, Em: 510 nm).

Figure S19

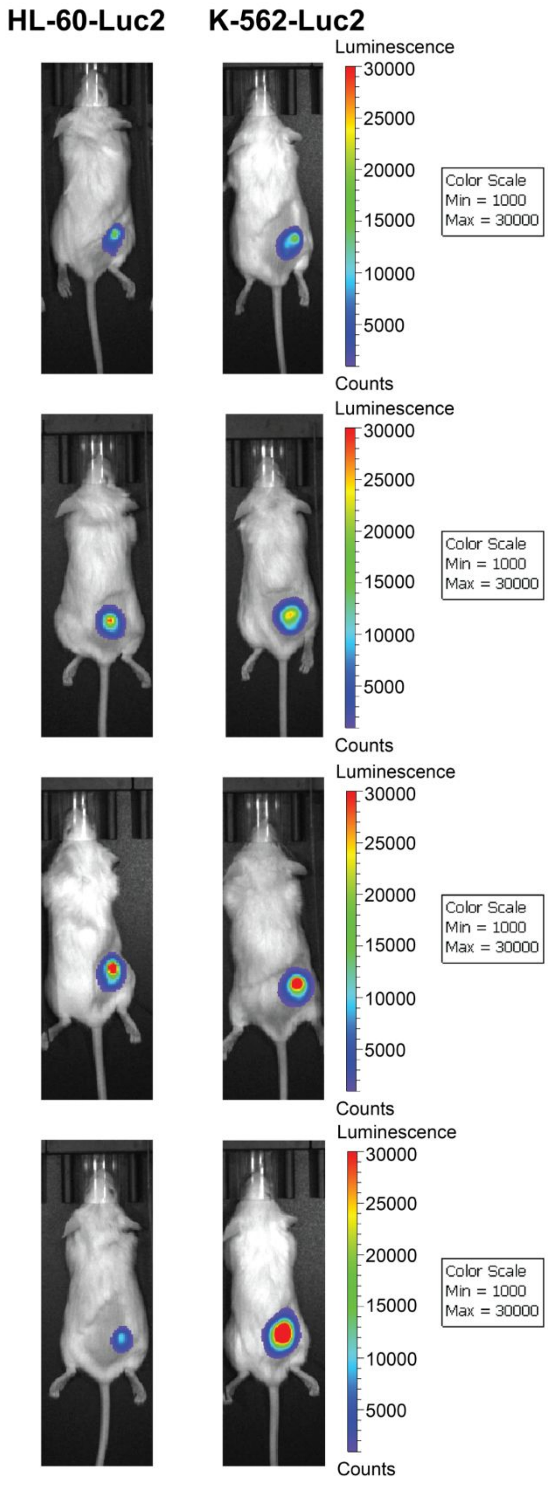

Bioluminescence imaging inoculation of HL-60-Luc2 and K-562-Luc2 tumor-bearing mice. Representative luminescence images of mice bearing subcutaneous HL-60-Luc2 (left) or K-562-Luc2 (right) tumors. The intensity of luminescence (color-coded heat map) reflects tumor cell burden, with a fixed color scale ranging from 1,000 to 30,000 counts.

Figure S20

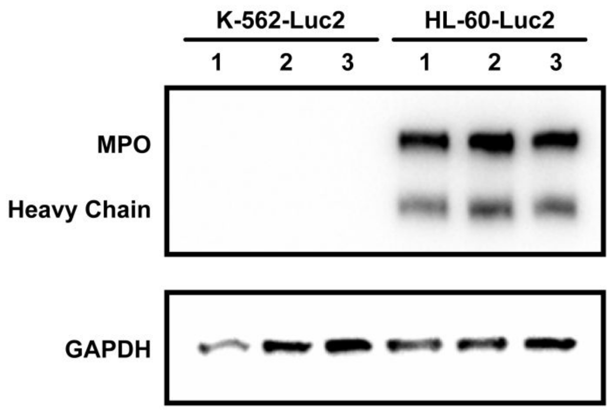

Western blot analysis of lysates from HL-60-Luc2 or K-562-Luc2 cells probed for MPO expression using the Myeloperoxidase (E1E7I) XP® Rabbit mAb (top). Equivalent amounts of total protein were loaded in each lane as confirmed by GAPDH staining (bottom).

Figure S21

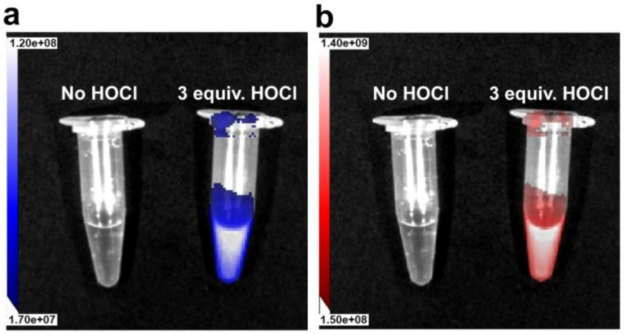

In vitro fluorescence imaging of 100 μM **NR-HOCl-TFMU** with or without 3 equiv. HOCl in eppendof tubes using an IVIS imaging system. Optimal excitation and emission filters were 430 and 520 nm for **TFMU** (**a**) and 675 and 760 nm for **NR$_{666}$-MSA** (**b**), respectively.

Figure S22

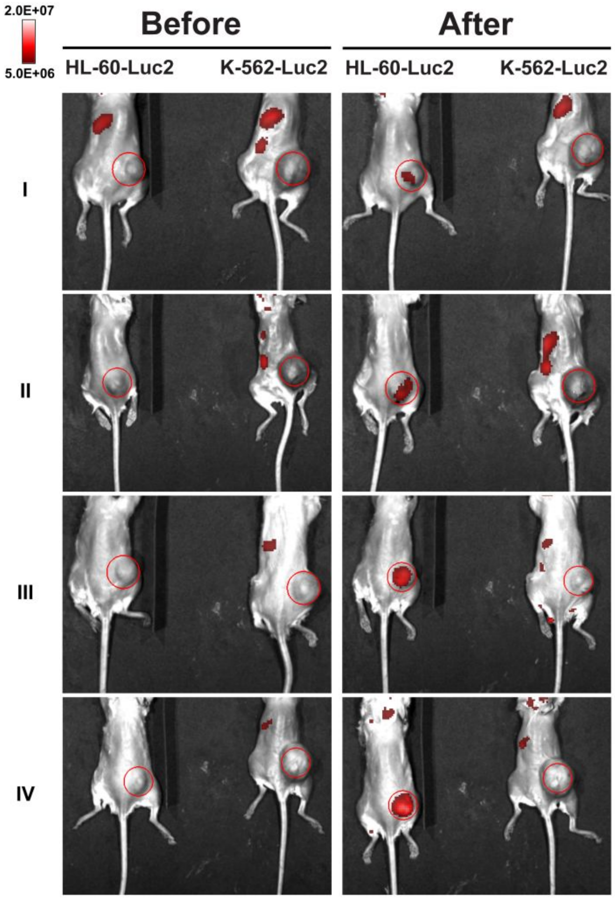

In vivo NIR fluorescence imaging of HL-60-Luc2 and K-562-Luc2 tumor-bearing mice before and immediately after injection of 100 μM **NR-HOCl-TFMU** in sterile saline (with 10% DMF). Images were acquired before and after intratumoral injection of the **NR-HOCl-TFMU** using 675 nm excitation and 760 nm emission filters. Fluorescence signal in tumors is indicated by red circles. The color scale represents total radiant efficiency (TRE) ([p/s]/[μW/cm^2]).

Figure S23

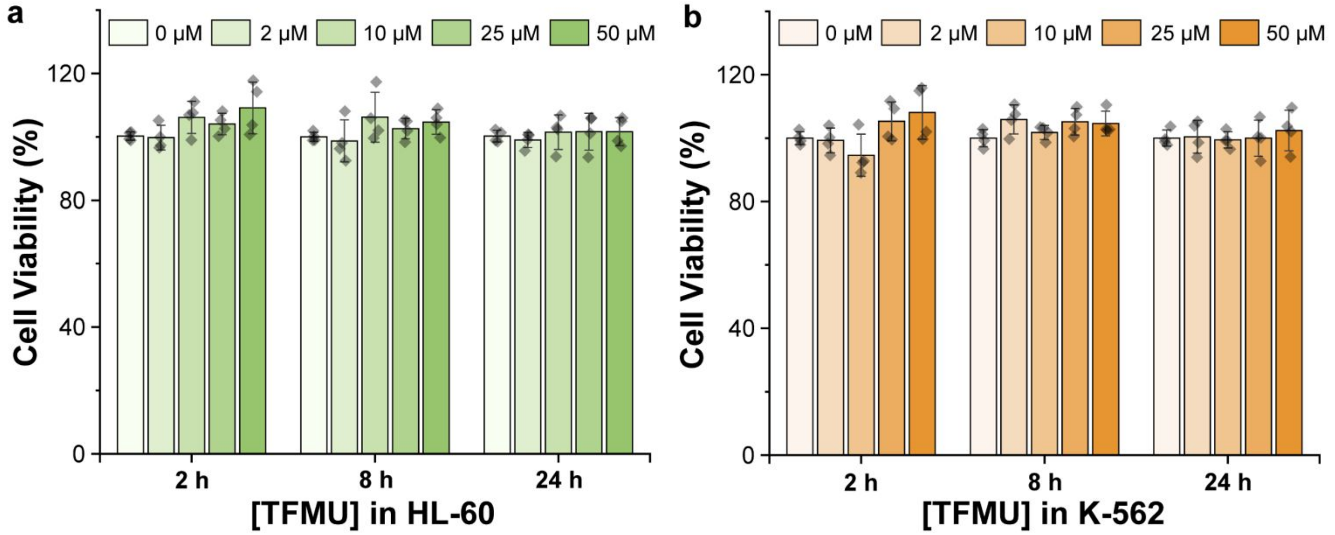

Toxicity of **TFMU** in HL-60 (**a**) or K-562 (**b**) cells. Cells were incubated with the indicated concentration of **TFMU** (containing 1% DMF) in the corresponding cell culture media without phenol red. Cell viability was assessed at the indicated time point using the commercially available CCK-8 assay. Data represent four biological replicates. Error bars indicate mean ± SD. No statistically significant differences were observed using a two-tailed t-test.

Figure S24

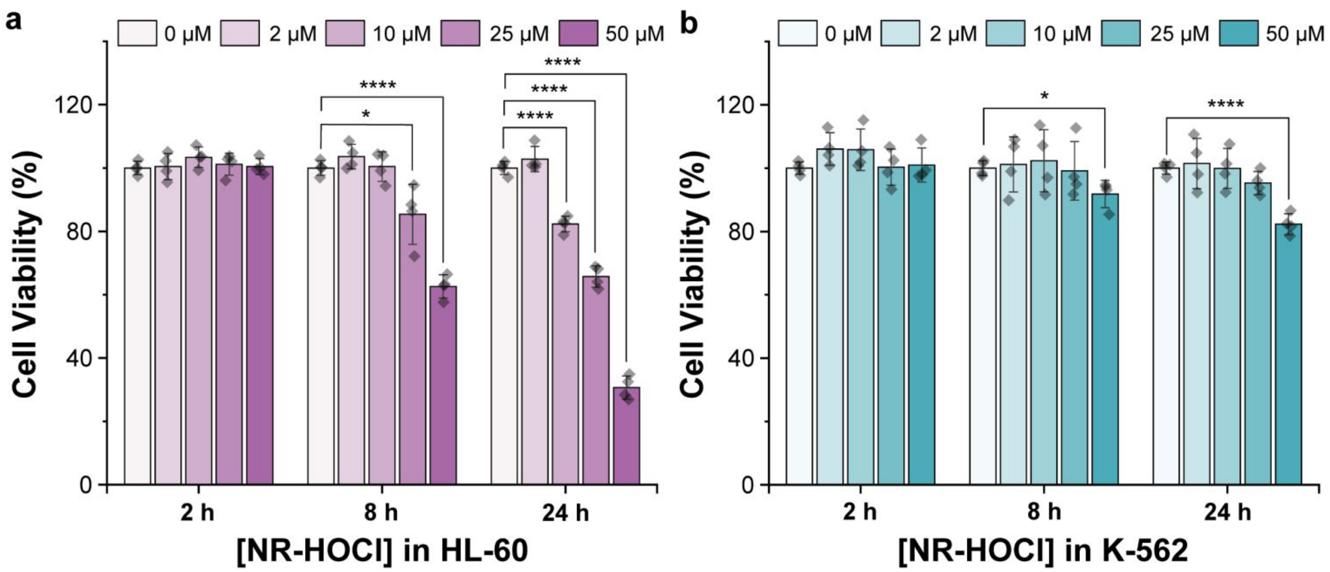

Gated production of **NR$_{666}$-MSA** is toxic to HL-60 cells. Toxicity of **NR-HOCl** in HL-60 (**a**) or K-562 (**b**) cells. Cells were incubated with the indicated concentration of **NR-HOCl** (containing 1% DMF) in the corresponding cell culture media without phenol red. Cell viability was assessed at the indicated time point using the commercially available CCK-8 assay. Data represent four biological replicates. Error bars indicate mean ± SD. Statistically significant differences were determined using a two-tailed t-test and are indicated as * for $p \le 0.05$ and **** for $p \le 0.0001$.

Figure S25

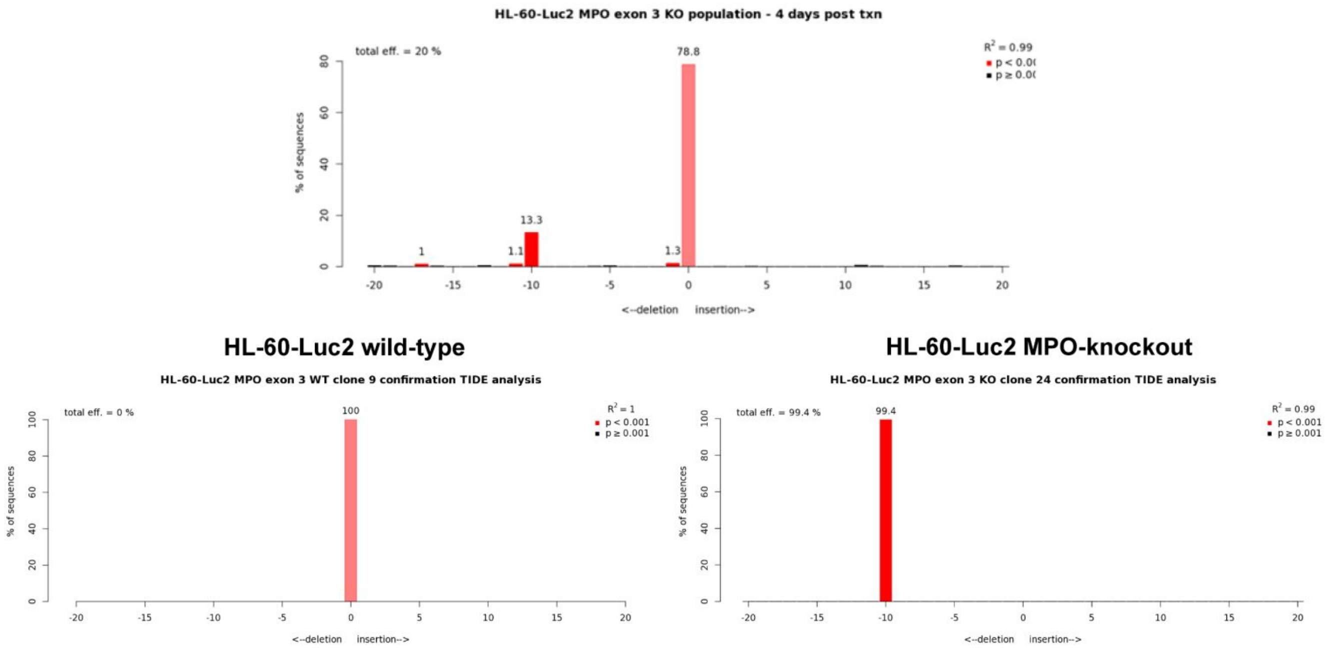

CRISPR/Cas9-mediated MPO gene editing efficiency in HL-60-Luc2 cells assessed by TIDE analysis. Left is wild-type (clone 9); right is the MPO-knockout (clone 24).

Figure S26

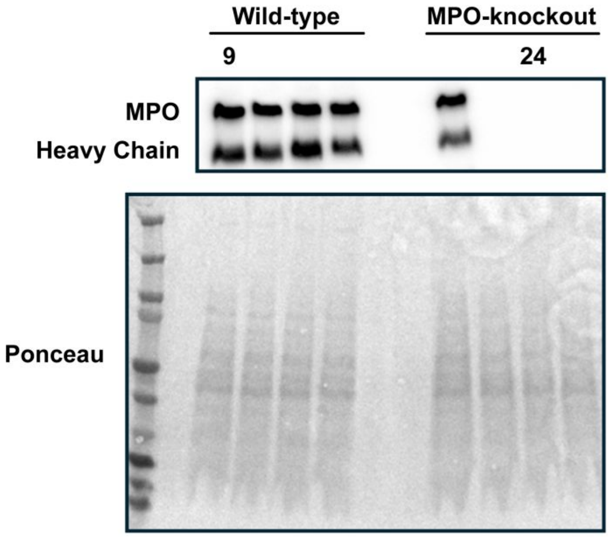

Western blot analysis of MPO expression in HL-60-Luc2 wild-type (clone 9) and HL-60-Luc2 MPO-knockout (clone 24) cells. Cell lysates from HL-60-Luc2 wild-type or the MPO gene knockout cells were analyzed by Western blot using the Myeloperoxidase [E1E7I] XP® Rabbit mAb (top). Equal loading of total protein was confirmed by Ponceau S staining (bottom). The remaining lanes on the image correspond to other clones that were not pursued in this work.

Figure S27

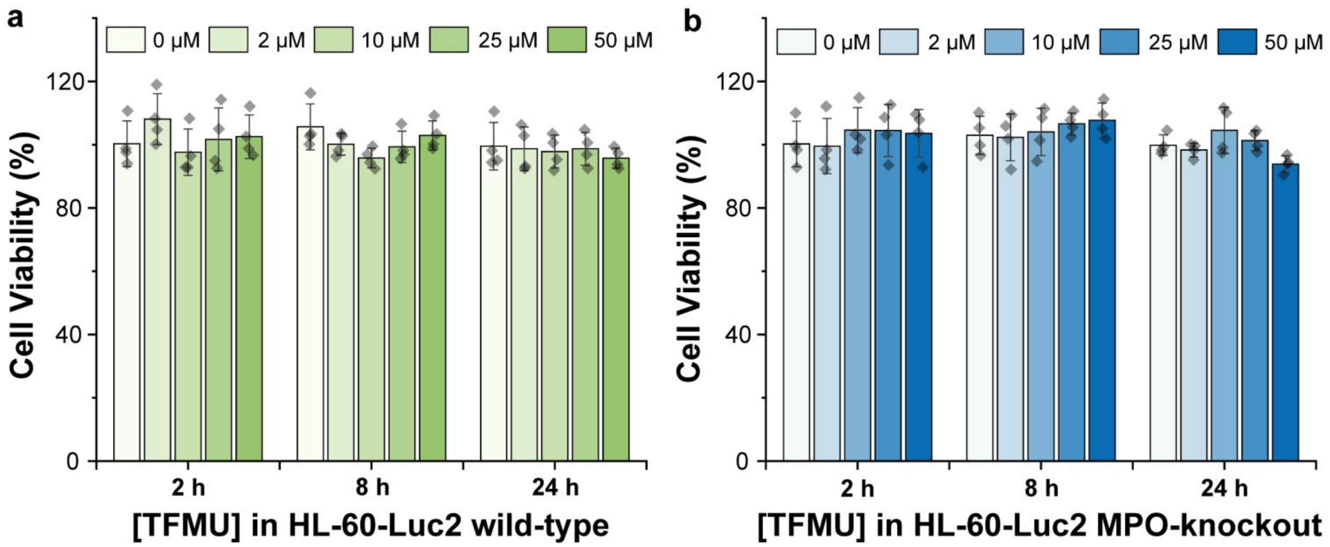

Toxicity of **TFMU** in HL-60-Luc2 wild-type (**a**) or HL-60-Luc2 MPO-knockout (**b**) cells. Cells were incubated with the indicated concentration of **TFMU** (containing 1% DMF) in the corresponding cell culture media without phenol red. Cell viability was assessed at the indicated time point using the commercially available CCK-8 assay. Data represent four biological replicates. Error bars indicate mean ± SD. No statistically significant differences were observed using a two-tailed t-test.

Figure S28

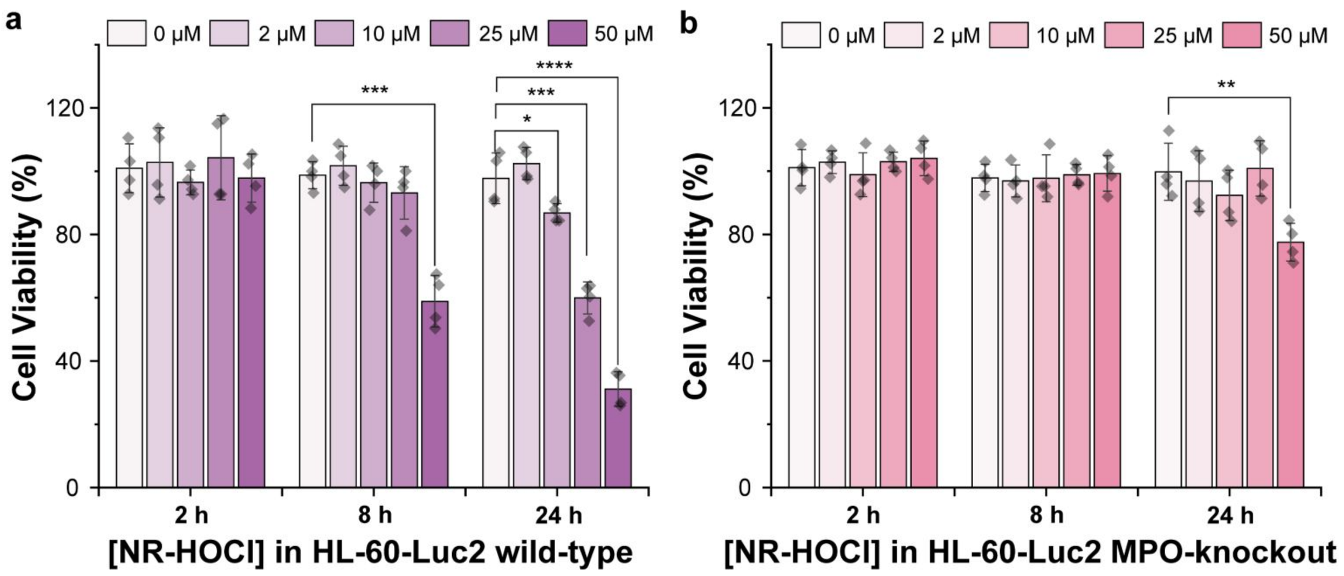

Toxicity of **NR-HOCl** in HL-60-Luc2 wild-type (**a**) or HL-60-Luc2 MPO-knockout (**b**) cells. Cells were incubated with the indicated concentration of **NR-HOCl** (containing 1% DMF) in the corresponding cell culture media without phenol red. Cell viability was assessed at the indicated time point using the commercially available CCK-8 assay. Data represent four biological replicates. Error bars indicate mean ± SD. Statistical significance was determined using a two-tailed t-test and is indicated as follows: * for $p \leq 0.05$, ** for $p \leq 0.01$, *** for $p \leq 0.001$, and **** for $p \leq 0.0001$.

Figure S29

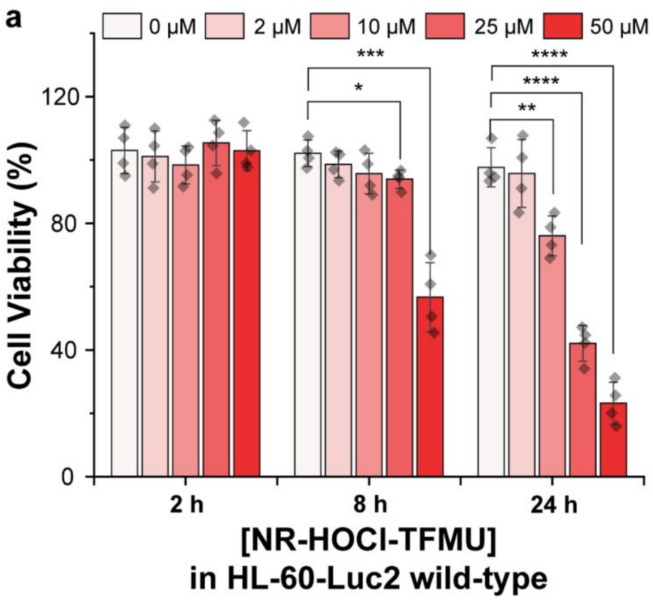

Toxicity of **NR-HOCl-TFMU** in HL-60-Luc2 wild-type (**a**) or HL-60-Luc2 MPO-knockout (**b**) cells. Cells were incubated with the indicated concentration of **NR-HOCl-TFMU** (1% DMF) in the corresponding cell culture media without phenol red. Cell viability was assessed at the indicated time point using the commercially available CCK-8 assay. Data represent four biological replicates. Error bars indicate mean ± SD. Statistically significant differences were determined using a two-tailed t-test and are indicated as * for p ≤ 0.05, ** for p ≤ 0.01, *** for p ≤ 0.001, and **** for p ≤ 0.0001.

Copies of ^{1}H, ^{13}C, ^{19}F and ^{31}P NMR Spectrum

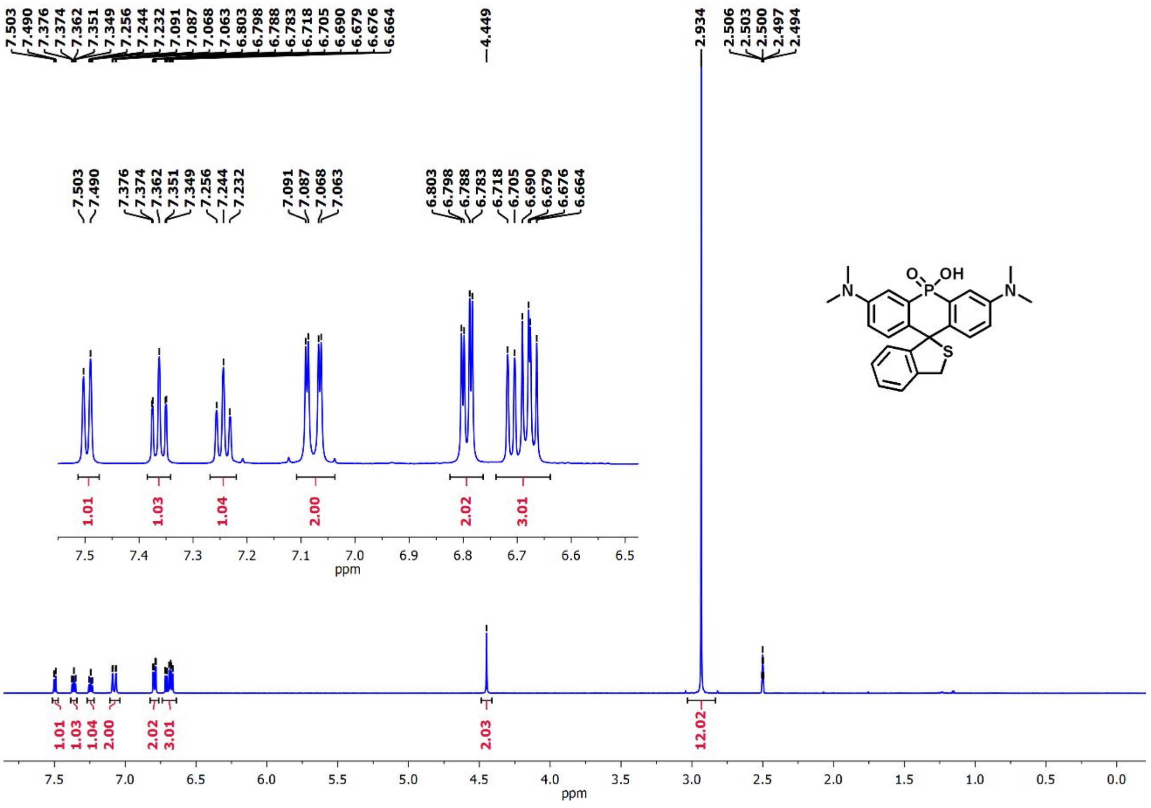

^{1}H-NMR of **NR_{666}-HOCl** in DMSO-d_6 (600 MHz)

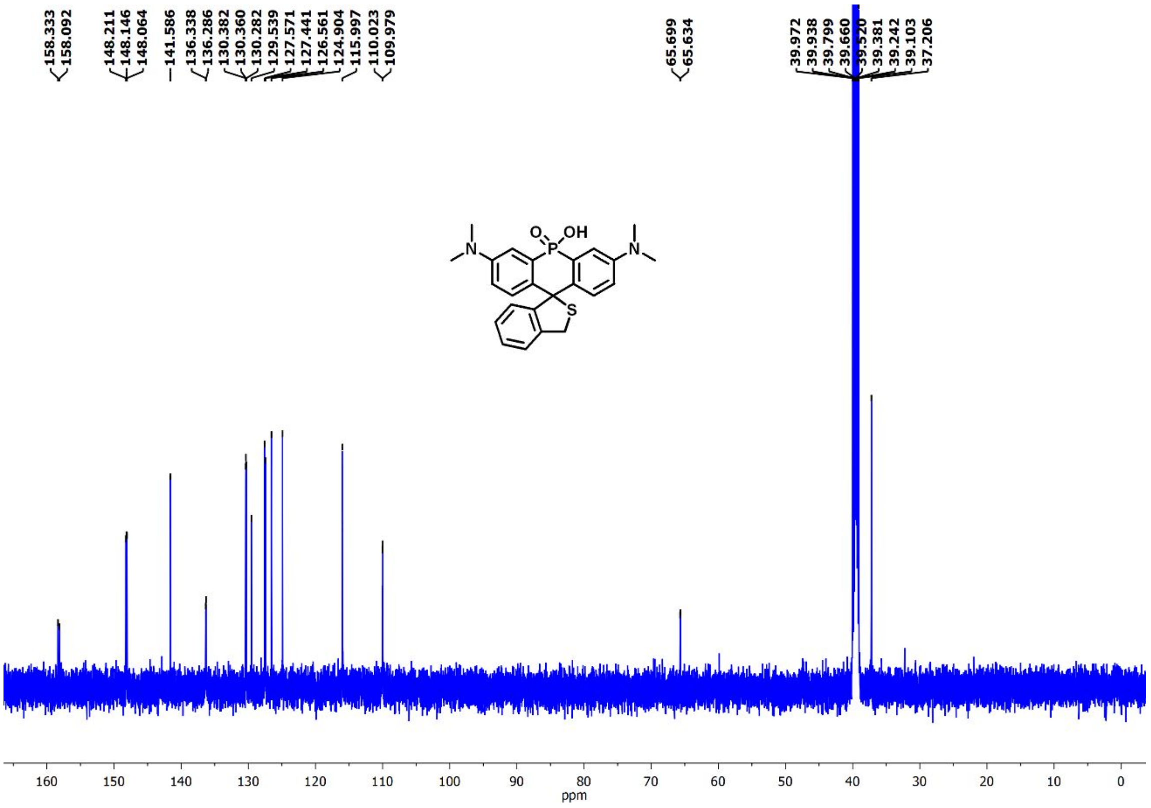

^{13}C-NMR of **NR_{666}-HOCl** in DMSO-d_6 (151 MHz)

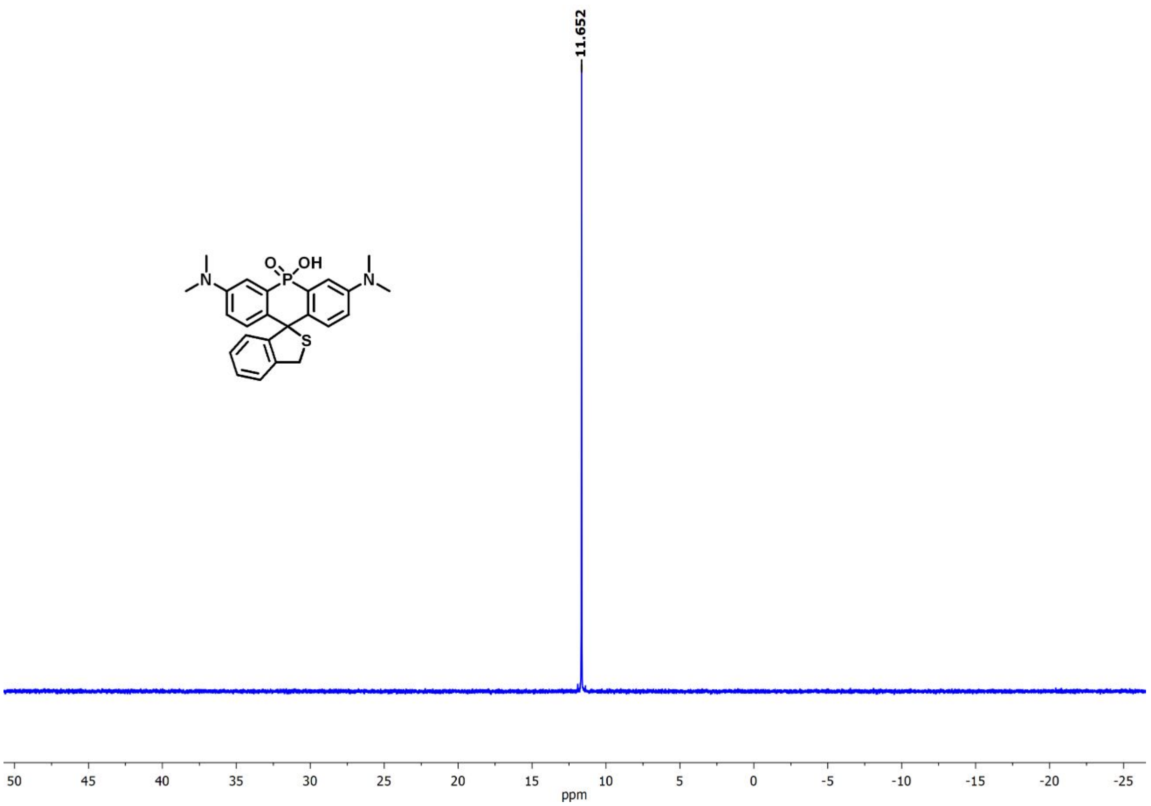

^{31}P-NMR of **NR$_{666}$-HOCl** in DMSO-d_6 (243 MHz)

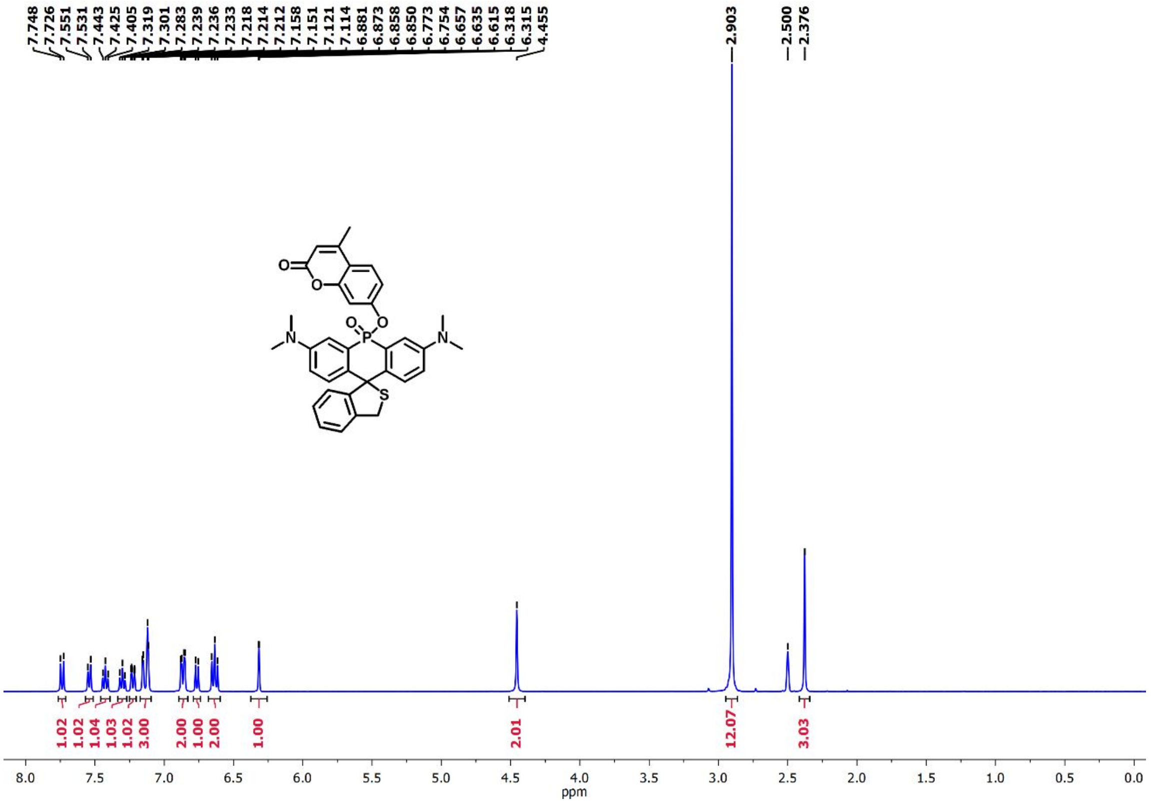

^{1}H-NMR of **NR-HOCl-4MU** in DMSO-d_6 (600 MHz)

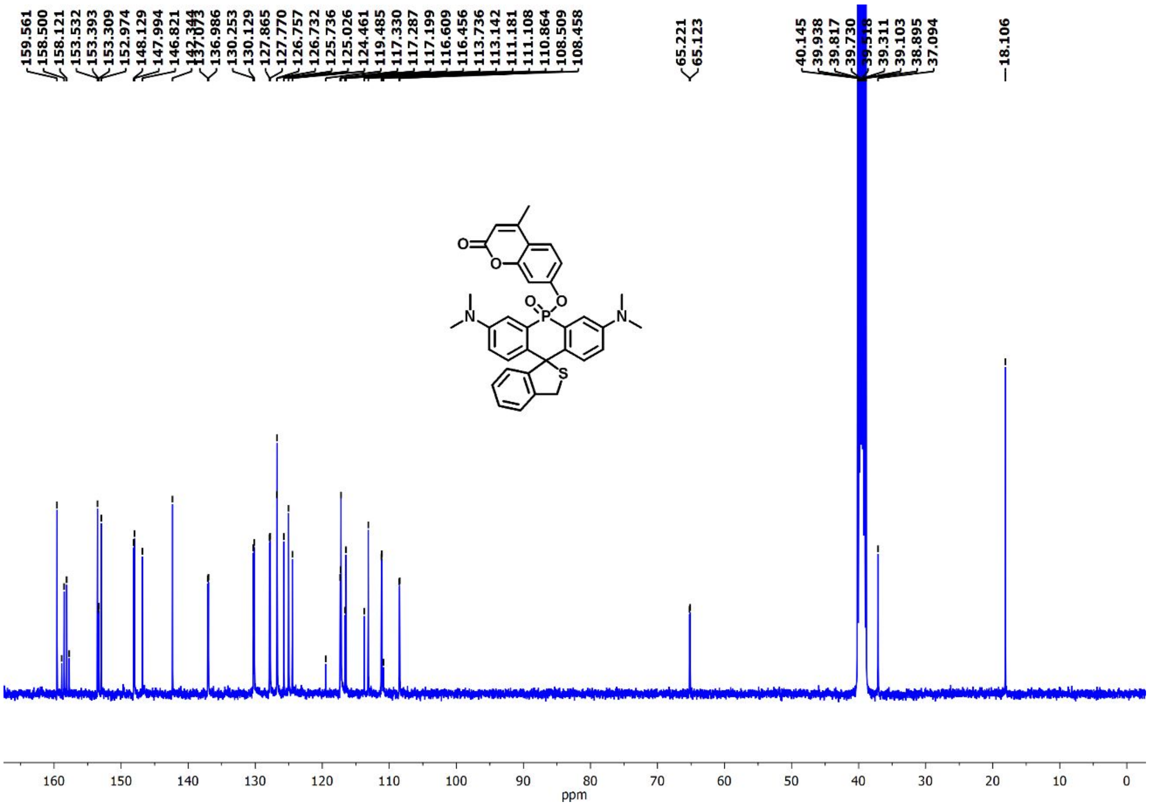

^{13}C-NMR of **NR-HOCl-4MU** in DMSO-d_6 (151 MHz)

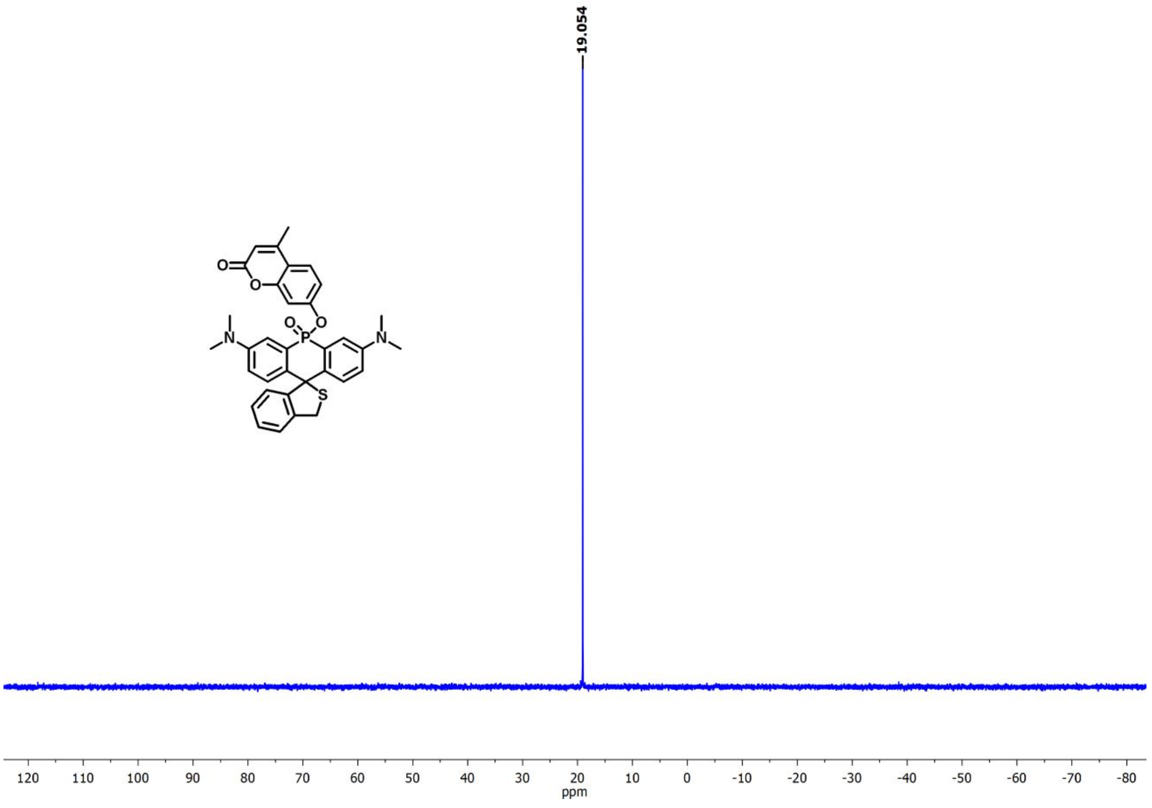

^{31}P-NMR of **NR-HOCl-4MU** in DMSO-d_6 (243 MHz)

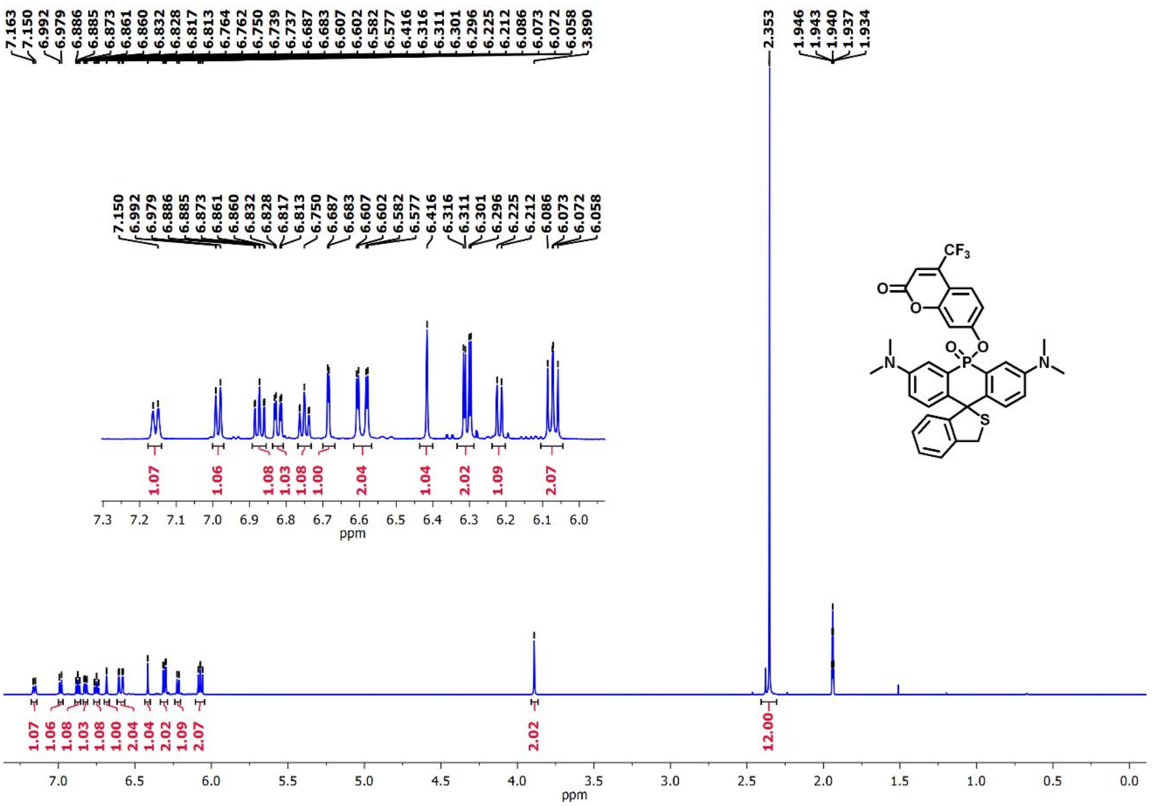

^{1}H-NMR of **NR-HOCl-TFMU** in DMSO-d_6 (600 MHz)

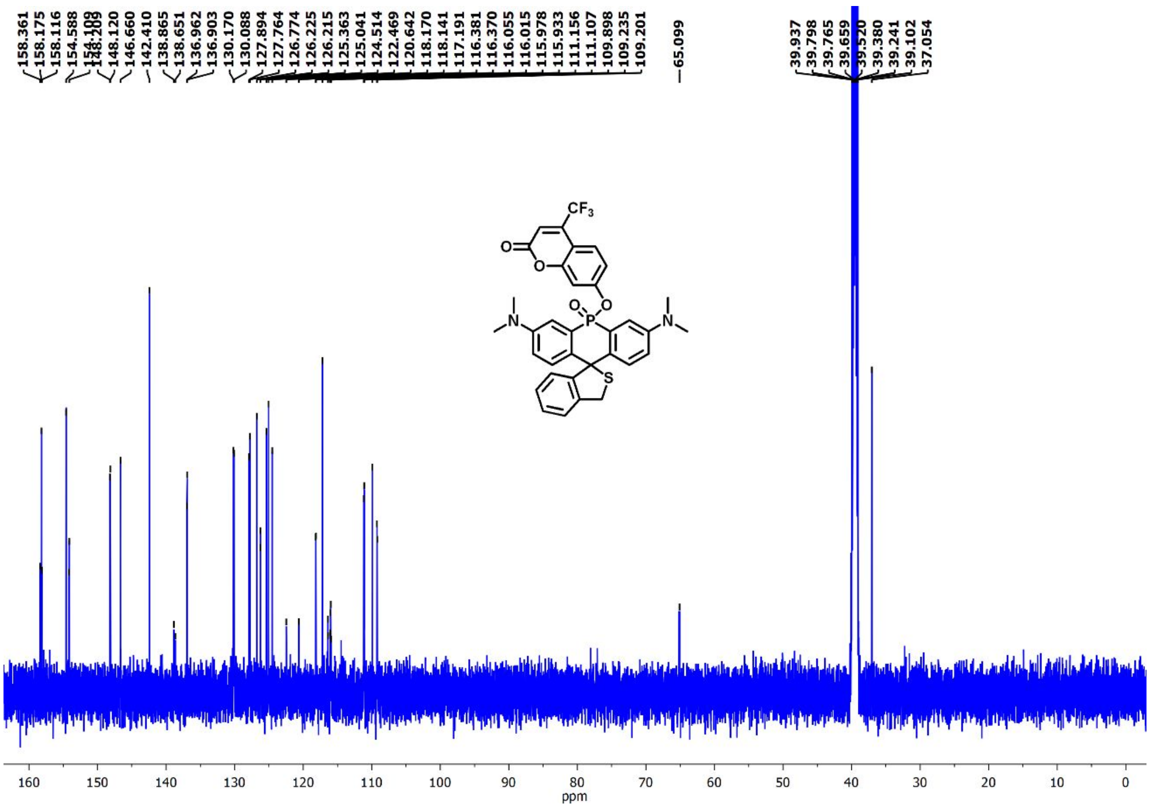

^{13}C-NMR of **NR-HOCl-TFMU** in DMSO-d_6 (151 MHz)

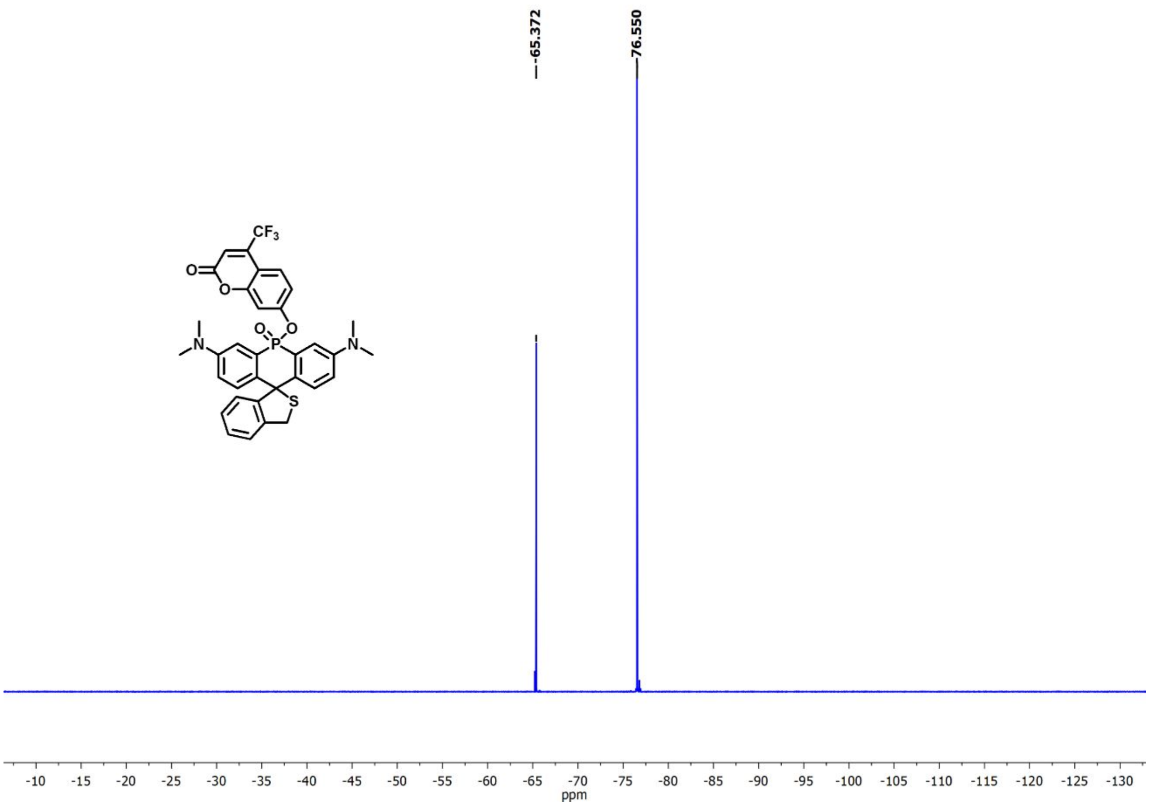

^{19}F-NMR of **NR-HOCl-TFMU** in DMSO-d_6 (564 MHz)

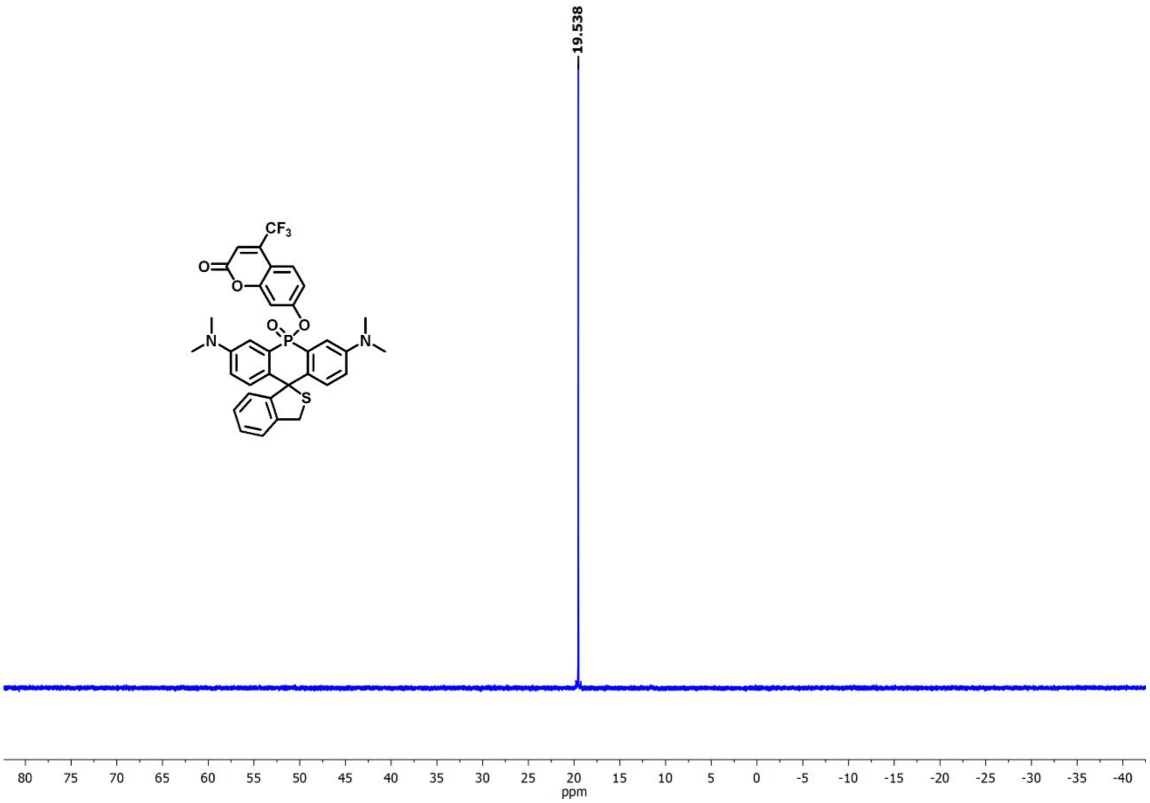

^{31}P-NMR of **NR-HOCl-TFMU** in DMSO-d_6 (243 MHz)

References

1. Mongay, C.; Cerda, V. Britton-Robinson buffer of known ionic strength. *Ann. Chim.*, **1974**, *64*, 409-412.
2. Sun, X.; Xu, Q.; Kim, G.; Flower, S. E.; Lowe, J. P.; Yoon, J.; Fossey, J. S.; Qian, X.; Bull, S. D.; James, T. D. A water-soluble boronate-based fluorescent probe for the selective detection of peroxynitrite and imaging in living cells. *Chem. Sci.*, **2014**, *5*, 3368-3373.
3. Srikun, D.; Miller, E. W.; Domaille, D. W.; Chang, C. J. An ICT-Based Approach to Ratiometric Fluorescence Imaging of Hydrogen Peroxide Produced in Living Cells. *J. Am. Chem. Soc.*, **2008**, *130*, 4596-4597.
4. Zhou, X.; Lai, R.; Beck, J. R.; Li, H.; Stains, C. I. Nebraska Red: a phosphinate-based near-infrared fluorophore scaffold for chemical biology applications. *Chem. Commun.*, **2016**, *52*, 12290-12293.
5. Xiong, B.; Hu, C.; Li, H.; Zhou, C.; Zhang, P.; Liu, Y.; Tang, K. CDI-promoted direct esterification of P(O)-OH compounds with phenols. *Tetrahedron Lett.*, **2017**, *58*, 2482-2486.
6. Koide, Y.; Urano, Y.; Hanaoka, K.; Terai, T.; Nagano, T. Development of an Si-rhodamine-based far-red to near-infrared fluorescence probe selective for hypochlorous acid and its applications for biological imaging. *J. Am. Chem. Soc.*, **2011**, *133*, 5680-5682.
